# Reporting of cluster randomised crossover trials: extension of the CONSORT 2010 statement with explanation and elaboration

**DOI:** 10.1136/bmj-2024-080472

**Published:** 2025-01-06

**Authors:** Joanne E McKenzie, Monica Taljaard, Karla Hemming, Sarah J Arnup, Bruno Giraudeau, Sandra Eldridge, Richard Hooper, Brennan C Kahan, Tianjing Li, David Moher, Elizabeth L Turner, Jeremy M Grimshaw, Andrew B Forbes

**Affiliations:** 1 Methods in Evidence Synthesis Unit, School of Public Health and Preventive Medicine, Monash University, Melbourne, VIC 3004, Australia; 2 Clinical Epidemiology Programme, Ottawa Hospital Research Institute, Ottawa, ON, Canada; 3 School of Epidemiology and Public Health, University of Ottawa, Ottawa, ON, Canada; 4 Institute of Applied Health Research, University of Birmingham, Birmingham, UK; 5 University of Tours, University of Nantes, INSERM SPHERE U1246, Tours, France; 6 INSERM CIC, CHRU de Tours, Tours, France; 7 Wolfson Institute of Population Health, Queen Mary University of London, London, UK; 8 MRC Clinical Trials Unit at University College London, London, UK; 9 Department of Ophthalmology, School of Medicine, University of Colorado Anschutz Medical Campus, Aurora, CO, USA; 10 Centre for Journalology, Clinical Epidemiology Programme, Ottawa Hospital Research Institute, Ottawa, ON, Canada; 11 Department of Biostatistics and Bioinformatics, Duke University, Durham, NC, USA; 12 Duke Global Health Institute, Duke University, Durham, NC, USA; 13 Department of Medicine, University of Ottawa, Ottawa, ON, Canada; 14 Biostatistics Unit, School of Public Health and Preventive Medicine, Monash University, Melbourne, VIC, Australia

## Abstract

This article presents the CONSORT (consolidated standards of reporting trials) extension for cluster randomised crossover trials. A cluster randomised crossover trial involves randomisation of groups of individuals (known as clusters) to different sequences of interventions over time. The design has gained popularity in settings where cluster randomisation is required because it can largely overcome the loss in power due to clustering in parallel cluster trials. However, the design has many methodological complexities, requiring tailored reporting guidance. The guideline was developed using a survey and in-person consensus meeting, informed by a systematic review examining the quality of reporting in cluster randomised crossover trials and relevant CONSORT statements for individual, crossover, cluster, and stepped wedge designs. This article also provides recommended reporting items, along with explanations and examples.

Complete and accurate reporting of randomised trials is necessary to enable users to understand the benefits and harms of an intervention, details of the intervention (such that it could be implemented in practice), the population to which the findings apply, and any methodological limitations that might threaten the validity of the findings. Additionally, complete reporting allows the findings of a trial to be incorporated in evidence synthesis products such as systematic reviews and clinical practice guidelines. Complete and accurate reporting also facilitates reproducibility.^1^ The CONSORT (consolidated standards of reporting trials) statement was first published in 1996^2^ and last updated in 2010.^3^ It aims to improve the quality of reporting of parallel group, individually randomised designs through the provision of a minimum set of recommendations for reporting (hereafter referred to as CONSORT 2010). Extensions have been developed, providing modified and additional recommendations for variants of randomised trial designs (eg, crossover randomised trials,^4^ cluster randomised trials^5^), specific interventions (eg, social and psychological interventions 6), and outcome types (eg, harms^7^).

The cluster randomised crossover (CRXO) trial is a particular type of randomised trial design that has become more prominent in recent decades.^8^ In this design, groups of individuals (known as clusters) are randomly assigned to different sequences of treatments over time (box 1). The design has several potential benefits. The cluster randomisation feature allows evaluation of interventions directed at clusters, can provide logistical benefits, and can reduce the potential for contamination within clusters.^10^ The crossover feature can yield increased statistical efficiency and reduce the impact of chance imbalances in cluster characteristics.^11^

Box 1 **Example of a cluster randomised crossover trial: the PEPTIC trial^9^**

**Primary aim**

To compare proton pump inhibitors versus histamine-2 receptor blockers for stress ulcer prophylaxis on in-hospital mortality among adults requiring mechanical ventilation.

**Primary outcome**

In-hospital all cause mortality up to 90 days from the date of admission.

**Treatment conditions**

Proton pump inhibitors versus histamine-2 receptor blockers.

**Clusters**

Intensive care units.

**Participants**

Adult patients requiring invasive ventilation within 24 hours of admission to the intensive care unit.

**Design**

Two period, two sequence, cross sectional design including 50 intensive care units across five countries. Twenty five intensive care units were randomised to use proton pump inhibitors in the first period, then crossover to use histamine-2 receptor blockers in the second period; the other 25 units were randomised to use histamine-2 receptor blockers in the first period, and then crossover to use proton pump inhibitors in the second period. The trial was pragmatic in its intent to inform clinical decision making, and as such, while the default prescription for stress ulcer prophylaxis was determined by the intensive care unit’s treatment allocation in a particular period, clinicians were able to use the alternative treatment if they considered this preferable. Each period was six months long. There was no washout between periods.

While the CRXO design offers many advantages, methodological complexities exist in the design, conduct, and analysis that require tailored reporting guidance. Current guidelines for the (1) individual parallel group^3^ and crossover designs^4^ and (2) cluster parallel group^5^ and stepped wedge designs^12^ all provide reporting recommendations of relevance to the CRXO design, but these recommendations are scattered across the statements and do not address all issues unique to this design. Furthermore, according to a systematic review examining the quality of reporting of CRXO trials, incomplete reporting was common for aspects of the methods and results.^8^ ^13^ Thus, our aim was to develop a CONSORT reporting extension (CONSORT CRXO) for this specific design that consolidated reporting recommendations across relevant statements, modified items as necessary, and added relevant items to address identified reporting gaps. Here, we report the consensus process used to develop the extension, and the resulting items with explanation and elaboration, along with examples of complete reporting.

**Summary points**

Cluster randomised crossover trials have been gaining popularity recently, owing to the design’s methodological strengths as compared with other cluster randomised designs (ie, increased statistical efficiency, reduced risk of chance imbalances in cluster characteristics)
The design has allowed randomised evaluation of interventions directed at clusters that otherwise could not have been undertaken owing to the availability of too few clusters to achieve the desired power (eg, a limited number of intensive care units within a country)
Many methodological complexities exist in the design, conduct, and analysis of these trials, which require tailored reporting guidance
This article reports on a CONSORT (consolidated standards of reporting trials) extension for the cluster randomised crossover trial design; the reporting guideline brings together advice from relevant CONSORT statements and addresses issues unique to this trial design.
New items and amendments to other items reflect evolving requirements of the scientific community
The extension includes a checklist of 28 items (with 43 subitems), along with a separate checklist for journal and conference abstracts, and sample size items

## Design features of a cluster randomised crossover trial

In a CRXO design, clusters are randomly allocated to sequences of treatments that are delivered in different periods of time (see table 1 for a glossary of terms). Typically, the sequences alternate between intervention and control conditions, such that each cluster will cross between all treatment conditions at least once. Some trials include a washout period when no treatment is delivered, as a means to mitigate any potential carryover effects at the individual or cluster level.

**Table 1:** Glossary

Term	Explanation
Carryover effect	Effect of the treatment delivered in one period persisting into a subsequent period.
Cell	Combination of the sequence and period. See sequence period.
Cluster period	Combination of a cluster and period (eg, standard design of a cluster randomised crossover trial has two cluster periods for each cluster).
Cohort*	Participants are repeatedly assessed over a series of measurement points. In an open cohort design, participants can join and leave the trial throughout its duration; in a closed cohort design, participants cannot join the trial once it has started.
Cross sectional*	When different participants are measured at each measurement occasion.
Estimand	A clear description of what the treatment effect being estimated represents.
Exposure to treatment conditions	Exposure refers to a participant potentially being affected by an intervention or control condition, whether directly (eg, being given an individual level intervention) or indirectly (eg, through an intervention delivered at the cluster level).
Gatekeepers, cluster guardians	Individuals or bodies (such as a school principal or municipal council) who may be called on to protect the interests of organisations or communities that are the setting for a cluster randomised trial.^14^
Dual pair sequences (duals)	A pair of sequences that are the inverse of one another (eg, ABB and its dual BAA).^11^ Designs consisting of sequences and their duals help to ensure a time balanced design.
Multiple period, or longitudinal, cluster randomised crossover design	Where clusters cross between treatments more than once.^11^
Period*	Grouping of observations by time of measurement.
Period effect	Systematic differences in responses across periods that are not due to the treatments.^15^ Examples of period effects include seasonal or secular trends, and order and learning effects (see item 3a for specific examples).
Participant*	An individual who investigators seek to measure the outcome of interest.
Research participant*	An individual participating in research from the standpoint of ethical considerations.
Sequence (or sequence of treatments)†	Order in which a cluster will receive the treatment conditions over time. More than one cluster can be allocated to each sequence.
Sequence period	Combination of the sequence and period. For example, a standard design in a cluster randomised crossover trial has four sequence periods in total from sequence AB (period 1, treatment condition A; period 2, treatment condition B) and sequence BA (period 1, treatment condition B; period 2, treatment condition A).
Standard cross sectional design for cluster randomised crossover trials	Design for a cluster randomised crossover trial with two periods, two treatments (A or B), and two sequences (AB, BA), with different participants in each period.
Washout period	Length of time between treatment periods when no treatment is delivered to reduce the risk of carryover effects.

* Definition used in, or with minor modification from, the CONSORT extension for stepped wedge trials.^12^

† Following the CONSORT extension for stepped wedge trials, we avoid the terms “group” or “arm” to refer to the allocated treatment as is used in the standard CONSORT statement. We instead use the term “treatment” in a generic way to refer to either the active treatment or comparator. We use the phrase “intervention condition” to refer to the active treatment and the “control condition” to the comparator.

**Fig 1:**
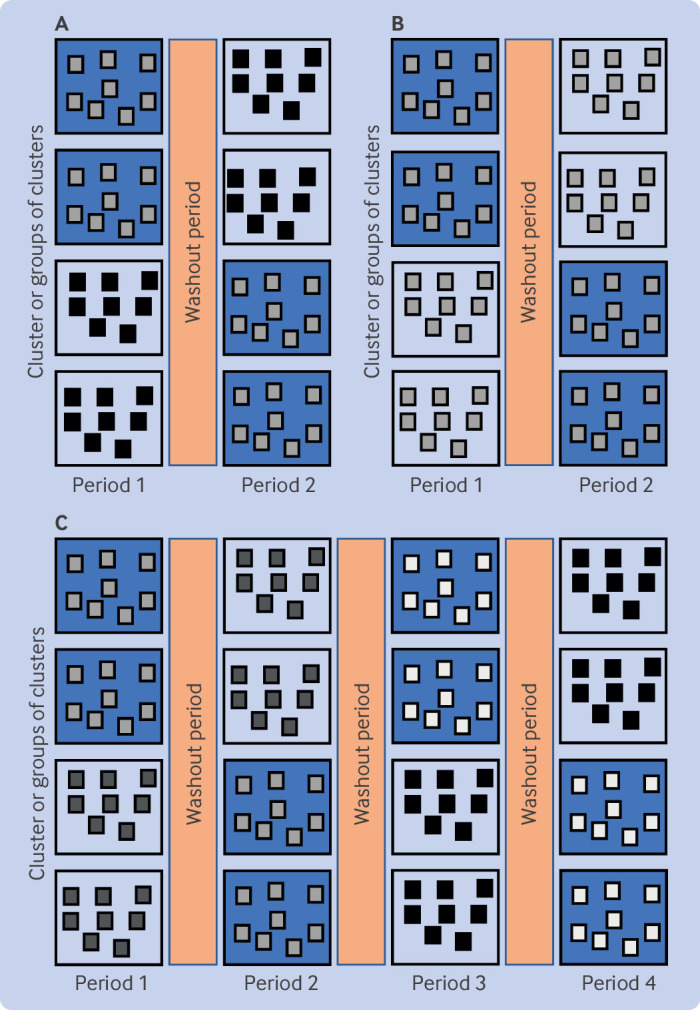
Three different designs for cluster randomised crossover trials. Large boxes represent clusters (or groups of clusters), with different colours representing different treatments. Internal boxes represent participants, with changes in grey shading from period to period representing different participants across periods (ie, cross sectional design). (A) Standard cross sectional design (indicated by different grey shading of internal boxes across periods) with two treatments, two periods, two sequences (AB, BA). (B) Standard cohort design (indicated by the same grey shading of internal boxes across periods) with two treatments, two periods, two sequences (AB, BA). (C) Cross sectional design with two treatments, four periods, two sequences (ABAB, BABA)

Figure 1A depicts the commonly used two treatment (A or B), two period, and two sequence (AB or BA) cross sectional design, where observations in different periods in a cluster are of different people.^8^ Figure 1B depicts a cohort design, where observations in different periods in a cluster are of the same people. Mixture designs are also possible in which observations are from a mix of the same and different people in different periods. Figure 1C extends the first design by increasing the number of periods (from two to four), but still has two treatments (A or B) and two sequences (ABAB, BABA). Designs in which clusters cross between treatments more than once are known as multiple period or longitudinal cluster designs.^11^

The CRXO design shares features in common with parallel group cluster trials (where clusters are randomised to treatment conditions, which they remain in for the duration of the study), individually randomised crossover trials (where individuals are randomised to sequences of treatments), and stepped wedge trials (where clusters are randomised to sequences of treatments, but where the clusters can only cross in one direction (eg, from A to B).^16^ ^17^ For this reason, the CRXO design shares some of the potential benefits and limitations of these designs.

## Development of the CONSORT CRXO extension

**Fig 2:**
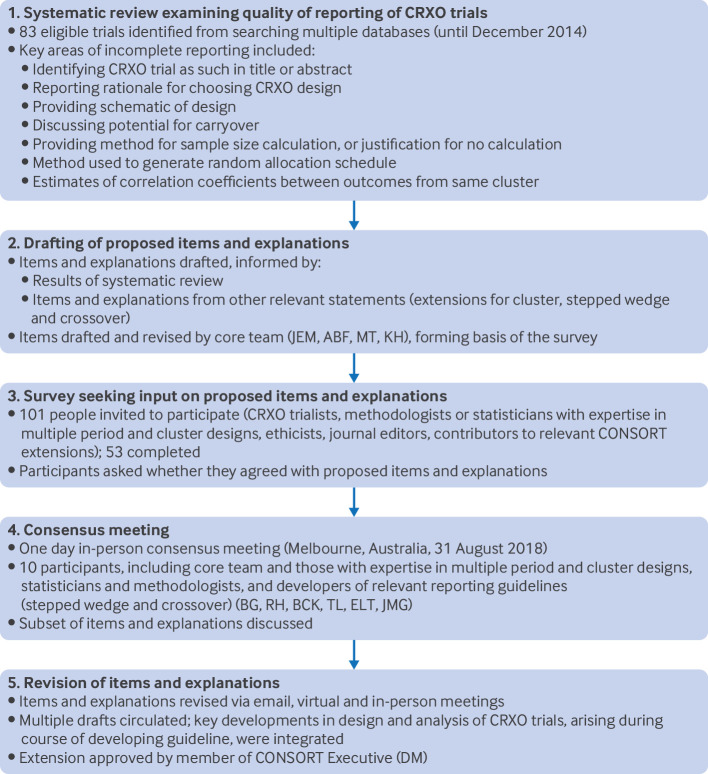
Summary of five step process used to develop the CONSORT CRXO extension for cluster randomised crossover trials (CRXO). Further details are available in supplementary materials 1

Development of this extension was based on guidance for developing health research reporting guidelines.^18^ We registered our intent to develop the CONSORT CRXO extension on the EQUATOR (enhancing the quality and transparency of health research) Network (23 June 2017). Figure 2 summarises the development process, and supplementary materials 1 provides details.

## CONSORT CRXO extension

### Scope of the guideline

This CONSORT extension has been developed for CRXO designs in which clusters of participants are randomly allocated to sequences of treatments over time, where the sequences alternate between intervention and control conditions (eg, AB, BA). However, much of the extension applies to a broader range of designs where at least some of the clusters cross forwards and others backwards at least once between treatments (eg, AA, AB, BA, BB). Trials are excluded if crossing between interventions arises solely from protocol deviations. One way crossover (or cross forward) designs where clusters can only cross in one direction (eg, from A to B) are also excluded.^17^ A prominent subset of these cross forward designs is the stepped wedge design, for which a CONSORT extension is available.^12^

### How to use the CONSORT CRXO extension

The checklist includes 28 items (with 43 subitems; table 2), along with a separate checklist for journal and conference abstracts (table 3), and sample size items (table 4). Some items are identical to those in the CONSORT 2010 statement or relevant extensions, while others have been modified or are new (see supplementary materials 2, tables S1 and S2 for details, along with evidence of reporting quality for items in table S3). New items on research ethics review (item 26), data sharing (item 27) and patient and public involvement (item 28) have been included. In line with recommendations to provide greater clarity of the treatment effects being estimated, we incorporate estimands into item 12a.^23^ ^24^ For CRXO trials, items in this extension should be addressed, replacing those in the CONSORT 2010 statement.

**Table 2:** Checklist of information to include when reporting a cluster randomised crossover (CRXO) trial

Section or topic	Item No	Checklist item
**Title and abstract**		
	1a	Identification as a cluster randomised crossover trial in the title.
	1b	Structured summary of the trial design, methods, results, and conclusions (see separate CRXO checklist for abstracts).
**Introduction**		
Background and objectives	2a	Scientific background and explanation of rationale.
	2b	Specific objectives or hypotheses.
**Methods**		
Trial design	3a.1	Rationale for a cluster crossover design.
	3a.2	Description of the realised trial design including: number of treatment conditions; definition of cluster (ie, the unit of randomisation); number and duration of periods; number and composition of sequences (eg, ABAB, BABA); number of clusters randomised to each sequence; duration of any washout periods; whether the participants assessed in different periods are the same people, different people, or a mixture of the two; and consideration of potential for carryover effects. A diagram of the trial is recommended when there are more than two periods and/or treatment conditions.
	3b	Important changes to planned methods after trial commencement (such as eligibility criteria), with reasons.
Participants	4a	Eligibility criteria for clusters and participants.
	4b	Settings and locations where the data were collected.
Intervention	5	The treatment conditions with sufficient details to allow replication, and whether they were delivered at the level of the cluster, the individual, or both.
Outcomes	6a	Completely defined prespecified primary and secondary outcome measures, including how and when they were assessed (for specific guidance, see CONSORT for outcomes).
	6b	Any changes to trial outcomes after the trial commenced, with reasons.
Sample size	7a	How sample size was determined. Method of calculation and relevant parameters with sufficient detail so the calculation can be reproduced. Assumptions made about correlations between outcomes of participants from the same cluster (see separate CRXO checklist for sample size items).
	7b	When applicable, explanation of any interim analyses and stopping guidelines.
Randomisation:		
Schedule generation	8a	Method used to generate the random allocation schedule.
	8b	Type of randomisation; details of any restricted randomisation, if used.
Allocation concealment mechanism	9	Specification that allocation was based on clusters; description of any methods used to conceal the allocation from the clusters until after their recruitment.
Implementation	10a	Who generated the random allocation schedule, who enrolled clusters, and who assigned clusters to sequences of treatments in the schedule.
	10b	Mechanism by which individual participants were included in clusters for the purposes of the trial (such as complete enumeration or random sampling; continuous recruitment or ascertainment, or recruitment at a fixed point in time), including who recruited or identified participants.
	10c	Whether consent was sought, from whom, when, and for what; whether this differed between treatment conditions. Justification for any waiver or modification of informed consent requirements.
Blinding	11a	Who was blinded after assignment to sequences (eg, cluster level participants, individual level participants, those assessing outcomes) and how.
	11b	If relevant, description of the similarity of interventions.
Statistical methods	12a	Target estimand for each primary and secondary outcome including whether it pertains to the cluster level or individual level; statistical methods for their estimation including how period effects, clustering, and repeated measures were taken into account. Any assessment of carryover effects should be reported.
	12b	Methods for additional analyses, such as subgroup analyses, sensitivity analyses, and adjusted analyses.
**Results**		
Participant flow (a diagram is strongly recommended)	13a	The numbers of clusters that were assessed for eligibility and were randomly assigned to each sequence. For each sequence period (ie, each cell) or treatment condition: the numbers of clusters that received intended treatments and were analysed for the primary outcome; and the numbers of participants who were assessed for eligibility, received intended treatments and were analysed for the primary outcome.
	13b	For each sequence period (ie, each cell) or treatment condition, losses and exclusions for both clusters, and participants with reasons.
Recruitment	14a	Dates of treatment periods and washout periods.
	14b	Why the trial ended or was stopped.
Baseline data	15	A table showing baseline cluster level characteristics by sequence, and individual level characteristics for each sequence period (ie, each cell) or treatment condition.
Numbers analysed	16	The number of observations and clusters included in each analysis for each treatment condition and whether the analysis was according to the allocated schedule.
Outcomes and estimation	17a	For each primary and secondary outcome, summary statistics by sequence period (ie, each cell) or treatment condition; the estimated effect size and its precision (eg, 95% confidence interval); and any within-cluster correlations or variance components estimated in the analysis.
	17b	For binary outcomes, presentation of both absolute and relative effect sizes is recommended.
Ancillary analyses	18	Results of any other analyses performed, including subgroup analyses, sensitivity analyses, and adjusted analyses, distinguishing prespecified from exploratory.
Harms	19	Important harms or unintended effects in each treatment condition (for specific guidance, see CONSORT Harms 2022 statement).
**Discussion **		
Limitations	20	Trial limitations, addressing sources of potential bias, imprecision, and if relevant, multiplicity of analyses. Consider potential carryover effects.
Generalisability	21	Generalisability (external validity, applicability) of the trial findings. Generalisability to clusters or individual participants, or both (as relevant).
Interpretation	22	Interpretation consistent with results, balancing benefits and harms, and considering other relevant evidence.
**Other information **		
Registration	23	Registration number and name of trial registry, or state the trial was not registered.
Protocol	24	Where the full trial protocol and statistical analysis plan can be accessed, if available.
Funding	25	Sources of funding and other support (such as supply of drugs), role of funders.
Research ethics review	26	Whether the study was approved by a research ethics committee, with identification of the review committee(s).
Data sharing	27	Where the individual de-identified participant data (including data dictionary), statistical code, and any other relevant documents or materials can be accessed.
Patient and public involvement	28	Details of any patient and/or public involvement in the design, conduct and reporting of the trial; and, when applicable, other stakeholders’ involvement.

CONSORT=consolidated standards of reporting trials.

Checklist can be downloaded as a separate document in supplementary materials 4.

**Table 3:** Items to report in conference and journal abstracts of a cluster randomised crossover (CRXO) trial

Abstract item	Extension for CRXO trials
Title	Identification of study as a CRXO trial
Trial design	Description of the trial design (including number of sequences, periods and clusters; and whether participants assessed in different periods are the same people, different people, or a mixture)
Methods	
Participants	Eligibility criteria for clusters and participants and the settings where the data were collected
Interventions	The treatment conditions
Objective	Specific objectives or hypotheses
Outcome	Clearly defined primary outcome
Randomisation	How clusters were allocated to sequences of treatments
Blinding (masking)	Whether or not participants, healthcare professionals, those recruiting and those assessing outcomes were blinded
Results	
Numbers randomised	Number of clusters randomised to each sequence of treatments
Recruitment	Trial status
Numbers analysed	Number of clusters included in the analysis; number of observations in the analysis for each treatment condition
Outcome	For the primary outcome, summary statistics by treatment condition and the estimated effect size (confidence interval)
Harms	Important adverse events or side effects (even if not observed), or that harms were not assessed
Conclusions	General interpretation of the results
Trial registration	Registration number and name of trial register
Funding	Source of funding
Research ethics review	Ethical approvals

**Table 4:** Core items to report under the sample size calculation (item 7a)* for a cluster randomised crossover (CRXO) trial

Recommended items to report	Further explanation
Outcome(s)	Outcome(s) for which the sample size calculation was undertaken.
Target difference	For a superiority trial, the magnitude of difference in the outcome of interest (effect size) that the trial is designed to detect (see Power), along with the basis for this choice. Specify the target difference as an absolute or relative effect, or both (eg, risk difference, risk ratio).^19^ For equivalence and non-inferiority trials, as above, but where the target difference is replaced by the equivalence or non-inferiority margin.
Level of significance (probability of a type I error)	State clearly whether a one sided or two sided test was used.
Power	The probability of detecting the target difference (or larger) as statistically significant, given such a difference exists.
A measure of variation for the outcome	For continuous outcomes, a standard deviation is required; for binary outcomes, the control condition proportion should be specified. For other outcomes, see Cook et al.^19^
Number of clusters and allocation ratio	The total number of clusters and the allocation ratio of the sequences (ie, the proportion of clusters allocated to each sequence).
Number of periods	State the number of periods.
Average cluster size	The total cluster size or the cluster size per period, clearly denoting which is reported.
Assumed correlation structure	The assumed ICC and whether the ICC is time dependent or time independent. If time dependent, state the parameters that were assumed to accommodate the time dependency, for example, the within-period ICC and the between-period ICC or the cluster autocorrelation coefficient, or any variance components. For binary outcomes, it is important to report the scale of the correlations or variance components (eg, proportions scale or logistic scale). For rate outcomes, it might be more appropriate to report coefficients of variation of outcomes.^12^
Within-person correlations	Where the design includes repeated measurements on the same individual, describe the assumed correlation structure at the individual level (eg, an individual autocorrelation coefficient), including if any decay in correlation in repeated measures on the same individual has been accounted for.
Method used	Description of the sample size methodology used (eg, via provision of a formula, or results and code for simulation based, sample size calculations), including the statistical packages (and functions) used for implementation.
Any adjustments made	
Allowance for multiple primary outcomes	Any allowance made for multiple primary outcomes (eg, by applying a correction to the level of significance in the sample size calculation^20^).
Allowance for variation in cluster size	Any allowance made for unequal cluster sizes. This can include variation in total cluster sizes or variation in sizes of cluster periods.
Allowance for attrition	Any inflation due to anticipated attrition at the cluster or participant levels, or both.
Small sample adjustment	Any corrections applied when the number of clusters are small. Sample size calculations for CRXO trials based on the normal distribution are only accurate when the number of clusters is large. Use of these formulas when the derived number of clusters is small could underestimate the required number of clusters, resulting in a trial with less power than planned. A range of small sample size adjustments might be applied, such as adding clusters or using values for the t distribution in place of the normal distribution in the sample size calculation^10^ (page 149).^21^ ^22^
Sensitivity analysis	Any analyses undertaken to examine the sensitivity of the sample size, or power, to the chosen parameters (eg, ICCs, control condition proportion). Justification for the chosen parameters should be provided.

CONSORT=consolidated standards of reporting trials; ICC=intracluster correlation coefficient.

* Adapted (where applicable) from table 5 of the CONSORT extension for stepped wedge, cluster randomised trials^12^ and informed by the DELTA^2^ guidance on choosing the target difference.^19^

For each item, the CRXO item, an explanation, and example(s) are presented. For explanations and examples for items 2a, 4b, 6b, 11b, 14b, 21, 22, 23, and 25, readers are referred to the CONSORT 2010 statement. Other CONSORT extensions might also need to be consulted depending on the attributes of the trial (eg, the CONSORT extension for health equity in trials focusing on health equity^25^). The checklist structure guides where to report information, but this structure should not be seen as prescriptive. Most journals offer online supplements (and the availability of open access repositories), so journal space restrictions should not preclude complete and accurate reporting of this minimum set of items.

## Title and abstract

### Item 1a: Title

Identification as a cluster randomised crossover trial in the title.

#### Explanation

Identification of the study design in the title facilitates appropriate indexing in bibliographic databases^3^ for searching and ensures ease of identification of randomised trials for potential inclusion in evidence synthesis products (eg, systematic reviews). Further, alerting readers to the study design early in the article allows them to consider implications for the analysis methods and any potential biases.

#### Example of item 1a

“Cluster-Randomized, Crossover Trial of Head Positioning in Acute Stroke”.^26^

### Item 1b: Abstract

Structured summary of trial design, methods, results, and conclusions (table 3).

#### Explanation

Clear, accurate, and sufficiently detailed abstracts are necessary for several reasons: readers commonly use abstracts to decide whether to read the full article; some readers might not have access to the full article and might base healthcare decisions on the abstract only; in systematic reviews using multistage screening, the eligibility of studies at the first stage of screening is based on abstracts; and finally, to ensure appropriate indexing in electronic databases. A minimum set of items to be reported in an abstract of a CRXO trial is presented in table 3. An adapted version of a published abstract demonstrating reporting against the items is provided (supplementary materials 3, table S1).

## Introduction

### Item 2b: Objective

Specific objectives or hypotheses.

#### Explanation

Specifying objectives allows readers to understand the questions the trial is designed to ask.^27^ In cluster trials, the treatments might be intended to change outcomes at different levels—for example, at the cluster level (eg, clinicians’ adherence to guideline recommendations) or the patient level (eg, mortality), or both. Furthermore, trials might be undertaken for different purposes: to inform clinical decision making (pragmatic intention) or to understand the effects of the treatments in ideal experimental conditions (explanatory intention). Clearly reported objectives will provide clarity about the purpose of the trial and its intended effects. The articulation of research questions might be aided by the use of a question formulation framework^27^; for example, the PICOTS framework (patient/population, intervention, comparison, outcomes, timing, and setting) or the estimands framework (item 12a).

#### Example of item 2b

*Example (reporting of PICOTS elements; pragmatic intention)*—“we conducted a pragmatic, multicenter, cluster randomized, crossover trial. We hypothesized that umbilical cord milking would reduce admission to the neonatal intensive care unit (NICU) compared with early cord clamping in nonvigorous newborns born between 35 and 42 weeks’ gestation.”^28^

## Methods: Trial design

### Item 3a: Trial design

3a.1 Rationale for a cluster crossover design.

3a.2 Description of the realised trial design, including the following:

- number of treatment conditions;
- definition of cluster (ie, unit of randomisation);
- number and duration of periods;
- number and composition of sequences (eg, ABAB, BABA);
- number of clusters randomised to each sequence;
- duration of any washout periods;
- whether the participants assessed in different periods are the same people, different people, or a mixture of the two; and
- consideration of potential for carryover effects.

A diagram of the trial is recommended when there are more than two periods or treatment conditions.

#### Explanation

A rationale for the choice of trial design should be provided. For a CRXO trial, this rationale should include justification of the cluster and crossover aspects of the design.^13^ Justifying cluster randomisation is important because this will likely increase the required number of participants compared with an individually randomised trial. Thus, more participants will likely receive an intervention of unknown effectiveness than if individual randomisation were used. Furthermore, cluster randomisation could increase the potential for bias arising from selective identification and recruitment of participants. The crossover element might exacerbate this risk in cross sectional designs, because participants in the second period are, by definition, recruited after randomisation and it might be harder to ensure that those responsible for recruitment in the second period are unaware of the cluster’s assigned treatment. The crossover element might lead to an increased risk of cluster withdrawal owing to additional burden of participating in multiple periods. Hence, justifying the need for the crossover element is also important.

Possible justifications for adopting a cluster randomised design include the ability to evaluate interventions directed at clusters, operational and logistical benefits, and the need to avoid contamination within clusters (when interventions are delivered at the individual level).^11^ Possible justifications for using the crossover include infeasibility of a parallel design due to the availability of too few clusters to achieve the desired power^29^; reduction of the impact of chance imbalances in cluster characteristics; and increased statistical efficiency (with associated benefits such as minimising the number of participants receiving the interventions, and reducing the cost).

A key requirement of the crossover design is that the effect of an intervention given in one period does not carry over into the next period.^30^ In a CRXO trial, the carryover can occur at both the cluster and individual levels.^31^ At the cluster level, the treatment delivered in one period can continue to be delivered in subsequent periods. At the individual level in cross sectional designs, participants who have treatment for a long period (eg, those receiving care in an intensive care unit) might unintentionally be given the other treatment if they are still in the cluster when it crosses over. In open or closed cohort designs where participants are followed over time, carryover could arise in the same ways as in individually randomised crossover designs (ie, the treatment itself could persist (eg, physical persistence of a drug) or the effects of the treatment might persist (eg, a drug has a curative effect)).^8^ ^15^ ^30^ Therefore, methods for managing potential carryover (eg, the use of washout periods, along with justification for the duration; different participants in each period; and blinding of trialists involved in the delivery of the intervention), or justification for why carryover at both the cluster and individual level is expected to be negligible, should be provided. In a 2×2 crossover design, carryover is particularly problematic because it cannot be distinguished from a treatment by period interaction.^30^

In CRXO trials where period effects are expected, any design approaches used to limit the possible impact of such period effects should be noted. An example of a period effect is the seasonal variation in outcomes that might be expected when the trial spans a year and the outcome (eg, incidence of influenza) varies by season. A learning effect is another form of period effect that might arise when fidelity to each intervention improves similarly in the second period. The use of balanced designs and dual pair sequences (eg, ABB and its dual BAA) could limit the potential for period effects to bias the estimation of treatment effects.^11^

Providing specific details of the CRXO design is important to allow assessment of the potential for bias, and whether the sample size and analysis methods were appropriate. Where possible, justification for the chosen design elements should be provided (eg, providing a rationale for the chosen composition of sequences). A diagram can efficiently and clearly communicate the design details, particularly for complex designs (more than two periods or treatment conditions or both) or simple designs with staggered commencement of clusters. Key details to depict for each sequence include the commencement date and duration of each treatment condition and washout period, and the number of clusters allocated (example 3 of item 3a.2). Such a diagram will clearly signal any imbalance in the treatments evaluated in each period, and therefore the need to control for period effects.

#### Example of item 3a.1

*Example 1 (rationale for cluster randomisation and crossover)*—“Cluster-randomisation with hospitals as clusters was used to overcome confounding by indication and to prevent contamination between isolation strategies. Crossover of strategies at the hospital level was aimed at reducing between-hospital variability and, thus, increasing statistical efficiency.” 32

#### Examples of item 3a.2

*Example 2 (description of cross sectional design)*—“Hospitals were randomized 1:1 . . . to UCM [umbilical cord milking] or ECC [early cord clamping] in Period 1 (5 hospitals per treatment group from January 2019 to January 2020) until half of the required enrollment was reached (N=600 consented subjects); they were then crossed over to the other intervention during Period 2 (February 2020 to May 2021) for the remaining half of consented subjects (N=600). A 1- to 2-month washout period occurred after Period 1.”^28^

*Example 3 (description of closed cohort design; number of schools allocated to each sequence shown in* figure 2 *of Makris et al* 33 *)—*“The trial was conducted in six primary schools with two periods (40-days organic diet vs. 40-days of conventional diet) . . . A washout period was not required as it was intrinsically included in the two periods, since the first urine sample [for measurement of the primary outcome] of the second period was collected about 12 days after the beginning of the second period. Moreover, the pesticide half-lives are short (half-lives ranging 6.4-16.5 hours for pyrethroids and 5-33 hours for neonicotinoids), so no carryover effect was expected.”^33^

**Fig 3:**
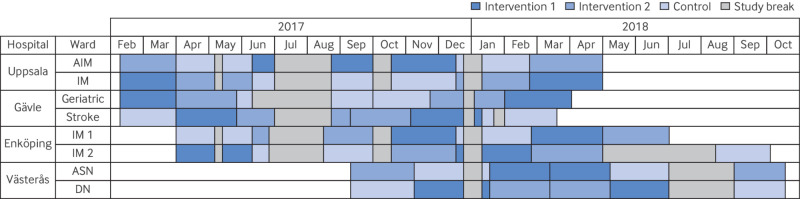
Diagram of the realised design from the MedBridge trial.^34^ AIM=acute internal medicine; ASN=acute stroke and neurology; DN=diabetes and nephrology; IM=internal medicine. Adapted from reference 28 with permission

*Example 4 (diagram of realised design from MedBridge trial* 34 *)*—see figure 3.

### Item 3b: Trial design

Important changes to planned methods after trial commencement (such as eligibility criteria), with reasons.

#### Explanation

Changes from the planned methods might need to be made after the trial has commenced. Important changes should be fully reported to enable readers to appropriately interpret the results. For example, changes in a CRXO design might arise from delays in recruitment of either clusters or participants within clusters, which could lead to unintended staggered commencement of the trial in different clusters, or differences in period lengths. Such changes have the potential to bias estimates of treatment effect, and therefore are important to describe.

#### Example of item 3b

*Example (deviation from planned dates)*—*“*The study had 1 major deviation. Originally, the initial phase was to run from November 2017 to February 2018, and the crossover phase from March to June 2018. However, because of logistics issues faced by all clinics with obtaining seasonal influenza vaccine supplies ahead of the midyear season, commencement of the crossover phase was delayed by 1 month. We retained the study design of two 4-month phases and ran the initial and crossover phases from November 2017 to February 2018 and from April to July 2018, respectively.”^35^

## Methods: Participants

### Item 4a: Participants

Eligibility criteria for clusters and participants.

#### Explanation

A comprehensive description of the eligibility criteria is required to help readers determine the population to whom the results of the trial were intended to be generalised. The criteria should be framed in such a way that a reader can recognise whether the trial includes typical or atypical clusters and participants.^36^ Inclusion and exclusion criteria need to be reported for both clusters and participants. In addition, in some cluster trials, there may be multiple levels of participants (eg, emergency department staff and patients attending the emergency departments), and eligibility criteria should be reported for each.

#### Example of item 4a

*FLUID trial*—“Inclusion criteria at hospital level: participating hospitals were required to have a level II or III ICU [intensive care unit] as these hospitals have the capability of admitting patients that are more severely ill and in turn may receive more fluid administration than hospitals with a level I ICU.

“Exclusion criteria at the hospital level: we excluded hospitals that had fewer than 6000 acute care admissions per year (<1500 admissions per study period).

“Inclusion criteria at patient level: adult and paediatric patients admitted to the participating hospitals for the first time in the previous 90 days (index admission) over the duration of each study period were included in FLUID (to avoid exposure and thus potential contamination with either crystalloid fluid in the prior 90 days).

“Exclusion criteria at patient level: neonates were excluded from FLUID since RL [Ringer’s lactate] is neither used nor recommended for use in this population. Patients who were readmitted to hospital during study period 1 or 2 were excluded to avoid contamination with previous FLUID exposure. Patients admitted during the run-in or run-out study periods were also excluded.”^37^

## Methods: Intervention

### Item 5: Intervention

The treatment conditions with sufficient details to allow replication, and whether they were delivered at the level of the cluster, the individual, or both.

#### Explanation

A complete description of the intervention conditions is necessary for their future implementation.^38^ The TIDieR (template for intervention description and replication) checklist provides an extension of the CONSORT statement (item 5)^3^ for describing interventions.^38^ Complete description also allows assessment of the potential for carryover effects (table 1). Unlike other cluster designs (parallel, stepped wedge), the CRXO design is not suitable for evaluating treatments where their effects persist, such as educational interventions targeted at changing clinicians’ practice.^30^

For cluster trials, reporting whether the treatments are delivered at the cluster level, the individual participant level, or both, is important. This information allows assessment of whether individuals can avoid treatments (which is important for determining the coverage of the treatment) and has implications for the identification of research participants and requirements for informed consent (items 10c and 26).

In CRXO trials, treatments are commonly delivered at the individual participant level (eg, daily bathing of critically ill children in paediatric intensive care units with chlorhexidine gluconate compared with standard bathing practices) because these treatments can be withdrawn.^8^ Treatments delivered at the level of the cluster can also be evaluated using the CRXO design, as long as the intervention can be withdrawn (eg, enhanced cleaning of hand contact surfaces within intensive care wards compared with standard cleaning, where the active treatment component of extra hand gel on wards can be removed).

#### Examples of item 5

*Example 1 (description of the treatment conditions; level of delivery to the individual)*—“In each group, the intervention was applied at the patient level. 

“For patients included during experimental periods, hospital pharmacists performed the medication reconciliation at discharge . . . Hospital pharmacists completed a short form documenting the reason for hospitalisation, home medication modifications, new medication and laboratory results necessary for community pharmacists to understand and/or accept the prescription . . . They also checked the discharge prescriptions (drug added and/or omitted, different dosage, route or duration of treatment) and, if needed, made an intervention on the physician’s prescription according to SFPC [French Society of Clinical Pharmacy] standards [standards provided in a figure] to change the prescription. Then they explained to the patient the drug initiated and the modifications to the home medication. They phoned the patient’s community pharmacist to explain the patient's inclusion in the study, the discharge time, and the modifications in treatment. They also sent the prescription sheet to the community pharmacist before patient discharge. The patient or care giver then visited the community pharmacist as usual.

“For the control group, patients received the usual care already implemented both at the hospital (classical drug dispensation by staff pharmacists) and by their community pharmacist (drug dispensation according to the prescription sheet written by the hospital physician in addition to the general practitioner’s sheet [if present]).” 39

*Example 2 (level of delivery to the cluster)*—“The study fluid interventions were implemented at the hospital level using a hospital policy or strategy, with the aim to answer our study question at the hospital level.” 37

## Methods: Outcomes

### Item 6a: Outcomes

Completely defined prespecified primary and secondary outcome measures, including how and when they were assessed (for specific guidance see CONSORT for outcomes).

#### Explanation

Outcomes should be completely defined, such that the resulting treatment effect estimates can be interpreted and used in other research (eg, systematic reviews). Here, we focus on issues particular to CRXO trials, and refer readers to the CONSORT 2010 statement and the CONSORT-Outcomes extension for further discussion of aspects common to all designs and examples.^3^ ^40^

In cluster randomised trials, outcomes in each period can be measured at the level of the individual or at the level of the cluster. The second option could reflect a true cluster level outcome or arise because only aggregated individual level data are available (eg, the number of hospital associated infections per period might be available only in summary form).^12^ The level of the outcome measure has implications for the method of analysis (item 12a).

The timing of outcome assessments should be reported, including whether the outcomes were measured at discrete points in time common to all participants (eg, via a survey at the end of each period), or at a time point specific to each participant (eg, number of adverse events per patient until the time of their hospital discharge). Reporting of the timing of follow-up facilitates assessment of whether participants had similar (or variable) duration of exposure to the treatment conditions (table 1), and whether there was the possibility of contamination of observations collected in the second (or later) periods. Possible contamination might arise in cross sectional designs where the treatment is delivered at the level of the cluster.

In CRXO trials, data might be sought from routinely collected sources (eg, registries, hospital medical records) or purposively collected sources (eg questionnaires, patient diaries). Reporting the methods for data collection is important for allowing readers to assess whether the methods are appropriate for measuring the intended outcomes and facilitates an assessment of the potential for bias arising from measurement of the outcomes.^41^

#### Examples of item 6a

*Example 1 (description of outcomes; primacy; outcome measured at level of individual; time point specific to each participant)*—“Primary outcome and timing: All the outcomes pertain to the individual participant. The primary outcome was admission to the NICU [neonatal intensive care unit] in the first 24 hours of life for predefined criteria as follows: respiratory distress (tachypnea, grunting, or retractions), bradycardia or tachycardia, hypotonia, lethargy or difficulty arousing, hypertonia or irritability, poor feeding or emesis, hypoglycemia, oxygen desaturations or cyanosis, need for oxygen, apnea, seizures or seizure-like activity, hyperbilirubinemia, and/or temperature instability.

*“*Secondary outcomes and timing: The predefined secondary safety and efficacy outcomes included therapeutic hypothermia, volume expanders, phototherapy, haemoglobin at 24 hours of life, and peak serum bilirubin.”^28^

*Example 2 (outcome measured at level of cluster; use of administrative data)*—“Primary feasibility outcome: Adherence to the fluid protocol: Adherence to the study fluid was measured not at the individual patient-level, but according to the aggregate use of the study fluid throughout the hospitals (all hospital wards, monitored units, and departments) using the hospital inventory system; monitoring fluid exposure or adherence according to individual patients was not feasible due to the sheer number of hospital admissions . . . 

“Data collection: All follow-up and collection of data for enrolled patients at the participating hospitals were captured through health administrative data that are housed at the Institute for Clinical Evaluative Sciences. There were no individual patient level data collected by research coordinators in the participating hospitals.” 37

*Example 3 (description of outcomes; primacy; outcome measured at level of individual (implicit); time point specific to each participant; data collection methods)*—“The primary outcome was symptomatic VTE [venous thromboembolism] within 90 days of surgery . . . 

“Data source and timing: Patients received a web link via email or text message to complete online data collection at 90 days postoperatively . . . Data collection forms were reviewed by registry staff and all patients who responded “yes” to having had a venous thromboembolism, a secondary operation within 90 days or 6 months, a major bleeding event within 90 days, or a joint-related readmission within 90 days had this outcome verified through written documentation from treating physicians. These results were verified by the trial outcome verification committee. For the primary outcome, the date and type of VTE (below- or above-knee deep venous thrombosis [DVT] or pulmonary embolism) and side of DVT (left or right) were recorded. Mortality data were collected through linkage between the registry and the National Death Index.” 42

## Methods: Sample size

### Item 7a: Sample size

How sample size was determined. Method of calculation and relevant parameters with sufficient detail so the calculation can be reproduced (table 4). Assumptions made about correlations between outcomes of participants from the same cluster.

#### Explanation

Despite improvements over time, most randomised trials remain insufficiently powered to detect the small effects that are generally observed.^43^ Reporting the sample size calculation in sufficient detail is important to ensure reproducibility and for allowing assessment of the plausibility of the assumptions, and identification of any scientific or ethical concerns arising.^19^

The method used to determine the sample size should be described, specifically noting how the calculation accounts for both the cluster randomisation and the multiple period aspects of the design (seen in examples elsewhere^21^ ^44^ ^45^). For CRXO trials, as in any cluster randomised trial, it is important to account for the intracluster correlation coefficient; however, in a CRXO trial, a single intracluster correlation coefficient might not be adequate because the strength of the intracluster correlation could depend on the separation in time of the observations. The recommended minimum items to report are given in table 4. The details to provide depend partly on the particulars of the design (eg, whether the design is cross sectional or cohort).

Justification for the choice of parameter values such as intracluster correlation coefficients should be provided (eg, based on estimates reported in a repository of correlations or extrapolation from known determinants of correlations such as whether the outcome is a process or clinical outcome).^46^ ^47^ Often sample size parameters (eg, intracluster correlation coefficients, cluster autocorrelation coefficient) are based on estimates with large uncertainty. Therefore, the protocol should include an indication of the sensitivity of the sample size, or power, to the assumed parameter values, and should be available with the trial report.^19^

In some CRXO trials, the sample size might be fixed by constraints on the number of clusters, participants, or periods. In this circumstance, the effect size that the trial was powered to detect should be reported. If no power calculation was performed, this should be reported. Retrospective power calculations based on the results of the trial are of little merit and are discouraged.^48^

#### Example of item 7a

*PEPTIC trial (some core items are described elsewhere in the publication: two periods; two sequences; 1:1 allocation ratio)*—“There is no established consensus regarding what constitutes a minimal clinically important difference in in-hospital mortality for critically ill adults. This . . . makes it challenging to establish what difference in mortality one should power an ICU study to detect . . . Accordingly, we sought to conduct the largest trial that we considered feasible in order to provide the most precise estimates of the likely mortality treatment effect possible with PPIs [proton pump inhibitors] vs. H2RBs [histamine-2 receptor blockers]. In the study protocol the sample size was calculated [reference to sample size method] to have 80% power at a 5% significance level to detect a 2.4% absolute reduction in in-hospital mortality from a baseline mortality of 15%, which corresponds to a relative risk reduction of 16%. This calculation assumed a mean cluster-period size of 310 patients with coefficient of variation of 0.50, a within-cluster-within-period correlation of 0.035, and a within-cluster-between-period correlation of 0.025, yielding a cluster autocorrelation of 0.71.” (Supplement 2 of PEPTIC trial.^9^)

### Item 7b: Interim analyses

When applicable, explanation of any interim analyses and stopping guidelines.

#### Explanation

Interim analyses of outcomes are undertaken to inform the decision of whether a trial should be stopped early if there is substantial evidence of harm, effectiveness, or futility. Authors should report the dates or times at which they were carried out, what triggered them, whether these were planned or ad hoc, and the statistical methods used (including any formal stopping rules). When stopping rules or guidelines are used, it is important to report whether these were planned before commencement of the trial and before assessment of any interim analysis results, or some time thereafter.

For CRXO trials, little methodological guidance exists on how to do an interim analysis of outcomes, and interim analyses are challenging owing to the complexity of this design. For example, whether commencement of clusters is concurrent or staggered will affect the type of interim analysis. If commencement of clusters is concurrent, in a two period design when the analysis is undertaken at the end of the first period, the design would reduce to a parallel cluster randomised trial, necessitating a different analysis from the planned final analysis. If commencement of clusters is staggered, some clusters might have completed both treatments, others only one, and some a mix of partially completed treatments. Any interim analyses should address these issues, and the properties of any stopping rules should be determined, with these details reported. As with any trial, the planned interim analyses (where a decision is to be made about continuation of the trial) should be allowed for in power calculations to control the overall type I error rate.^12^

Other important reasons for considering whether to stop a trial arising from the trial conduct itself include: the inability to recruit clusters or clusters unable to recruit participants as planned, and poor quality of the primary outcome. Reporting the date when the trial was stopped, along with the reasons for stopping, is recommended.

#### Example of item 7b

“An interim analysis was not initially planned, as both treatments are considered standard practice for venous thromboembolism prophylaxis in Australia and the trial is investigating an adverse event as the primary outcome. However, due to concerns of an increased adverse event rate (symptomatic thromboembolism and death) in one of the prophylaxis groups, a Data Safety Monitoring Board (DSMB) was convened 1 year into patient recruitment . . . The DSMB were advised by the Trial Management Committee (TMC) to conduct an interim analysis. In conjunction with the DSMB (prior to the interim analysis), the TMC applied the Haybittle-Peto stopping rule of a two sided significance of 0.001 for the primary outcome in population 1*.

“This stopping rule was chosen as it does not require adjustment of the significance threshold for the final analysis and allows further interim analyses using the same threshold (if required). After the first interim analysis (in September 2020), the DSMB recommended continuing the trial and performing a second interim analysis in November 2020 . . . 

“Interim analyses were conducted for thromboembolism and mortality within 90 days for population 1*. To account for unequal cluster sizes, incomplete crossover or clusters which had not yet crossed over, a composite analysis was designed. For clusters which had crossed over, including with partial completion of the second period, the cluster weighted estimator intended for the primary outcome was used. Harmonic mean weighting when there are unequal cluster sizes has been shown to improve precision and 95% confidence interval coverage compared with unweighted or inverse variance estimates [reference provided for methods]. Clusters which had not crossed over were analysed using the cluster period summaries, weighted by cluster size, in a parallel design approach, i.e., as if it were a cluster randomised trial without crossover. Estimates for the two approaches were combined using inverse variance weights to provide a final estimate. Confidence intervals were constructed using the Haybittle-Peto boundary of 0.001.” (Supplement 2 of Sidhu et al^42^; *Population 1=all registered patients undergoing primary total hip arthroplasty or total knee arthroplasty for a diagnosis of osteoarthritis.)

## Methods: Randomisation

### Item 8a: Allocation schedule generation

Method used to generate the random allocation schedule.

#### Explanation

In a CRXO trial, clusters are randomly allocated to a sequence of treatments, where the sequence defines the order in which each cluster will receive the treatments. For example, a two treatment, two period design might have two sequences: treatment A followed by treatment B (sequence 1), and treatment B followed by treatment A (sequence 2). The allocation schedule dictates the random allocation of clusters to either sequence 1 or 2. Note that our use of the term “sequence” differs from its use in the CONSORT statement^3^ and the cluster CONSORT extension,^5^ where for these parallel group designs, “sequence” refers to the randomly generated list of allocations to a single treatment (eg, treatment A or treatment B). In this CRXO statement, the term “schedule” is instead used to refer to this randomly generated list of allocations to sequences of treatments (eg, AB, or BA; table 5 presents an example).

**Table 5:** Distinguishing use of terms “sequence” and “schedule” in relation to random allocation of units in parallel group randomised trials and cluster randomised crossover (CRXO) trials

Randomisation unit (ie, participant or cluster)	Parallel group randomised trial (2 treatments, 1 period; allocation sequence)	CRXO trial (2 treatments, 2 periods; allocation schedule)
1	A	AB
2	B	BA
3	A	BA
4	A	AB
5	B	AB
6	B	BA

The set of candidate sequences and the method used to generate the random allocation of clusters to these sequences should be sufficiently described so that an assessment can be made as to its adequacy. Use of general terms such as “random,” “randomisation,” and “random allocation” are insufficient. Details of the method are required (eg, random numbers generated by computer).

#### Example of item 8a

“Each hospital unit is randomised to perform medical reconciliation or usual care for the first 14-day period and is crossed over to the other group for a second 14-day period.” (Figure 1 of Pourrat et al.^39^)

“The randomisation sequence was generated . . . using a computerised process.”^39^

### Item 8b: Randomisation method

Type of randomisation; details of any restricted randomisation, if used.

#### Explanation

In a CRXO trial, randomisation of clusters to the sequences of treatments is often done at a single point in time before the trial commences. In this circumstance, it might be possible to balance (restrict) the randomisation such that an equal number of clusters are allocated to each of the sequences of treatments, which can be achieved using a single block balanced across sequences for the entire study. For example, in a design of two periods, two treatments, and two sequences (AB or BA) with 20 clusters, a single block of size 20 balanced across the two sequences would allocate 10 clusters to AB and 10 to BA.

Stratification in CRXO trials might also be used for clusters with distinct characteristics (eg, different case mix, different hospital protocols for the delivery of care) that are related to the trial outcomes, or where commencement of clusters is staggered in time and there is potential for secular or seasonal trends. In stratified designs, balanced allocation of clusters to sequences are generated independently within each stratum. Other methods of restricted randomisation that might be used include covariate constrained allocation,^49^ minimisation,^50^ and matching.^51^

When reporting the type of randomisation, investigators should indicate whether clusters were allocated at one time, in batches or sequentially, and whether simple or restricted randomisation (eg, permuted blocks) was used. If permuted block randomisation was used, details should be provided on how the blocks were generated, the block size(s), and whether the block size was fixed or randomly varied. If stratification was used, researchers should provide details about the stratification variables and the cut-off values for categorisation.^3^ If another method of restricted randomisation was used, the method should be described, along with the variables used to restrict the randomisation.

#### Example of item 8b

*Example 1 (restricted randomisation using stratification; sequential allocation)*—“The ICUs [intensive care units] were consecutively randomized in a 1:1 ratio using computer-generated randomization with random block sizes of 2, 4, and 6 and stratified by number of ICU beds (≤10 or >10).”^52^

Consecutive randomisation was “as per the date of approval by the institutional review board” (supplement 1 of Rosa et al^52^).

*Example 2 (restricted randomisation using stratification; batch allocation)*—“Participating ICUs [intensive care units] were randomized to the order of treatments in a 1:1 ratio when ethics and regulatory approvals were in place at each study site. Randomization was performed using computer-generated random numbers and was stratified by region and time period with a minimum of 4 ICUs randomized at a time.”^9^

*Example 3 (simple randomisation)*—“Schools were randomized a priori to two groups that differed in the sequence of the treatments; organic diet followed by conventional diet (Group 1) or conventional diet followed by organic diet (Group 2).”^33^

“Schools were randomly assigned following simple randomization procedures of no restriction or matching.” (Supplement 5 of Makris et al.^33^)

### Item 9: Allocation concealment

Specification that allocation was based on clusters; description of any methods used to conceal the allocation from the clusters until after their recruitment.

#### Explanation

The benefits of randomisation are only realised when randomisation is properly implemented. In individually randomised trials, this process requires generation of a random allocation schedule, and concealment of upcoming assignments from the participants and from those responsible for recruiting the participants, until after recruitment (known as allocation concealment).^53^ Analogously, in cluster trials, where randomisation occurs at the level of the cluster, allocation concealment involves concealment of upcoming assignments from the clusters (ie, so-called gatekeepers or guardians) and from those responsible for recruiting the clusters. The randomisation process in CRXO trials might involve randomising clusters sequentially, in batches, or all at once.^54^ The strategies implemented to conceal the allocation will differ depending on the randomisation process, and should be completely described. As with other cluster randomised trials, allocation concealment is likely to be preserved when randomisation occurs at a single point in time.^54^

In cluster randomised trials, participants might be recruited (or identified) after the clusters have been allocated. This scenario can lead to recruitment or identification bias arising from selective recruitment (or identification) of participants across treatment conditions when those responsible for the recruitment are aware of the cluster’s allocation in a particular period. In this statement, the process for recruitment of individual level participants is considered separately (item 10b) to the randomisation process.

#### Example of item 9

*Example 1*—“Crossover and randomization took place at cluster (ward) level within each hospital . . . The computer-generated codes were held by the statistician to assure allocation concealment until the moment of randomization.” (Supplement 3 of Kempen et al.^34^)

*Example 2*—“Use of sequentially numbered sealed opaque envelopes enabled concealment of the allocation sequence at the hospital level until the last hospital was randomised.”^32^

## Methods: Implementation of randomisation

### Item 10: Implementation

Replaced by items 10a, 10b, and 10c (listed below).

#### Explanation

Consistent with the CONSORT extension for other clustered designs,^5^ ^12^ it is important that all steps in the implementation of the randomisation and recruitment processes for both clusters and individual level participants are described. Reporting of these processes allows readers to assess the potential for bias. Details of the allocation and enrolment process of the clusters are described in item 10a, with the corresponding detail provided for participants in item 10b. The consent process for participants is also dealt with under implementation of the randomisation methods (item 10c) because this component is integral to enrolment, with potential implications for bias (eg, when consent procedures differ across treatment conditions that might lead to different types of participants being recruited). Use of the timeline cluster tool, which depicts the time sequence of trial processes (including the randomisation process) and the blinding status of participants and trial staff at each stage, allows readers to easily assess threats to the internal validity of the trial.^55^

### Item 10a: Inclusion of clusters

Who generated the random allocation schedule, who enrolled clusters, and who assigned clusters to sequences of treatments in the schedule.

#### Explanation

Reporting who generated the random allocation schedule, who enrolled the clusters, and who assigned the clusters to the sequences of treatments is important for allowing assessment of the potential for bias.^54^ A guiding principle to achieve adequate allocation concealment involves separation between the person who generates the allocation schedule and those who enrol and assign the clusters to the sequences of treatments. This separation can be achieved by having someone (preferably a statistician) independent of the trial generate the allocation schedule. When this person is unaware of the identity of the cluster (eg, where they receive a non-identifying unique code for each cluster), they might also assign the clusters to the allocation schedule (ie, equivalently maintaining separation). Failure to separate the above processes risks potential subversion of the randomisation schedule and potential biased estimates of treatment effect.

#### Example of item 10a

*Example 1 (who generated the random allocation schedule, who enrolled clusters)*—“Potentially eligible health services were identified in consultation with Departments of Health and Aboriginal Community Controlled Health Organisations and enrolled by study coordinators (AT, BH, and SGB) . . . The trial statistician (HW) did the randomisation . . . Participating health services were told their group allocation once participation agreements were signed.”^56^

*Example 2 (who generated the random allocation schedule, who assigned clusters to the sequences of treatments)*—“To remove the potential for allocation bias, one statistician generated the allocation codes and another randomly permuted clinic names within strata. A third person matched the allocation codes with clinic names to reveal the allocations.” 57 [Note that the role of the third person should be specified.]

### Item 10b: Inclusion of participants

Mechanism by which individual participants were included in clusters for the purposes of the trial (such as complete enumeration or random sampling, continuous recruitment or ascertainment, or recruitment at a fixed point in time), including who recruited or identified participants.

#### Explanation

As with other cluster randomised designs,^5^ ^12^ there are different sources from which the individual participant data can be obtained (eg, directly from the patient or from review of their medical records), different sampling strategies (eg, complete enumeration or random sampling), and different timings of the data collection (eg, continuously or at fixed points in time). These mechanisms have differing risks of bias.

In some CRXO trials, data are not directly sought from participants, but instead obtained from routinely collected sources (such as hospital medical records). In these trials, participants are identified (ascertained) but might not be approached directly for recruitment into the trial. Alternatively, participants could be recruited into the trial and might need to provide data prospectively, for example, through questionnaires. Recruitment can occur continuously or at fixed points in time. Recruitment or identification might involve approaching or identifying all participants or a sample.

Reporting the mechanisms for including individual participants, covering who was involved in recruiting or identifying participants, and whether they were blinded to the cluster’s treatment allocation in a particular period, allows assessment of the likelihood of bias arising from selective recruitment or identification of participants.^54^ In trials where participants are recruited or identified before randomisation, there is no potential for recruitment or identification bias (because selection of participants cannot be influenced by the cluster’s treatment allocation, which is unknown before randomisation). However, in CRXO trials, the possibility of using this approach is limited to only a closed cohort design (where the same participants are included in all periods), which is a less common design.^8^ More commonly, participants are identified or recruited after randomisation. In this circumstance, recruitment or identification of participants by a person unaware of the allocation of the cluster can help mitigate recruitment or identification bias. Trials with complete enumeration might have less scope for selective recruitment or identification of participants, even when the recruiter is aware of the cluster’s assigned treatment; nevertheless, concealing the treatment assignment from the recruiter is always advisable.^58^

#### Example of item 10b

*Example 1 (continuous recruitment; recruiters unblinded to cluster’s treatment allocation)*—“All adult patients were eligible, except those who stayed in the hospital longer than 21 days, who did not return home, who were in a moribund state, or who were not able to understand the topic of the study or complete a questionnaire.”^39^

“Participants are recruited by unblinded hospital pharmacists.” (Figure 1 of Pourrat et al.^39^)

*Example 2 (random sampling)*—“Because of the variability in cluster size, the female participants were only randomly selected for enrolment by probability proportional to size sampling. For the male participants’ enrolment, all the eligible participants were enrolled except for in three clusters whose size allowed random selection.”^59^

### Item 10c: Consent

Whether consent was sought, from whom, when, and for what; whether this differed between treatment conditions. Justification for any waiver or modification of informed consent requirements.

#### Explanation

Recruitment and consent procedures in cluster randomised trials are more complex than in individually randomised trials.^60^ In individually randomised trials, informed consent is usually obtained from individual participants, and consent generally encompasses being randomised, receiving study interventions, and providing data for outcome assessment. In cluster randomised trials, however, clusters are randomised—often before individual participants can even be identified for the trial, while study interventions and data collection procedures can be administered to different types of participants. Thus, the randomisation units, the units exposed to the intervention, and the units on which outcomes are assessed might be different. For example, hospitals could be randomised, the intervention could be given to providers, while outcomes are assessed on patients. Reporting of explicit details about the recruitment and consent procedures in CRXO trials is essential to avoid confusion. A simple statement that “participants provided informed consent” is not adequate; instead, referring to specific participants (eg, providers, patients) and indicating what consent pertains to (eg, exposure to the intervention or data collection) aids clarity.^61^ While so-called gatekeepers or cluster guardians can have an important role in facilitating the recruitment of clusters into the trial,^62^ gatekeeper permission for trial participation should not be confused with consent from research participants.^14^

Clarity around the timing of any consent relative to the timing of randomisation and intervention delivery is also particularly important in cluster randomised trials, because consent after randomisation increases the risks of bias.^63^ In a minority of CRXO trials (those with closed cohort designs), it might be feasible to identify and recruit individual participants before cluster randomisation, but most CRXO trials have cross sectional designs in which participants are recruited prospectively.^8^ In CRXO trials and in those CRXO trials with multiple periods, investigators should describe consent procedures in adequate detail so that it is clear whether consent procedures differed over time and between treatment conditions in different periods because these events can lead to bias.^64^ Steps taken to mitigate the risks of bias should also be clearly described.^54^ ^58^ Making recruitment materials and consent forms available in supplementary materials can aid clarity around what information was conveyed to participants about the trial, while providing timeline cluster diagrams can aid clarity around the timing of consent.^55^

In some CRXO trials in which outcomes are available in routinely collected databases and study interventions pose only minimal risk to research participants, a research ethics committee could give permission for a CRXO trial to proceed without the explicit informed consent of research participants. When an ethics committee has approved the study with a waiver of informed consent, this approval should be clearly stated together with the justification for the waiver according to the regulatory requirements of the country or countries where the CRXO trial took place.

#### Example of item 10c

*Example 1 (consent procedures differ between treatment conditions for patients; timing of consent)*—“6a. Participant recruitment in the medication reconciliation group . . . [Participants] receive complete information and provide oral consent for intervention and for data collection . . . 6b. Participant recruitment in the usual care group . . . [Participants] receive partial information because they are not aware of the existence of the medication reconciliation group and provide oral consent for data collection.” (Figure 1 of Pourrat et al.^39^)

*Timeline cluster diagram*—See supplementary materials 3, figure S1.^39^

*Example 2 (waiver of consent to exposure to intervention)*—“We conducted a cluster randomized multiple crossover trial to evaluate a real-time best practice alert embedded into electronic health records . . . Institutional review board approval was obtained before patient enrolment . . . Patient and provider consent was waived because the alert supplemented the current standard of care, there was no requirement to respond, and the alerts were unlikely to be harmful and might have proven beneficial.”^65^

## Methods: Blinding

### Item 11a: Blinding

Who was blinded after assignment to sequences (eg, cluster level participants, individual level participants, those assessing outcomes) and how.

#### Explanation

Clear reporting of whether those involved in the delivery of the treatments, receipt of the treatments, outcome collection and adjudication, and data analysis, were blinded to the treatment allocation is important for assessing the likelihood of bias. In some CRXO trials, participants are unaware of their treatment allocation because they do not know they are in the trial (eg, when they are not directly recruited), or they might know they are in a study, but not a trial.^54^ The level of awareness that participants have about their participation in a trial can affect the likelihood of bias. Therefore, explicit reporting of participants’ knowledge of their participation in the trial is important.

Performance bias could arise when healthcare providers or individual level participants are aware of the treatment allocation, and this knowledge leads to deviations from the intended treatment (eg, use of co-interventions, increased or decreased compliance with the treatment).^41^ Similarly, detection bias might arise when the outcome assessor or adjudicator is aware of the treatment allocation, which leads to differential assessment of the outcome. The outcome assessors can be cluster participants (eg, patients or healthcare providers that self-report outcomes), healthcare providers responsible for outcome assessment (eg, a clinical examination), trial personnel, or people who are independent of delivery of the treatment. Many trials will have different types of outcome assessors; therefore, clearly reporting which outcomes were assessed by whom, and whether they were blind to the treatment allocation is necessary.

Parallel and stepped wedge cluster randomised trials commonly evaluate treatments delivered to cluster level participants (eg, educational interventions delivered to healthcare providers to improve practice),^66^ ^67^ and in this circumstance, blinding of these participants would generally be difficult.^54^ However, in CRXO trials, treatments delivered at the individual participant level are more likely (because these treatments can be withdrawn—see item 5), and could provide more opportunity to use blinding of cluster level and individual level participants.^8^ The consequence of a lack of blinding of those people delivering the treatments (eg, clinicians) on performance bias might be of particular concern in CRXO trials. If changes in outcomes for individual level participants are observed in the first period by those delivering the treatment, this could lead to the continued use of the treatment condition in subsequent periods (a type of contamination).

#### Example of item 11a

*Example 1*—“Participants and ward staff were not blinded to treatment allocation.”^34^

“All primary and secondary outcome data collection and assessments were blinded to treatment allocation . . . Statisticians were blinded to treatment allocation until database closure.” (Supplement 3 of Kempen et al.^34^)

*Example 2*—“Blinding of clinicians and research staff will remain throughout the entirety of the trial . . . Trial participants, care providers, outcome assessors, and data analysts will be blinded to the assignment of intervention. The only research personnel who will be unblinded are the statistician performing the randomization, the PM [project manager], and the research pharmacists in charge of supplying locking kits used during the study . . . Data analysis will occur outside of the unit with the type of intervention received by each patient concealed until the completion of analysis.”^68^

## Methods: Statistical methods

### Item 12a: Statistical methods

Target estimand for each primary and secondary outcome including whether it pertains to the cluster level or individual level; statistical methods for their estimation, including how period effects, clustering, and repeated measures were taken into account. Any assessment of carryover effects should be reported.

#### Explanation

Primary reasons for reporting the statistical methods are to allow for reproduction of the results and for readers to assess whether the methods are appropriate for the design, and the research question. The guiding principle should be to provide enough detail such that a knowledgeable individual with access to the original data could verify the reported results.^69^ References to the chosen statistical methodology should be provided, along with details of the statistical software packages (and versions) used to implement the methods, and ideally the statistical code (items 24 and 27). The link between which statistical method(s) apply to which outcomes, should be made clear. Prespecified analyses should be distinguished from unplanned analyses. This distinction is important because prespecification limits bias arising from selective reporting of favourable results from fitting multiple analyses (commonly known as p hacking), and so prespecified analyses are considered more trustworthy.^70^ ^71^

Reporting the precise description of the treatment effect to be estimated for each outcome (ie, the estimand) allows readers to understand the specific questions asked,^23^ which should align with the study objectives (item 2b). The attributes for completely specifying an estimand are outlined in box 2. In addition to reporting the standard attributes for individually randomised trials (ie, attributes 1-5), investigators of cluster trials should report whether a participant average treatment effect or cluster average treatment effect was targeted (attribute 6).^74^ Attribute 6 will depend on the target of interest; that is, whether interest lies in how effective the intervention is on average for a population of participants (participant average treatment effect) or for a population of clusters (cluster-average treatment effect). Researchers should also specify whether interest lies in the marginal or cluster specific treatment effect.^75^

Box 2 **Estimands in cluster randomised trials: concepts and explanations**

**Estimand**

Randomised trials are used to answer questions about how well an intervention works. These questions might be about the effect of the treatment if all participants adhered, the effect in those patients who would adhere, or the effect of assignment to treatment irrespective of whether they adhered or not. This shows that there is not one single type of question of interest, and different trials might try to answer different types of questions. The exact question being asked is described by the estimand, which provides a clear description of what the treatment effect being estimated represents. It precisely describes what the numerical result (the estimate) will mean (eg, whether a relative risk of 0.80 implies a 20% relative reduction regardless of whether participants adhered, or only if all patients were to adhere).^72^

For cluster randomised trials, estimands are comprised several attributes: (1) population, (2) treatment conditions, (3) endpoint, (4) summary measure (eg, marginal risk ratio), (5) strategies used to handle intercurrent events (such as non-adherence at the patient or cluster level), and (6) whether a participant average treatment effect or cluster average treatment effect is to be used.

Certain estimand attributes link to other items in the CONSORT extension checklist for cluster randomised crossover trials; for example, the estimand population links to item 4a (the trial population) and item 12a (which includes the analysis population), and the estimand’s treatment conditions link to item 5 (intervention). However, the estimand attribute differs to these other items in that it pertains to the trial’s treatment effect (eg, which participants the treatment effect applies to; which treatments are being compared for the treatment effect) rather than the specific study methods (eg, which participants were eligible to be enrolled, which participants were included in the analysis, which treatments clusters are randomised to).

**Estimator**

The method of analysis used to calculate the estimated treatment effect.

**Estimate**

The numerical value computed by the estimator using the trial outcome data (eg, in a cluster crossover trial reporting an odds ratio of 1.51, the value 1.51 represents the estimate).

**Example of a participant average estimand (adapted from the PEPTIC CRXO trial^9^)**

The marginal, participant average risk ratio (summary measure) for in-hospital, all cause mortality up to 90 days from the index admission to intensive care unit (endpoint) in patients aged 18 years or older requiring invasive mechanical ventilation within 24 hours of admission without upper gastrointestinal bleeding (population), between a strategy of proton pump inhibitor versus histamine-2 receptor blocker given until death, discharge, or development of a clinically important upper gastrointestinal bleeding event, or until no longer indicated (treatment conditions), regardless of non-receipt of treatment, discontinuation, or receipt of alternate treatments (ie, treatment policy strategy for handling intercurrent events) (intercurrent events).

**Example of a cluster average estimand (adapted from the BETR CRXO trial^73^)**

The marginal, cluster average difference in infection rates (summary measure) for incident cases of *Clostridium difficile* infection during hospital admission (exposure period) (endpoint) among patients placed in a previously contaminated hospital room (population), between standard terminal disinfection (bleach) and enhanced terminal disinfection (beach and ultraviolet light) (treatment conditions), regardless of whether there was documented use of the ultraviolet device (intercurrent event).

For cluster randomised trials, analyses can be undertaken at the individual or cluster level, with the second scenario done by aggregating observations within each cluster (or cluster period) into one summary measure. The approach taken in the trial’s analysis should be reported. Note that the level of analysis is distinct from the level of targeted inference.^74^ Recent literature concerning parallel cluster trials has indicated that participant average treatment effects and cluster average treatment effects might differ if there is informative cluster size, namely when cluster size is predictive of participant outcomes or of the magnitude of treatment effect (ie, treatment effect heterogeneity). In such cases, the different levels of analysis might be targeting different estimands.^74^ Although further work is needed to assess the impact of informative cluster size in CRXO trials, it should be reported whether informative cluster size has been considered or dealt with in the analysis.

It is well recognised that the statistical analysis of cluster trials must account for the correlation of participant observations within clusters (intracluster correlation). In CRXO trials, the strength of this correlation might depend on the separation in time between the observations,^17^ ^76^ along with (where applicable) correlation in repeated measurements from the same participants. Analyses that account for these correlations must be used: analyses that do not allow for these aspects typically yield incorrect estimates of standard errors of treatment effects (and therefore confidence intervals with coverage that is less or greater than the nominal level (eg, 95%)),^10^ and in some circumstances, might bias treatment effects (eg, certain generalised linear mixed models where the random effects are mis-specified).^77^ Therefore, reporting the estimation methods and assumptions made about the intracluster correlation of observations at different periods or times (eg, constant correlation, continuous-time correlation decay, exponential decay) is recommended.^13^

In CRXO trials where commencement of the trial is staggered across clusters or where period lengths differ across clusters, adjustment for secular or seasonal trends in the analysis should be considered, and description of the modelling approach should be provided (eg, including calendar time periods as a fixed effect).

Any tests for carryover effects, or adjustment for carryover, should be reported. The two stage analysis procedure, which involves first testing for carryover effects and determining the subsequent analysis approach based on the result of the test, has been shown to adversely affect estimation of the treatment effect and its statistical properties.^78^ For these reasons, testing for carryover is not recommended, but if undertaken, or an analysis of only the first period data are presented as a result of the two stage strategy, the analysis strategy needs to be reported. Similarly, when an attempt is made to adjust for carryover (eg, in designs with more than two periods), the model used and its assumptions should be described explicitly.^79^

Given that CRXO trials typically include a small number of clusters,^8^ small sample standard error or degrees of freedom adjustments might need be applied to maintain the nominal type I error rate (eg, 5%) and confidence interval coverage.^80^ Any adjustments applied should be reported. Similarly, researchers should report any adjustments for prognostic factors at the participant level to mitigate any imbalance in participant characteristics between periods within a cluster and note if they were prespecified or not.

Any methods for handling of missing data at the individual and cluster level should be described.^81^ ^82^ For trials with deviations from the allocated sequences or in the planned crossover timings, details should be provided about how this was dealt with in the analysis. The handling of these two components—missing data and treatment of deviations from allocated sequences—defines whether an analysis follows the intention-to-treat principle.^3^ ^83^ However, challenges in applying the intention-to-treat principle in the analysis of parallel cluster randomised trials have been recognised.^63^ These issues are further exacerbated in multiple period cluster designs where different definitions of adhering to the allocated sequence might be adopted (eg, according to the planned dates of crossover, or to the order of the allocation sequence).^12^ For these reasons, explicitly reporting how deviations are handled and methods for missing data is preferable to using terms such as “intention to treat.”

For binary outcomes, reporting both absolute and relative effect sizes is recommended (item 17b). When a model based approach has been adopted, the method used to calculate the risk difference should be reported. Several methods exist, such as regression based approaches that directly yield estimates of risk differences (eg, fitting a binomial model with an identity link), or indirect approaches, such as marginal standardisation.^84^

#### Example of item 12a

*Example 1 (individual level analysis; allowance for clustering, period and secular trends; method for calculating absolute effect)*—“The analysis of the primary outcome used individual patient-level data and generalized estimating equations with a logarithmic link function, an exchangeable working correlation matrix, and robust standard errors using the ICU [intensive care unit] as the clustering unit. Because randomization was performed in batches of ICUs, covariate adjustment for randomization batch, the order of administration of the treatments, and batch × order interaction was performed to allow for separate order and secular time effects occurring in each of the randomization batches . . . Treatment comparisons are presented as risk ratios (RRs) and 95% CIs [confidence intervals] from the generalized estimating equation analysis, and as absolute risk differences and 95% CIs obtained by marginalizing (ie, standardizing) of the RR model [reference provided]. The approach to the analyses of the secondary end points appears in the [reference to supplement] . . . All statistical analyses were completed using Stata version 15 (StataCorp).”^9^

*Example 2 (handling of missing data)*—“The primary analysis will use all available data with no imputation. In addition, if the mRS [modified Rankin scale] at 90 days (the primary endpoint) or NIHSS [National Institute of Health Stroke Scale] at 7 days are missing for more than 10% of patients, missing data will be imputed using a fully conditional specification (FCS). The imputation model will include mRS at 90 days, the NIHSS and mRS scores at 7 days (or hospital discharge, if sooner), a variable indicating the cluster, a variable indicating the period, a variable indicating the intervention, and all baseline variables that are listed in Section 2.6 [sic] . . . The mRS at 90 days and NIHSS at 7 days will be imputed using an ordinal logistic model. Other variables will be imputed using either linear regression (for continuous/ordinal variables) or a discriminant function method (for nominal variables). Ten sets of imputed data will be created and analysed using the model described in Section . . . Estimates of the treatment effect (β in Model 1) and its standard-errors will be combined to obtain a pooled common OR [odds ratio] and 95% CI.” (Statistical analysis plan of reference 19.^26^)

*Example 3 (cluster level analysis; allowance for period and prognostic factor)*—“As primary analysis, the difference in risk of PJI [periprosthetic joint infection] after the intervention treatment compared with the control treatment will be estimated using linear regression for aggregated cluster-period data, with the proportion of events within a cluster-period as the dependent variable and treatment, cluster, period, and proportion of females within the cluster-period as independent variables. An estimated difference in risk with CIs [confidence intervals] and a 2-sided p-value will be presented.”^85^

### Item 12b: Additional statistical methods

Methods for additional analyses, such as subgroup analyses, sensitivity analyses, and adjusted analyses.

#### Explanation

CRXO trials, like other designs, will commonly investigate subgroup differences and might perform adjusted analyses.^3^ Sensitivity analyses might also be undertaken to examine whether the results are robust to the assumptions of the analysis. These analyses deal with the same question (ie, estimand) but make different (plausible) assumptions that could lead to different results,^86^ for example, sensitivity of the treatment effect and its standard error to the choice of intracluster correlation structure. Details of such sensitivity analyses should be reported.

#### Example of item 12b

“A post-hoc series of analyses were undertaken to gauge the sensitivity of the point estimate and confidence interval from the primary analysis of in-hospital mortality to 90 days to alternative analysis models and small-sample corrections to the robust/sandwich variance estimator, and to the use of degrees of freedom from the t-distribution.” (Supplement 2 of PEPTIC trial.^9^)

## Results: Participant flow (diagram is strongly recommended)

### Item 13a: Participant flow

The numbers of clusters that were assessed for eligibility and were randomly assigned to each sequence. For each sequence period (ie, each cell) or treatment condition, the following should be reported:

- numbers of clusters that received intended treatments and were analysed for the primary outcome; and
- numbers of participants who were assessed for eligibility, received intended treatments, and were analysed for the primary outcome.

### Item 13b: Participant attrition

For each sequence period (ie, each cell) or treatment condition, losses and exclusions for both clusters and participants with reasons.

#### Explanation for items 13a and 13b

Providing information on the flow of clusters and participants at each stage of a randomised trial allows assessment of generalisability and the likelihood of selection and attrition bias. A flow diagram provides a succinct visual depiction of this information, which is particularly valuable for more complex trials.^3^

For CRXO trials, the flow diagram can be structured in several ways (eg, by each sequence period or treatment condition). Different structures will be more or less suitable depending on the CRXO trial’s specific design features, such as the number of sequences and periods, and whether the same people, different people, or a mixture are assessed in different periods. In some circumstances, presenting multiple flow diagrams using different structures might aid interpretation.

For a standard CRXO trial, the flow diagram is ideally structured by sequence period, where for each sequence the cluster and participant level numbers are reported for each period. Such a structure allows assessment of differential recruitment or identification and attrition across treatments and across periods. Structuring the flow diagram by only treatment condition could be considered for more complex cross sectional designs (eg, more than two periods, treatments, or sequences) where a flow diagram structured by sequence period might be unwieldy. However, structuring by only treatment condition has the disadvantage of not allowing assessment of differential inclusion and attrition across treatments and periods. Furthermore, this structure is problematic for cohort designs, because it is likely to overrepresent the numbers of individual level participants as they contribute measurements under multiple treatment conditions.

#### Examples of items 13a and 13b

*Example 1 (flow diagram structured by sequence-period cross-sectional design)*—See supplementary materials 3, figure S2.

*Example 2 (flow diagram structured by treatment condition)*—See supplementary materials 3, figure S3.

## Results: Recruitment

### Item 14a: Recruitment

Dates of treatment periods and washout periods.

#### Explanation

Providing the dates of the treatment periods and washout periods is important for providing historical context for the trial (in particular, the treatments evaluated)^3^ and for assessing the potential for biases arising from carryover effects or secular trends. Providing the dates of the washout periods allows assessment of whether the implemented length (which might differ from that planned (item 3a)) was sufficient to mitigate any carryover effects. Providing dates of treatment periods across clusters allows assessment of potential confounding bias arising from secular trends when there is staggered commencement, or different period lengths, across the clusters. In a simple design that is implemented as planned (eg, where the treatment and washout periods commence at the same set of dates for all clusters), reporting the dates for all clusters in the text is likely sufficient. For more complex designs that have staggered commencement (whether by plan or as implemented (items 3a and 3b)), displaying the dates in graphical form for each cluster is recommended.

#### Example of item 14a

**Fig 4:**
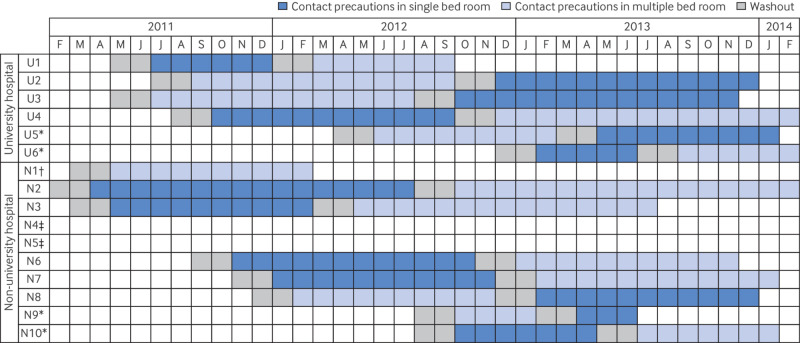
Example of dates of treatment periods and washout periods for each cluster in a cluster randomised crossover trial. Adapted from reference 26 with permission from Elsevier. *Two university hospitals and two non-university hospitals were additionally recruited in 2012. †One non-university hospital withdrew at time of crossover to second study period. ‡Two non-university hospitals, randomised to sequence multiple bed room/single bed room, withdrew after randomisation

*Example (dates of treatment and washout periods for all clusters depicted in graphical form)*—“13 hospitals completed both study periods and assessed 1652 index patients for eligibility from April 24, 2011, to Feb 27, 2014” (fig 4).^32^

## Results: Baseline data

### Item 15: Baseline data

A table showing baseline cluster level characteristics by sequence, and individual level characteristics for each sequence period (ie, each cell) or treatment condition.

#### Explanation

Reporting of baseline characteristics provides information on the characteristics of the clusters and individual level participants that were actually recruited to the trial, which might differ from the eligibility criteria (eg, if only urban emergency departments agree to participate in a trial that was open to all emergency departments). This information is necessary for generalising the findings (item 21). In addition, reporting of baseline characteristics allows assessment of the balance of these characteristics across the randomised sequences, and in trials with post-randomisation recruitment of participants, can allow assessment of potential recruitment or identification bias and potential changes to the case mix of participants over time.

In trials with multiple periods, the concept of “baseline” is more complex because of the longitudinal nature of the design.^12^ Here, the CONSORT stepped wedge terminology is adopted where a baseline characteristic is considered one that is measured before exposure to the treatment condition or one that is not expected to be influenced by the treatment (eg, age). Therefore, these baseline characteristics might apply differently to cluster and individual level characteristics. Baseline cluster characteristics can usually be measured before randomisation (and are often time invariant), while baseline individual characteristics might be assessed when the participant is recruited or identified (which could be after randomisation), but before they have been exposed to a treatment condition.

The baseline characteristics table can be structured in several ways, with different structures being more or less suitable depending on the CRXO trial’s specific design features (eg, number of sequences and periods; whether the same people, different people, or a mixture, are included in different periods), and the type of characteristics to be summarised (ie, cluster or individual level). Different structures have advantages and disadvantages, so presenting multiple might aid in interpretation. Regardless of the structure of the table, the statistics reported should be descriptive (eg, means and standard deviations), and not inferential (eg, standard errors).^3^

For a standard CRXO cross sectional trial, tabulating baseline characteristics at the individual level for each sequence period allows assessment of the balance of these characteristics across the randomised sequences, the potential for recruitment or identification bias, and any systematic change in characteristics across the periods.^13^ For time invariant characteristics at the cluster level, tabulating by sequence will be sufficient for assessing the balance of these characteristics across the randomised sequences. Tabulation by treatment condition could be considered for more complex cross sectional designs (eg, more than two periods, treatments, or sequences), where presentation by sequence period might be unwieldy. Although this structure does not reflect the design, it still might be useful for identifying selective recruitment or identification of participants.

#### Example of item 15

*Example 1 (baseline table by sequence period)*—See supplementary materials 3, table S2.

*Example 2 (baseline table by treatment condition)*—See supplementary materials 3, table S3.

## Results: Numbers analysed

### Item 16: Numbers analysed

The number of observations and clusters included in each analysis for each treatment condition and whether the analysis was according to the allocated schedule.

#### Explanation

The number of clusters and observations by treatment condition should be reported for analyses of all outcomes. For the primary outcome(s), this information will be attainable from the flow diagram of clusters and participants through the trial (item 13a). For analyses of secondary outcomes, reference can be made to the flow diagram when the same number of clusters and participants are included. For secondary outcomes where not all clusters or participants contribute to each analysis, in addition to providing the number of clusters and observations by treatment condition, it can be useful to also provide these numbers by sequence (eg, table 6 and table 7), or ideally, as a flowchart in accordance with the primary outcome (ie, structured by sequence period, where for each sequence the cluster and participant numbers are reported for each period).

**Table 6:** Possible table structure 1 for reporting the number of clusters and observations included in each analysis per outcome by sequence for a standard cluster randomised crossover (CRXO) trial

Outcome	No of clusters (No of observations)			
	AB clusters	BA clusters	A only clusters	B only clusters
X	8 ( . . . )	8 ( . . . )	2 ( . . . )	2 ( . . . )
Y				
. . .				

**Table 7:** Possible table structure 2 for reporting the number of clusters and observations included in each analysis per outcome by sequence for a standard cluster randomised crossover (CRXO) trial

Outcome	No of clusters (No of observations)						
	Sequence AB				Sequence BA		
	AB clusters	A only clusters	B only clusters		BA clusters	B only clusters	A only clusters
X	8 ( . . . )	2 ( . . . )	0 ( . . . )		8 ( . . . )	2 ( . . . )	0 ( . . . )
Y							
. . .							

This table provides more complete information than table 6 because it separates the number of clusters that contribute to only one period by sequence. Note that when there is no withdrawal of clusters, the columns with “A only” or “B only” headings are not required.

The type of summary information might differ depending on whether the same participants, different participants, or a mixture are assessed in different periods. For example, where the same participants contribute repeated measurements across the periods, they will have been exposed to both treatment conditions; therefore, the total number of observations can be presented by treatment condition, or as the number of participants in the study with the average number of observations per participant under each treatment condition.^12^

Providing information on whether the analysis was according to the allocated schedule, or if not, how deviations were dealt with in the analysis, is recommended. A detailed consideration of this issue is given for item 12a (statistical methods).

#### Examples of item 16

*Example 1 (numbers by treatment condition)*—“Of 38 assessed ICUs [intensive care units], eight fulfilled all eligibility criteria . . . 10 980 patients were admitted during the study period: 2204 during baseline, 4069 during cycling [treatment condition 1], and 4707 during mixing [treatment condition 2]. 1598 (18.2%) patients were present during the monthly point-prevalence surveys (745 during cycling and 853 during mixing), and were therefore included in the main analysis.”^87^

*Example 2 (table with the number of clusters and participants reported for each period within each sequence; noted analysis based on intention-to-treat)*—See supplementary materials 3, table S4.

## Results: Outcomes and estimation

### Item 17a: Outcomes and estimation

For each primary and secondary outcome, summary statistics by sequence period (ie, each cell) or treatment condition; the estimated effect size and its precision (eg, 95% confidence interval); and any within-cluster correlations or variance components estimated in the analysis.

#### Explanation

For each primary and secondary outcome, reporting summary statistics provides necessary information for assessing the magnitude or prevalence of the outcome in the trial participants, and the potential for any ceiling or floor effects. For a standard CRXO trial, presenting summary statistics by sequence period can allow assessment of the likelihood of any period effects, and allows readers to judge the appropriateness of the analysis. Presentation of summary statistics by treatment condition might be necessary for more complex designs (eg, more than two periods, treatments, or sequences), where presentation by sequence period might be unwieldy. Presentation of summary statistics by treatment condition is a natural approach; however, a simple comparison of summary statistics by treatment could lead to a biased estimate of treatment effect when period effects exist.

Estimated treatment effects with confidence intervals should be reported. P values might be provided, but these values should be in addition to effect estimates and confidence intervals, and should be reported as exact values (ie, not based on a significance threshold, for example, P=0.032 not P<0.05). The completeness of reporting of outcomes has been shown to be associated with statistical significance, where statistically significant outcomes are more often completely reported than non-significant outcomes, thus distorting the evidence base about the effects of treatments.^88^

Reporting of estimates of correlations arising from the CRXO design is critical for planning future trials.^5^ ^12^ For a CRXO trial with two periods, this report will include estimates of the within-period intracluster correlation coefficient and the between-period intracluster correlation coefficient (or cluster autocorrelation coefficient). With more than two periods, the parameter estimates of the assumed within-cluster correlation structure over time should be reported. Given the number of possible correlations, explicit reporting of the type of correlation alongside the estimate is necessary. Variance components can be reported as an alternative to intracluster correlation coefficients, particularly for non-continuous outcomes.^89^ When reporting intracluster correlation coefficients for binary outcomes, indication of the scale (eg, proportions or logistic) is necessary to know whether the intracluster correlation coefficient can be used in the variance inflation formula.^90^

#### Example of item 17a

*Example 1 (table with summary statistics (percentages) reported for each period within each sequence; estimated effect sizes (with 95% confidence intervals))*—See supplementary materials 3, table S4.

*Example 2 (table with within-period and between-period intracluster correlation coefficients)*—See supplementary materials 3, table S5.

*Example 3 (summary statistics by treatment condition)*—“The number of patients with at least one drug-related problem in the intervention and control groups was 236 (44.0%) and 280 (50.6%) respectively (OR [odds ratio] 0.77, 95% CI [confidence interval] 0.61–0.98). The intervention reduced the frequency of prescription and/or dispensing errors, patient errors and treatment gaps (OR 0.52, 95% CI 0.29–0.93; 0.84, 0.66–1.07; and 0.65, 0.43–0.99, respectively; [supplementary materials 3, table S4]). Within-period and between-period intra-cluster correlation coefficients are reported in [supplementary materials 3, table S5].”^39^

### Item 17b: Binary outcomes

For binary outcomes, presentation of both absolute and relative effect sizes is recommended.

#### Explanation

Reporting of both absolute effect sizes (eg, risk difference) and relative effect sizes (eg, risk ratio, odds ratio) for each binary outcome is recommended to allow readers to fully interpret the impact of the intervention. Relative effect measures are generally more stable across different levels of baseline risk,^91^ and therefore have the advantage of being more generalisable to populations or settings where the baseline risk might differ. However, presentation of only relative effects can lead to overstatement and overinterpretation of the intervention effect when the outcome is rare.^92^ For this reason, reporting of absolute effect measures is also recommended.^3^

#### Example of item 17b

*Example 1 (risk ratio and risk difference)*—“A total of 2459 of 13 415 patients (18.3%) in the proton pump inhibitor group died at the hospital by day 90 and 2333 of 13 356 patients (17.5%) in the histamine-2 receptor blocker group died at the hospital by day 90 (RR [risk ratio], 1.05 [95% CI [confidence interval], 1.00 to 1.10]; absolute risk difference, 0.93 percentage points [95% CI, −0.01 to 1.88 percentage points]; P=.054).”^9^

*Example 2 (table with odds ratios and risk differences presented for each binary outcome)*—See supplementary materials 3, table S4.^39^

## Results: Ancillary analyses

### Item 18: Ancillary analyses

Results of any other analyses performed, including subgroup analyses, sensitivity analyses, and adjusted analyses, distinguishing prespecified from exploratory.

#### Explanation

Readers are referred to the CONSORT statement for discussion of subgroup analyses and adjusted analyses.^3^ Reporting results from any sensitivity analyses undertaken is important for allowing readers to assess how departures from assumptions of the primary analyses might affect the interpretation of the results. Prespecified sensitivity analyses should be distinguished from those that were not planned.^23^ At a minimum, reporting the estimated effect size and its precision from each sensitivity analysis is recommended; however, other relevant parameter estimates might be reported depending on the type of sensitivity analysis (eg, reporting estimates of correlations if a different intracluster correlation structure is assumed). Sensitivity analyses might be best presented in a table that includes the results from the primary analyses, sensitivity analyses, and details of the assumptions that underlie the primary and sensitivity analyses.

#### Example of item 18

“Post-hoc sensitivity analyses for the primary outcome variable [Supplementary materials 3, table S6] provides the point estimate (RR [risk ratio]) and 95% CI [confidence interval] for a range of different analysis models for the primary endpoint, with small sample adjustments to the robust/sandwich variance estimator and the use of t-distribution with 32 degrees of freedom. Overall, there was no variation of the point estimate (RR) across approaches, and minimal variation in the 95% CI endpoints (maximum difference of 0.01 from the primary analysis result).” (Supplement 2 of PEPTIC trial.^9^)

## Results: Harms

### Item 19: Harms

Important harms or unintended effects in each treatment condition (for specific guidance, see CONSORT Harms 2022 statement).

#### Explanation

Readers are referred to the CONSORT 2010 statement and the CONSORT Harms 2022 statement for examples and explanation.^3^ ^7^ However, for open or closed cohort CRXO designs where participants are followed over time, particular care is required in interpreting adverse events data because of the risk of carryover effects. The effects of any adverse events experienced in the first period could persist into the second (or later) periods, or the effects of the treatment condition received in the first period might persist into later periods. Therefore, adverse events experienced in later periods cannot necessarily be attributed to the treatment condition the participant is receiving at the time they experience the event.

## Discussion

### Item 20: Limitations

Trial limitations, in relation to sources of potential bias, imprecision, and if relevant, multiplicity of analyses. Consider potential carryover effects.

#### Explanation

Reporting of any limitations is important because this allows readers to assess the potential for bias in the results. A key limitation of a CRXO design is the potential for carryover whereby the effects of the treatment in one period persist into a subsequent period, thus potentially biasing estimates of the treatment effects. Other possible CRXO design limitations are outlined in item 3a and should be reflected on when they apply.

Possible limitations common to all randomised trial designs include imprecise estimates of treatment effect (eg, for secondary outcomes) and the risk of spurious statistically significant findings arising from undertaking multiple statistical tests (eg, for different outcomes, subgroup analyses). Interpretation and reporting of the results cognisant of these limitations is recommended. Readers should refer to the CONSORT 2010 statement for further discussion of imprecision and multiplicity of results.^3^

#### Example of item 20

“Carryover effects are often plausible in individual-patient crossover trials where patients receive both study treatments. However, such carryover effects are less plausible in our cluster randomized crossover study because (i) different patients were present in the different observation periods and were not allocated to both treatments, and (ii) retention of ICU [intensive care unit]-level practices from the previous period on the basis of better or worse outcomes than expected is unlikely both because details of overall patient outcomes within the prior treatment period are not readily available to staff and because random variability renders it implausible that true effects would be [sic] distinguishable from random variation at a single ICU level.” (Supplement 2 of PEPTIC trial.^9^)

## Other information

### Item 24: Trial protocol

Where the full trial protocol and statistical analysis plan can be accessed, if available.

#### Explanation

Readers are referred to the CONSORT 2010 statement for examples and explanation.^3^ However, the CONSORT 2010 item was limited to only trial protocols. Statistical analysis plans, which provide technical details of the analysis (to allow for reproduction of the results), are now standard practice.^93^ As with trial protocols, authors should ensure that their statistical analysis plan (and any amendments) are accessible to users. Several mechanisms can be used to achieve this; for example, publishing these plans in journals (eg, *Trials*) and referencing the plan in the trial publication; including the plan as a supplementary file to the trial publication; uploading the plan to the clinical trial registry entry; or, depositing the plan to a general purpose or institutional open access repository (eg, Open Science Framework).

### Item 26: Research ethics review

Whether the study was approved by a research ethics committee, with identification of the review committee(s).

#### Explanation

The International Committee of Medical Journal Editors (ICMJE) requires that authors include a statement in the report about research ethics approval by an independent local, regional, or national review body.^69^ The CONSORT 2010 statement did not include an item on research ethics approval. However, extensions to the CONSORT statement for pilot studies^94^ and stepped wedge trials^12^ have introduced such an item. The relevant ethics committee(s) should be clearly identified, preferably together with the application or reference number. If a relevant research ethics committee granted an exemption from review, this exemption should be reported together with the justification for the exemption.

#### Example of item 26

“The National Healthcare Group Domain Specific Review Board [DSRB] approved the study, with waiver of informed consent from patients (DSRB number: 2017/00441).^35^

### Item 27: Data sharing

Where the individual de-identified participant data (including data dictionary), statistical code, and any other relevant documents or materials can be accessed.

#### Explanation

The increased call for data sharing for researchers, including clinical trialists, is now part of a larger ecosystem movement towards open science (eg, UNESCO (United Nations Educational, Scientific and Cultural Organization)^95^). The US National Institutes of Health have recently indicated that data sharing will become a normative practice for their grantees.^96^ The three federal funders in Canada now have a new mandate requiring data sharing by their grantees as of 2022.^97^ In Canada, data sharing will start by requiring grant applicants to describe their data management plans in their grant application, followed by actual data sharing at a later date. Similar data sharing requirements are elsewhere.^98^ While these developments are positive news, clinical trial data sharing is not yet a normative practice.

In the context of clinical trials, including CRXO trials, data sharing means that once the trial is finished and a report is made available (eg, preprint server; publication, preferably open access), the reporting includes sharing relevant trial information to facilitate bringing best evidence to best practice, to enhance and facilitate evidence syntheses^99^ and to help foster meta-research. This process typically includes the trial protocol, statistical analysis plan, or both (if not already publicly available; item 24), data, the analytical code that was used to arrive at the reported trial results, data dictionaries, and other material(s). Examples of the last item could include intervention materials; for example, in a trial examining the effects of freshly prepared school lunches (intervention condition) versus usual packed lunches from home (control condition), the authors would share details of the menu schedule, recipes, food groups, and nutrient content.

When sharing data, authors should be as open as possible, and as closed as necessary. How the data are shared might depend on funder policies or journal requirements. Data sharing recommendations and mandates are time consuming and require resources, which some authors might not be able to meet.^100^ Data sharing does not typically include sharing of signed patient consent forms. Authors should follow the FAIR (Findability, Accessibility, Interoperability, and Reuse of digital assets) principles when sharing clinical trial data.^101^

There have been concerns about the reproducibility of research, including clinical trials, for at least the past decade,^102^ but with little investigation into the reproducibility of trial results in biomedicine.^103^ Clinical trial data reuse can only happen if trialists share their data. Even during covid-19, trial data reuse did not happen^104^ and none of the covid-19 vaccine trialists shared the data underpinning their results.

#### Example of item 27

The publication of results for the ORGANIKO LIFE+CRXO trial included the following supporting information: study questionnaires, statistical analysis plan, data files (values in a format separated by commas), analysis script files (for the statistical package R), and methods for measuring the biomarkers.^33^

### Item 28: Patient and public involvement

Details of any patient and public involvement (PPI) in the design, conduct, and reporting of the trial; and, when applicable, other stakeholders’ involvement.

#### Explanation

PPI in research has been defined as research carried out with or by members of the public, rather than involvement as a participant in a randomised trial.^105^ In this definition, the public includes patients, potential patients, carers, and people who use health and social care services, as well as organisations that represent people who use these services.^105^ In the context of randomised trials, PPI might include input in trial oversight committees (eg, trial steering committee), defining the research questions, outcomes to be measured, visit schedules, methods of data collection, recruitment and consent procedures, lay write-up of the trials, and advocacy of the trial findings.^106^ ^107^

Many funding bodies encourage PPI in the clinical trials they fund,^108^ ^109^ and some journals now ask clinical trialists to explicitly report PPI in the trial (eg, *The BMJ*, *BMJ Open* 110). Despite such requirements, reporting of PPI has been very rare.^111^ Non-existent, incomplete, and inconsistent reporting can limit understanding of PPI in a particular trial and efforts to build a PPI evidence base (eg, cataloguing the types of PPI used in trials).^107^

The Guidance for Reporting Involvement of Patients and the Public 2 (GRIPP2) provides a checklist for reporting PPI in health and social care.^107^ The short form version (GRIPP2-SF) should be used to guide reporting in randomised trials (where PPI is not the primary focus of the trial). Some cluster trials (including CRXO trials) might have involvement from other stakeholders (eg, hospital administrative staff, health professionals) in addition to PPI.^108^ These stakeholders could be involved in various aspects of the trial (eg, advising on the content of the intervention, recruitment procedures), and their involvement should also be reported.

#### Example of item 28

*Example 1 (patient participant involvement)*—“The LUSTRUM [Limiting Undetected Sexually Transmitted infections to Reduce Morbidity] PPI (Patient and Public Involvement) Group consists of 27 lay people, with a broad mix of demographic characteristics and a range of sexual health experiences. The group were first involved in the design of the research at the grant application stage. Their opinions guided the trial protocol and topic guides for the process evaluation interviews. The trial is conducted by qualified healthcare professionals and, as such, it is not feasible for the group to undertake any aspects of the data collection. The group strongly supported the service-level consent design which reduces the burden of participating in the research. Lay summaries of our findings will be circulated to the group to gain their perspective and feedback on our results; this may inform further analytical decisions and inputs.”^112^

*Example 2 (other stakeholder involvement)*—“PREP-IT [Program of Randomized trials to Evaluate Pre-operative antiseptic skin solutions In orthopedic Trauma] stakeholders include patient advisors who experienced orthopedic trauma and/or surgery, and subsequent infection complications; professional-association representatives; a multidisciplinary healthcare team; and dissemination professionals. Stakeholders codeveloped the PREP-IT study concept and protocol following the 10-Step Framework.”^113^

## Conclusion

The CONSORT CRXO extension consolidates reporting items across relevant guidelines, modifying items as required, and includes additional items. The modified and new items address unique features of the CRXO design, as well as reflecting evolving requirements of the scientific community.^110^ ^114^ For CRXO trials to provide the best opportunity for informing policy and clinical practice, either directly, or through evidence synthesis products, complete and accurate reporting is required. The CONSORT CRXO extension is a first step to facilitating this. Journals, editors, and peer reviewers can also have a crucial role through endorsement and uptake of the guideline in editorial processes.

## Data Availability

The survey data produced for the present study are available on reasonable request to the authors (with ethics approval).

## Web extra

Extra material supplied by authors

Web appendix 1: Supplementary material 1

Web appendix 2: Supplementary material 2

Web appendix 3: Supplementary material 3

Web appendix 4: Checklist of information to include when reporting a cluster randomised crossover trial

## Notes

Contributors: JEM and ABF are joint lead authors. For the survey, JEM, MT, KH, JMG, and ABF sought ethics approval; JEM drafted proposed items and explanations, with input first from ABF, then MT and KH; and JEM administered the survey and analysed the data. For the consensus meeting, JEM prepared the materials with input from ABF, MT, and KH; JEM, ABF, and BG gave background presentations; JEM, ABF, KH, and MT presented proposed modifications to items and explanations; JMG chaired the meeting; all authors attended except for SJA, DM, and SE; JEM and Matthew J Page took notes; and JEM drafted the meeting summary. For the drafting of the extension, JEM revised the items and explanations with input first from ABF, then MT and KH. JEM led the drafting and editing of the article. All authors were involved in revising the article critically for important intellectual content. All authors approved the final version of the article. JEM is the guarantor of this work. The corresponding author attests that all listed authors meet authorship criteria and that no others meeting the criteria have been omitted.

Transparency: JEM and ABF affirm that the manuscript is an honest, accurate, and transparent account of the development of the CONSORT extension for CRXO trials; and that no important aspects have been omitted.

Provenance and peer review: Not commissioned; externally peer reviewed.
