# Supplementary material for: Reporting of cluster randomised crossover trials: extension of the CONSORT 2010 statement with explanation and elaboration
Source: BMJ. 2025 Jan 6;388:e080472. doi: 10.1136/bmj-2024-080472 (PMC11701780; doi:10.1136/bmj-2024-080472)
Supplement: Supplementary file 1 — Web appendix 1: Supplementary material 1 [file mckj080472.ww1.pdf]

Supplementary material 1 for ‘Reporting of cluster randomised crossover trials: extension of the CONSORT 2010 statement with explanation and elaboration’

McKenzie JE*, Taljaard M, Hemming K, Arnup SJ, Giraudeau B, Eldridge S, Hooper R, Kahan BC, Li T, Moher D, Grimshaw JM, Forbes AB. BMJ 2025;388:e080472. doi:10.1136/bmj-2024-080472

* Correspondence to: Professor Joanne McKenzie, Methods in Evidence Synthesis Unit, School of Public Health and Preventive Medicine, Monash University, 553 St Kilda Road, Melbourne, Victoria 3004, Australia
Email: joanne.mckenzie@monash.edu

Table of contents:

Section 1. Development of the CONSORT extension for cluster randomised crossover trials 2

Section 2. Survey for the CONSORT extension for cluster randomised crossover trials 4

Section 1. Development of the CONSORT extension for cluster randomised crossover trials

Development of this extension was based on guidance for developing health research reporting guidelines (1). We registered our intent to develop the CONSORT cluster randomised crossover (CRXO) extension on the Enhancing the QUAlity and Transparency Of health Research (EQUATOR) network (23 June 2017). We 1) undertook a systematic review to examine the quality of reporting of CRXO trials, 2) drafted proposed items and explanations, 3) undertook a survey to seek input on the proposed items and explanations, 4) convened an in-person consensus meeting to discuss the items, and accordingly, 5) revised the items and explanations. Funding to support the in-person consensus meeting was obtained through an Australian National Health and Medical Research Council project grant (GNT1108283). Ethics approval for the survey was obtained from the Monash University Human Research Ethics Committee (approval number 13429).

1) Results from systematic review examining the quality of reporting of CRXO trials
We undertook a systematic review to assess the design and statistical methods used in CRXO trials, and the quality of reporting, identifying 83 articles meeting our inclusion criteria (up to December 2014) (2, 3). Key areas of incomplete reporting included: identifying the CRXO trial as such in the title or abstract; reporting a rationale for choosing the CRXO design; providing a schematic of the design; discussing the potential for carryover; providing the method for sample size calculation, or a justification for no calculation; reporting the method used to generate the random allocation schedule; and, reporting of estimates of correlation coefficients between outcomes of participants from the same cluster for each primary outcome. Moreover, we determined that the potential for recruitment or identification bias could not be assessed in up to 30% of trials. Furthermore, while presentation of the baseline characteristics was common, there was inconsistency in the presentation.

2) Drafting of proposed items and explanations
Draft CRXO items and explanations were written, informed by the results of the systematic review, the items and explanations from other relevant statements, and our knowledge and review of the methodological literature. The statements of most relevance were the CONSORT 2010 statement, and the extensions for cluster, stepped-wedge and crossover (the version of the latter was under development) designs. The items were drafted and revised by the core team (JEM, ABF, MT, KH) and formed the basis of the survey.

3) Survey seeking input on proposed items and explanations
Potential participants were identified by the core team and were selected to ensure representation from trialists with CRXO experience, methodologists and statisticians with expertise in multiple-period and cluster designs, ethicists, journal editors and contributors to relevant CONSORT design extensions. We invited 101 people via email to participate, giving them four weeks to respond (28th July to 24th August, 2018), with a reminder sent at two weeks. Of the 101 invitees, 6 emails bounced, 3 opted out (because they did not feel sufficiently qualified to participate), and 1 had retired. Of the remaining 91, 53 completed the survey (45 full and 8 partial completions).

The survey (created using Qualtrics online survey software (4)) was structured using the 25 items of the CONSORT 2010 statement, plus an additional item on research ethics review from the CONSORT extension for stepped-wedge designs (5) (see Section 2 for a copy of the survey). We presented the CONSORT 2010 item, items from the relevant design extensions, and the proposed item and explanation for the CRXO design. For each proposed item and explanation, participants were asked whether they agreed; agreed – but with some change; disagreed; or didn't know. For some items, an explanation was not presented, instead referring readers to the relevant CONSORT statements, and the survey participants were asked whether an explanation beyond the referenced statement was required.

4) Consensus meeting
We held a one-day consensus meeting in Melbourne, Australia (31st August 2018). Participants of the consensus meeting (10 people) included the core team, those with expertise in multiple-period and cluster designs, statisticians and methodologists, and developers of relevant reporting guidelines (stepped-wedge and crossover) (BG, RH, BCK, TL, ELT, JMG). Some participants also had roles as journal editors.

A subset of the 26 items and explanations were discussed; items for which there was high agreement on the survey and no important issues raised were excluded from discussion. Each item was introduced by one of the core team and following this, any major comments from the survey on the item or explanation were presented, along with proposed revisions (which had been formulated by JEM, ABF and MT). We then had open discussion of the revisions, chaired by JMG.

5) Revision of the items and explanations
Following the meeting, the core team revised the items and explanations, via email, virtual meetings and an in-person meeting (attended by JEM, ABF, MT in November 2019). During this process, two new items were added (items 27 and 28) to address suggestions made in the survey, and a separate checklist for reporting abstracts for CRXO trials was drafted, reflecting specific reporting requirements for CRXO trials. Multiple drafts were circulated to the authorship team, and throughout the writing and editing process, the authors integrated key developments in the design and analysis of CRXO trials that had arisen in the literature over the course of developing the guideline. The extension was approved by a member of the CONSORT Executive (DM), who was a member of the CRXO team.

References

1. Moher D, Schulz KF, Simera I, Altman DG. Guidance for Developers of Health Research Reporting Guidelines. PLoS Med. 2010;7(2):e1000217.
2. Arnup SJ, Forbes AB, Kahan BC, Morgan KE, et al. The quality of reporting in cluster randomised crossover trials: proposal for reporting items and an assessment of reporting quality. Trials. 2016;17(1):575.
3. Arnup SJ, Forbes AB, Kahan BC, Morgan KE, et al. Appropriate statistical methods were infrequently used in cluster-randomized crossover trials. Journal of clinical epidemiology. 2016;74:40-50.
4. Qualtrics. Copyright year. 2023.: Qualtrics. Provo, Utah, USA. https://www.qualtrics.com; 2005 [first release].
5. Hemming K, Taljaard M, McKenzie JE, Hooper R, et al. Reporting of stepped wedge cluster randomised trials: extension of the CONSORT 2010 statement with explanation and elaboration. Bmj. 2018;363:k1614.

Section 2. Survey for the CONSORT extension for cluster randomised crossover trials

Background

The Consolidated Standards of Reporting Trials (CONSORT) statement provides minimum set of recommendations for reporting randomised trials. The statement was developed to facilitate complete and transparent reporting of aspects of trial design, conduct, analysis and results. Extensions of the CONSORT statement have been developed, or are in development, to give additional guidance for specific designs, such as the cluster, stepped-wedge, and crossover randomised trials. However, there is currently no extension for the cluster randomised crossover (CRXO) design. The aim of this project is to identity a set of recommended reporting items for CRXO trials.

Scope of CONSORT for CRXO randomised trials

The statement is being developed for trials in which:

- the allocation of the treatments is to clusters of individuals;
- each cluster receives each treatment in a sequence of time (conventional crossover design), or at least some clusters crossover from one treatment to another (e.g. a two-treatment-four-sequence design AA, AB, BA, BB);
- at least some clusters cross each way between at least two treatments (e.g. one cluster receives AB and one cluster receives BA); and,
- the clusters are randomly allocated to the sequences of treatments.

The individuals in the different periods may be the same people, different people, or a mixture.

Instructions for completing this survey

The survey will proceed through each of the standard CONSORT items in turn. You will be provided with a proposed corresponding item for the CRXO extension statement and a proposed explanation. To prompt thinking, items from the other related CONSORT extension statements are also provided (including, cluster, crossover, and the stepped-wedge).

For each of the proposed items, you will be asked to indicate whether you:

- Agree with the item
- Agree – but some change to the wording required (please explain)
- Disagree (please explain)
- Don't know

For the *content* of the proposed explanation of each item, you will be asked to indicate whether you:

- Agree
- Agree – but some additional rationale could be provided (please explain)
- Disagree (please explain)
- Don't know

At this stage we are seeking your advice on the *content* rather than the wording of the explanations, which is likely to change once the items have been agreed.

For some items no explanation is provided, and instead readers are referred to the relevant CONSORT statement(s). For these items, you will be asked whether an explanation beyond the referenced statement should be provided. For some of these items, a 'consideration' will be presented to prompt thinking about whether an extension to the explanation might be needed. These 'considerations' are based on the explanations from other CONSORT extension statements.

The final CRXO statement will include examples of good reporting for each item. These are not presented here to reduce the time taken to complete the survey.

The complete survey (including the list of references cited in the explanations) can be downloaded here.

Section/ Topic: Title and Abstract

Item 1a: Title

Standard CONSORT item:

Identification as a randomised trial in the title.

CONSORT cluster extension:

Title Identification as a cluster randomised trial in the title.

CONSORT crossover randomised trial extension [version 16/07/2018]**:**

Identification as a randomised crossover trial in the title.

CONSORT stepped-wedge cluster randomised trials extension [BMJ accepted]**:**

Identification as a stepped-wedge cluster randomised trial in the title.

Proposed checklist item for CRXO trial (modified item):

Identification as a cluster randomised crossover trial in the title.

Proposed explanation: Identification of the study design in the title facilitates appropriate indexing in bibliographic databases (6), searching, and ensures ease of identification of randomised trials for potential inclusion in evidence synthesis products (e.g. systematic reviews). Further, alerting readers to the study design early in the article allows them to consider implications for the analysis methods and any potential biases. A review of CRXO trials found that only 7/83 (8%) were identified as such in the title (2).

Do you agree with the proposed checklist item?

Agree

Agree – but some change to the wording required (please explain)

Disagree (please explain)

Don't know

Do you agree with the *content* of the proposed explanation?

Agree

Agree – but some additional rationale could be provided (please explain)

Disagree (please explain)

Don't know

Further comments:

Section/ Topic: Introduction

Item 2a: Background

Standard CONSORT:

Scientific background and explanation of rationale.

CONSORT cluster extension:

Rationale for using a cluster design

CONSORT crossover randomised trial extension [version 16/07/2018]**:**

No modification suggested.

CONSORT stepped-wedge cluster randomised trials extension [BMJ accepted]**:**

Scientific background. Rationale for using a cluster design and rationale for using a stepped-wedge design.

Proposed checklist item for CRXO trial (standard CONSORT item):

Scientific background and explanation of rationale.

(Note that providing a rationale for using a cluster crossover design is proposed to be included in Item 3a)

Proposed explanation: Readers are referred to the CONSORT statement for examples and explanation (6).

Do you agree with the proposed checklist item?

Agree

Agree – but some change to the wording required (please explain)

Disagree (please explain)

Don't know

Does this item require an explanation beyond the referenced CONSORT statement?

Yes (please explain reasons)

No

Don't know

Further comments:

Section/ Topic: Introduction

Item 2b: Objective
Standard CONSORT item:
Specific objectives or hypotheses.

CONSORT cluster extension:
Whether objectives pertain to the cluster level, the individual participant level or both.

CONSORT crossover randomised trial extension [version 16/07/2018]**:**
No modification suggested.

CONSORT stepped-wedge cluster randomised trials extension [BMJ accepted]**:**
Specific objectives or hypotheses.

Proposed checklist item for CRXO trial (standard CONSORT item):
Specific objectives or hypotheses.

Proposed explanation: Readers are referred to the CONSORT statement for examples and explanation (6).

Consideration: An explanation could include:

- Clearly specifying if the intervention's effects will be on process outcomes (e.g. systems changes, clinician performance) or patient outcomes; and,
- Whether the study is to show superiority of the intervention condition, non-inferiority or equivalence.

Do you agree with the proposed checklist item?

Agree

Agree – but some change to the wording required (please explain)

Disagree (please explain)

Don't know

Does this item require an explanation beyond the referenced CONSORT statement (for example, by addressing points in the *Consideration* above)?

Yes (please explain reasons)

No

Don't know

Further comments:

Section/ Topic: Methods Trial design

Item 3a: Trial design

Standard CONSORT item:

Description of trial design (such as parallel, factorial) including allocation ratio.

CONSORT cluster extension:

Definition of cluster and description of how the design features apply to the clusters.

CONSORT crossover randomised trial extension [version 16/07/2018]**:**

Rationale for a crossover design. Description of the design features including allocation ratio, especially the number and duration of periods, duration of washout period and consideration of carryover effect.

CONSORT stepped-wedge cluster randomised trials extension [BMJ accepted]**:**

Description and diagram of trial design including definition of cluster, number of sequences, number of clusters randomised to each sequence, number of periods, duration of time between each step, and whether the participants assessed in different periods are the same people, different people, or a mixture.

Proposed checklist item for CRXO trial (modified item):

Rationale for a cluster crossover design. Description of trial design including definition of cluster, number and duration of periods, number of sequences, number of clusters randomised to each sequence, duration of any washout periods, and whether the participants assessed in different periods are the same people, different people, or a mixture of the two. Consideration of carryover effect. Diagram of trial when there are more than two periods and/or treatment conditions.

Proposed explanation: A rationale for the choice of trial design should be provided. For a CRXO trial, this should include justification of the cluster and crossover aspects of the design (2). The cluster randomisation aspect will likely increase the required number of participants, as compared to an individually randomised trial. Thus, more participants will be exposed to an intervention of unknown effectiveness than if individual randomisation was feasible. The crossover element may increase the potential for bias as compared to a parallel (individual or cluster) designs. This may arise from carryover effects, an increased risk of selective identification and recruitment of participants (since it may be harder to blind those responsible for recruiting in the second period) or an increased risk of cluster withdrawal (due to additional burden of participating in multiple periods).

Possible justifications for adopting a CRXO design include the necessity to conduct a cluster randomised trial, which would otherwise be infeasible as a parallel design, due to the availability of too few clusters to achieve the desired power (7); to cross over to increase the statistical efficiency (with associated benefits such as minimising the number of participants exposed to the interventions, and reducing the cost) (8, 9); and, guaranteed receipt of the intervention acting as an incentive to cluster participation (10).

A key requirement of the crossover design is that the effect of an intervention given in one period does not carry over into the next period. Therefore, methods for managing potential carryover (e.g the use of washout periods (along with justification for the duration), different participants in each period, blinding of trialists involved in the delivery of the intervention), or justification for why carryover is expected to be negligible, should be provided. In a 2x2 crossover design, carryover is particularly problematic since it cannot be distinguished from a treatment by period interaction (11).

Providing specific details of CRXO design is important for allowing assessment of the potential for bias, and whether the sample size and analysis methods were appropriate. This should include providing a definition of the cluster, the number and duration of periods, the number of sequences, and the number

of clusters randomised to each sequence. Further, whether the participants assessed in different periods are the same individuals, different individuals, or a mixture of the two.

For complex crossover designs (i.e. more than two periods or treatment conditions, or both) a diagram can efficiently and clearly communicate the design details. Key details to depict include all sequences of treatments, any washout periods, and the number of clusters allocated to each sequence. Such a diagram will clearly signal any imbalance in the treatments evaluated in each period, and therefore the need to control for period effects.

Do you agree with the proposed checklist item?

Agree

Agree – but some change to the wording required (please explain)

Disagree (please explain)

Don’t know

Do you agree with the *content* of the proposed explanation?

Agree

Agree – but some additional rationale could be provided (please explain)

Disagree (please explain)

Don’t know

Further comments:

Section/ Topic: Methods Trial design

Item 3b: Trial design
Standard CONSORT item:
Important changes to methods after trial commencement (such as eligibility criteria), with reasons.

CONSORT cluster extension:
No modification suggested.

CONSORT crossover randomised trial extension [version 16/07/2018]**:**
No modification suggested.

CONSORT stepped-wedge cluster randomised trials extension [BMJ accepted]**:**
No modification suggested.

Proposed checklist item for CRXO trial (standard CONSORT item):
Important changes to methods after trial commencement (such as eligibility criteria), with reasons.

Proposed explanation: Readers are referred to the CONSORT statement for examples and explanation (6).

Consideration: An explanation could include:

- Reporting a test for carryover when the results from this test mean that only first period data is used.

Do you agree with the proposed checklist item?

Agree

Agree – but some change to the wording required (please explain)

Disagree (please explain)

Don't know

Does this item require an explanation beyond the referenced CONSORT statement (for example, by addressing the point in the *Consideration* above)?

Yes (please explain reasons)

No

Don't know

Further comments:

Section/ Topic: Methods Participants

Item 4a: Participants

Standard CONSORT item:
Eligibility criteria for participants.

CONSORT cluster extension:
Eligibility criteria for clusters.

CONSORT crossover randomised trial extension [version 16/07/2018]**:**
No modification suggested.

CONSORT stepped-wedge cluster randomised trials extension [BMJ accepted]**:**
Eligibility criteria for clusters and participants.

Proposed checklist item for CRXO trial (stepped-wedge CONSORT item):
Eligibility criteria for clusters and participants.

Proposed explanation: A comprehensive description of the eligibility criteria is required to help readers determine the population to whom the results of the trial can be generalised. The criteria should be framed in such a way that a reader can recognise whether the trial includes typical or atypical clusters and participants (12). For clustered trials, inclusion and exclusion criteria need to be reported for both clusters and participants. In addition, in some cluster trials, there may be multiple levels of participants (e.g. emergency department staff and patients attending the emergency departments), and eligibly criteria should be reported for each. In a review of 34 cluster trials, most reports were noted to contain some information about cluster eligibility (13).

Do you agree with the proposed checklist item?

Agree

Agree – but some change to the wording required (please explain)

Disagree (please explain)

Don’t know

Do you agree with the *content* of the proposed explanation?

Agree

Agree – but some additional rationale could be provided (please explain)

Disagree (please explain)

Don’t know

Further comments:

Section/ Topic: Methods Intervention

<u>Item 5: Intervention</u>

Standard CONSORT item:

The interventions for each group with sufficient details to allow replication, including how and when they were actually administered.

CONSORT cluster extension:

Whether interventions pertain to the cluster level, the individual participant level or both.

CONSORT crossover randomised trial extension [version 16/07/2018]**:**

The interventions with sufficient details to allow replication, including how and when they were actually administered.

CONSORT stepped-wedge cluster randomised trials extension [BMJ accepted]**:**

The intervention and control conditions with sufficient details to allow replication, including whether the intervention was maintained or repeated, and whether it was delivered at the level of the cluster, the individual, or both.

Proposed checklist item for CRXO trial (modified item):

The treatment conditions with sufficient details to allow replication, and whether they were delivered at the level of the cluster, the individual, or both.

Proposed explanation: A complete description of interventions is necessary for reliable implementation of those found to be effective (14). For interventions that are found to (conclusively) have small effects, complete reporting reduces the chance that the same intervention will again be evaluated, but allows identification of aspects of the intervention that might be modified in a future evaluation (10). Complete description also allows assessment of the potential for carryover effects. That is, the possibility that the effects of the interventions persist into a subsequent period once they are withdrawn. Unlike other cluster designs (parallel, stepped-wedge), the CRXO design is not suitable for evaluating interventions where their effects persist, such as educational interventions targeted at changing clinicians' practice.

The TIDieR (Template for Intervention Description and Replication) checklist, provides an extension of the CONSORT statement (item 5) (6), for describing interventions (14). The checklist consists of 12 recommended items that should be reported for all the evaluated treatment conditions, irrespective of whether they are active or control ('standard' or 'usual' care). For complex interventions with multiple components, detail should be provided for each component (14). Such detailed description facilitates investigation of which components are the 'active' ingredients when the results from a collection of studies are combined using meta-regression methodology in systematic reviews (15).

For cluster trials, reporting whether the intervention is delivered at the cluster level, the individual participant level, or both, is important. This information allows assessment of the coverage of the intervention, and what consent procedures should be in place (items 10c and 26). In CRXO trials, interventions delivered at the individual participant level are quite commonly assessed (e.g. daily bathing of critically ill children in paediatric intensive-care units with chlorhexidine gluconate compared with standard bathing practices), since these can be withdrawn (3).

Do you agree with the proposed checklist item?

Agree

Agree – but some change to the wording required (please explain)

Disagree (please explain)

Don’t know

Do you agree with the *content* of the proposed explanation?

Agree

Agree – but some additional rationale could be provided (please explain)

Disagree (please explain)

Don’t know

Further comments:

Section/ Topic: Methods Outcomes

Item 6a: Outcomes

Standard CONSORT item:

Completely defined pre-specified primary and secondary outcome measures, including how and when they were assessed.

CONSORT cluster extension:

Whether outcome measures pertain to the cluster level, the individual participant level or both.

CONSORT crossover randomised trial extension [version 16/07/2018]**:**

No modification suggested.

CONSORT stepped-wedge cluster randomised trials extension [BMJ accepted]**:**

No modification suggested.

Proposed checklist item for CRXO trial (standard CONSORT item):

Completely defined pre-specified primary and secondary outcome measures, including how and when they were assessed.

Proposed explanation: Readers are referred to the CONSORT statement for examples and explanation (6).

Consideration: An explanation could include:

- Reporting whether measurements could be made on different participants in each period (i.e. cross-sectional); the same participants (i.e. cohort design); or a mixture.
- Whether data are collected at the level of the cluster (either because of an outcome being a true cluster level outcome, or only aggregated data from data from individuals is available).
- Clear reporting of the timing of follow-up assessments allows assessment of whether observations in the treatment period were fully exposed to the intervention, or whether observations in the control condition were contaminated.

Do you agree with the proposed checklist item?

Agree

Agree – but some change to the wording required (please explain)

Disagree (please explain)

Don't know

Does this item require an explanation beyond the referenced CONSORT statement (for example, by addressing points in the *Consideration* above)?

Yes (please explain reasons)

No

Don't know

Further comments:

Section/ Topic: Methods Outcomes

Item 6b: Changes to outcomes

Standard CONSORT item:

Any changes to trial outcomes after the trial commenced, with reasons.

CONSORT cluster extension:

No modification suggested.

CONSORT crossover randomised trial extension [version 16/07/2018]**:**

No modification suggested.

CONSORT stepped-wedge cluster randomised trials extension [BMJ accepted]**:**

No modification suggested.

Proposed checklist item for CRXO trial (standard CONSORT item):

Any changes to trial outcomes after the trial commenced, with reasons.

Proposed explanation: Readers are referred to the CONSORT statement for examples and explanation (6).

Do you agree with the proposed checklist item?

Agree

Agree – but some change to the wording required (please explain)

Disagree (please explain)

Don't know

Does this item require an explanation beyond the referenced CONSORT statement?

Yes (please explain reasons)

No

Don't know

Further comments:

Section/ Topic: Methods Sample size

Item 7a: Sample size

Standard CONSORT item:

How sample size was determined.

CONSORT cluster extension:

Method of calculation, number of clusters(s) (and whether equal or unequal cluster sizes are assumed), cluster size, a coefficient of intra-cluster correlation (ICC or k), and an indication of its uncertainty.

CONSORT crossover randomised trial extension [version 16/07/2018]**:**

How sample size was determined, accounting for within participant variability.

CONSORT stepped-wedge cluster randomised trials extension [BMJ accepted]**:**

How sample size was determined. Method of calculation and relevant parameters with sufficient detail so the calculation can be replicated (Table 6). Assumptions made about correlations between outcomes of participants from the same cluster.

Proposed checklist item for CRXO trial (stepped-wedge CONSORT item):

How sample size was determined. Method of calculation and relevant parameters with sufficient detail so the calculation can be replicated (Table 6). Assumptions made about correlations between outcomes of participants from the same cluster.

Proposed explanation: Reporting how a sample size calculation has been performed is important for replicability, transparency (16) and scientific and ethical reasons (17). A key advantage of a CRXO design is that it may offset some of the loss in precision that arises from clustering through the removal of cluster-specific variation (18). Incorporation of both the cluster-specific variation and the cluster-period level variation (or equivalently, within-period and between-period correlations for the 2x2 design) in the sample size calculation is therefore required, and are the elements that distinguish CRXO from parallel cluster design calculations.

The method for sample size calculation should be provided (either referenced or described), specifically noting how the methodology accounts for both the cluster randomisation and the multiple period aspects of the design (e.g. (8, 9)). In addition to reporting the usual parameters required for sample size calculation (i.e. specified target difference between intervention groups, significance level (type I error level), power (type II error level), and for continuous outcomes, the standard deviation of the measurements) the following should be reported (2):

- number of clusters, number of periods, and the number of participants per cluster-period (and whether account of unequal cluster sizes has been made), noting which are assumed and which are determined from the same size calculation;
- for a 2x2 crossover design, a within-period intra -cluster correlation (WP-ICC) and a between period intra-cluster correlation (BP-ICC) (or a cluster autocorrelation coefficient (19)). The WP-ICC quantifies the similarity of the outcomes in the same cluster period, while the BP-ICC quantifies the similarity of outcomes in the same cluster, but in different periods (9). For designs with more than two periods, full detail of the assumed within-cluster correlation structure across all periods should be reported (e.g. exponential decay (20));
- any allowance made for repeated measurements taken from the same participants (if relevant); and,
- justification for the choice of parameter values and any constraints on the number of clusters, number of periods, or number of participants per cluster-period.

Often sample size parameters (e.g. WP-ICC, BP-ICC) are based on estimates with large uncertainty. Therefore, providing an indication of the sensitivity of the sample size, or power, to the assumed parameter values could be of merit. If no power calculation was performed, this should be reported.

Retrospective power calculations based on the results of the trial are of little merit and are discouraged (6, 21).

Reporting of sample size calculations, and with sufficient details to replicate them, could be improved in CRXO trials (3). In a review of CRXO trials, only 58% (53/91) provided a sample size calculation; of these, 74% (39/53) provided sufficient detail to allow replication. In only 10% of trials (9/91) was a sample size methodology used that was appropriate for the design.

Do you agree with the proposed checklist item?

Agree

Agree – but some change to the wording required (please explain)

Disagree (please explain)

Don’t know

Do you agree with the *content* of the proposed explanation?

Agree

Agree – but some additional rationale could be provided (please explain)

Disagree (please explain)

Don’t know

Further comments:

Section/ Topic: Methods Sample size

Item 7b: Interim analyses

Standard CONSORT item:

When applicable, explanation of any interim analyses and stopping guidelines.

CONSORT cluster extension:

No modification suggested.

CONSORT crossover randomised trial extension [version 16/07/2018]**:**

No modification suggested.

CONSORT stepped-wedge cluster randomised trials extension [BMJ accepted]**:**

No modification suggested.

Proposed checklist item for CRXO trial (standard CONSORT item):

When applicable, explanation of any interim analyses and stopping guidelines.

Proposed explanation: Readers are referred to the CONSORT statement and the extension to the CONSORT statement for examples and explanation (6, 16).

Do you agree with the proposed checklist item?

Agree

Agree – but some change to the wording required (please explain)

Disagree (please explain)

Don't know

Does this item require an explanation beyond the referenced CONSORT statements?

Yes (please explain reasons)

No

Don't know

Further comments:

Section/ Topic: Methods Randomisation – Sequence generation

Item 8a: Sequence generation

Standard CONSORT item:

Method used to generate the random allocation sequence.

CONSORT cluster extension:

No modification suggested.

CONSORT crossover randomised trial extension [version 16/07/2018]**:**

No modification suggested.

CONSORT stepped-wedge cluster randomised trials extension [BMJ accepted]**:**

Method used to generate the random allocation to the sequences of treatments.

Proposed checklist item for CRXO trial (stepped-wedge CONSORT item):

Method used to generate the random allocation to the sequences of treatments.

Proposed explanation: In a CRXO trial, clusters are randomly allocated to a *sequence of treatments*, where the sequence defines the order in which each cluster will receive the treatments. For example, in a two-treatment two-period design, the sequence of treatments to which a cluster is randomly allocated may be either treatment A followed by treatment B; or, treatment B followed by treatment A. Here the term 'sequence' conveys something additional to its usage in a parallel (individual or cluster) randomised trials, where 'sequence' refers only to the allocation of units to *a single treatment* (e.g. treatment A or treatment B) (Box 1).

The list of candidate sequences and the method used to generate the random allocation of clusters to these sequences needs to be sufficiently described so that an assessment can be made as to its adequacy. Use of general terms such as 'random', 'randomisation', 'random allocation' are insufficient. Details of the method are required (e.g. computer generated random numbers, coin tossing).

Box 1: Distinguishing the meanings of 'sequence'

	Allocation *sequence* for six clusters					
	1	2	3	4	5	6
Sequence of treatments	BA	BA	AB	BA	AB	AB

Do you agree with the proposed checklist item?

Agree

Agree – but some change to the wording required (please explain)

Disagree (please explain)

Don't know

Do you agree with the *content* of the proposed explanation?

Agree

Agree – but some additional rationale could be provided (please explain)

Disagree (please explain)

Don’t know

Further comments:

Section/ Topic: Methods Randomisation – Sequence generation

Item 8b: Sequence generation

Standard CONSORT item 8b: Sequence generation

Type of randomisation; details of any restriction (such as blocking and block size).

CONSORT cluster extension:

Details of stratification or matching if used

CONSORT crossover randomised trial extension [version 16/07/2018]**:**

No modification suggested.

CONSORT stepped-wedge cluster randomised trials extension [BMJ accepted]**:**

Type of randomisation; details of any constrained randomisation or stratification if used.

Proposed checklist item for CRXO trial (modified item):

Type of randomisation; details of any stratification if used.

Proposed explanation: In a CRXO trial, randomisation of the clusters to the sequences of treatments is often done at a single point in time before the trial commences. In this circumstance, the randomisation can be balanced (restricted) such that an equal number of clusters are allocated to each of the sequences of treatments. This can be achieved using permuted blocks (e.g. for an AB/BA design, a block of size 6 would have 3 clusters allocated to AB and 3 clusters allocated to BA). When the design consists of sequences with their 'duals' (e.g. when the design has an ABB sequence it also has a BAA sequence), then such balance will limit the potential impact of period effects.

Stratification in CRXO trials may also be used when there are clusters with distinct characteristics (e.g. different case-mix, different hospital protocols for the delivery of care) which are related to the trial outcomes. In stratified designs, allocation sequences are generated independently within each stratum. In combination, permuted blocks are used to maintain balance in the number of clusters allocated to the sequences of treatments.

When reporting the type of randomisation, it is important to indicate whether clusters were allocated at one time (or not), and whether simple or restricted randomisation (e.g. permuted blocks) was used. If permuted block randomisation was used, details should be provided on how the blocks were generated, the block size(s), and whether the block size was fixed or randomly varied. If stratification was used, details should be provided about the stratification variables, the categorisation cut-off values, and the method used for restriction (6).

In a review of CRXO trials, only 38% (35/91) provided sufficient information to replicate the randomisation (3). Thirty (33%) of the trials used covariates in the randomisation.

Do you agree with the proposed checklist item?

Agree

Agree – but some change to the wording required (please explain)

Disagree (please explain)

Don't know

Do you agree with the *content* of the proposed explanation?

Agree

Agree – but some additional rationale could be provided (please explain)

Disagree (please explain)

Don’t know

Further comments:

Section/ Topic: Methods Randomisation –Allocation concealment

Item 9: Allocation concealment

Standard CONSORT item:

Mechanism used to implement the random allocation sequence (such as sequentially numbered containers), describing any steps taken to conceal the sequence until interventions were assigned.

CONSORT cluster extension:

Specification that allocation was based on clusters rather than individuals and whether allocation concealment (if any) was at the cluster level, the individual participant level or both.

CONSORT crossover randomised trial extension [version 16/07/2018]**:**

No modification suggested.

CONSORT stepped-wedge cluster randomised trials extension [BMJ accepted]**:**

Specification that allocation was based on clusters; description of any methods used to conceal the allocation from the clusters until after recruitment.

Proposed checklist item for CRXO trial (stepped-wedge CONSORT item):

Specification that allocation was based on clusters; description of any methods used to conceal the allocation from the clusters until after recruitment.

Proposed explanation: The benefits of randomisation are only realised when randomisation is properly implemented. This requires generation of a random allocation sequence, and concealment of upcoming assignments from the clusters (e.g. the 'gatekeeper' or 'guardian') and from those responsible for recruiting the clusters, until after recruitment (known as allocation concealment) (22). The randomisation process in CRXO trials may involve randomising clusters sequentially, in batches, or all at once (23). The strategies implemented to conceal the allocation will differ depending on the randomisation process, and should be completely described. As with other cluster randomised trials, allocation concealment is likely to be preserved when randomisation occurs at a single point in time.

In cluster randomised trials, participants may be recruited (or identified) after the clusters have been allocated. This can lead to selection bias arising from selective recruitment (or identification) of participants across treatment conditions when those responsible for the recruitment are aware of the cluster's allocation in a particular period. In this statement, the process for recruitment of individual level participants is considered separately (item 10b) to the randomisation process.

Use of the 'timeline cluster tool', which depicts the time sequence of trial processes (including the randomisation process) and the blinding status of participants and trial staff at each stage, allows readers to easily assess threats to the internal validity of the trial (24).

Do you agree with the proposed checklist item?

Agree

Agree – but some change to the wording required (please explain)

Disagree (please explain)

Don't know

Do you agree with the *content* of the proposed explanation?

Agree

Agree – but some additional rationale could be provided (please explain)

Disagree (please explain)

Don’t know

Further comments:

Section/ Topic: Methods Randomisation – Implementation

Item 10: Implementation

Standard CONSORT item:

Who generated the random allocation sequence, who enrolled participants, and who assigned participants to interventions.

CONSORT cluster extension:

Replace by 10a, 10b and 10c.

CONSORT crossover randomised trial extension [version 16/07/2018]**:**

No modification suggested.

CONSORT stepped-wedge cluster randomised trials extension [BMJ accepted]**:**

Replace by 10a, 10b and 10c.

Proposed checklist item for CRXO trial (stepped-wedge and cluster CONSORT item):

Replace by 10a, 10b and 10c.

Proposed explanation: Consistent with the CONSORT extension for other clustered designs (10, 16), it is important that all steps in the implementation of the randomisation and recruitment processes for both clusters and individual level participants are described. Details of the allocation and enrolment process of the clusters is described in item 10a, with the corresponding detail provided for participants in item 10b. The consent process for participants, which is an integral component of the enrolment process, is described in item 10c.

Do you agree with the proposed checklist item?

Agree

Agree – but some change to the wording required (please explain)

Disagree (please explain)

Don't know

Do you agree with the *content* of the proposed explanation?

Agree

Agree – but some additional rationale could be provided (please explain)

Disagree (please explain)

Don't know

Further comments:

Section/ Topic: Methods Randomisation – Implementation

Item 10a: Implementation

Standard CONSORT item:

Not included in original CONSORT statement.

CONSORT cluster extension:

Who generated the random allocation sequence, who enrolled clusters, and who assigned clusters to interventions.

CONSORT crossover randomised trial extension [version 16/07/2018]**:**

Not included in crossover CONSORT extension.

CONSORT stepped-wedge cluster randomised trials extension [BMJ accepted]**:**

Who generated the randomisation schedule, who enrolled clusters, and who assigned clusters to sequences.

Proposed checklist item for CRXO trial (stepped-wedge CONSORT item):

Who generated the randomisation schedule, who enrolled clusters, and who assigned clusters to sequences.

Proposed explanation: Reporting who generated the randomisation schedule, who enrolled the clusters, and who assigned the clusters to the sequences is important for allowing assessment of the potential for selection bias. There should be separation between the person who generates the allocation schedule and those who enrol and assign the clusters to the sequences. Failure to separate these processes risks potential subversion of the randomisation schedule and potential biased estimates of treatment effect.

Do you agree with the proposed checklist item?

Agree

Agree – but some change to the wording required (please explain)

Disagree (please explain)

Don't know

Do you agree with the *content* of the proposed explanation?

Agree

Agree – but some additional rationale could be provided (please explain)

Disagree (please explain)

Don't know

Further comments:

Section/ Topic: Methods Randomisation – Implementation

Item 10b: Implementation

Standard CONSORT item:

Not included in original CONSORT statement.

CONSORT cluster extension:

Mechanism by which individual participants were included in clusters for the purposes of the trial (such as complete enumeration, random sampling).

CONSORT crossover randomised trial extension [version 16/07/2018]**:**

Not included in crossover CONSORT extension.

CONSORT stepped-wedge cluster randomised trials extension [BMJ accepted]**:**

Mechanism by which individual participants were included in clusters for the purposes of the trial (such as complete enumeration or random sampling; continuous recruitment or ascertainment, or recruitment at a fixed point in time), including who recruited or identified participants.

Proposed checklist item for CRXO trial (stepped-wedge CONSORT item):

Mechanism by which individual participants were included in clusters for the purposes of the trial (such as complete enumeration or random sampling; continuous recruitment or ascertainment, or recruitment at a fixed point in time), including who recruited or identified participants.

Proposed explanation: As with other cluster randomised designs (10, 16), when collecting individual level observations, there are different sources from which the data can be obtained (e.g. directly from the patient, medical records), different sampling strategies (e.g. complete enumeration, random sample), and different timings of the data collection (e.g. continuously, a fixed point in time). The mechanisms for collecting individual level observations have differing risks of selection bias.

In some CRXO trials, data is not directly sought from participants, but instead obtained from routinely collected sources (such as hospital medical records). In these trials, participants are identified, but are not recruited to the trial. Alternatively, data may be directly sought from participants through data assessments or questionnaires, in which case, participants will be recruited into the trial. Recruitment or identification may occur continuously, or at a fixed point in time, and may involve approaching or identifying all participants (complete enumeration), or a sample.

Reporting the mechanisms for collecting individual level observations, including who was involved in identifying or recruiting participants, and whether they were blinded to the cluster's treatment allocation in a particular period, allows assessment of the likelihood of selection bias arising from selective identification or recruitment of participants. In trials where participants are identified or recruited prior to randomisation, there is no potential for selection bias (since selection of participants cannot be influenced by the cluster's treatment allocation, which is unknown prior to randomisation). However, more commonly, participants are identified or recruited post randomisation. In this circumstance, identification or recruitment of participants by a person blind to the allocation can help mitigate selection bias. In trials where there is complete enumeration, there may be less scope for selective identification or recruitment of participants, even when the recruiter is not blinded to the cluster's allocation; however, blinding to allocation status is always advisable.

Do you agree with the proposed checklist item?

Agree

Agree – but some change to the wording required (please explain)

Disagree (please explain)

Don’t know

Do you agree with the *content* of the proposed explanation?

Agree

Agree – but some additional rationale could be provided (please explain)

Disagree (please explain)

Don’t know

Further comments:

Section/ Topic: Methods Randomisation – Implementation

Item 10c: Implementation

Standard CONSORT item:

Not included in original CONSORT statement.

CONSORT cluster extension:

From whom consent was sought (representatives of the cluster, or individual cluster members, or both), and whether consent was sought before or after randomisation.

CONSORT crossover randomised trial extension [version 16/07/2018]**:**

Not included in crossover CONSORT extension.

CONSORT stepped-wedge cluster randomised trials extension [BMJ accepted]**:**

Whether, from whom and when consent was sought and for what; whether this differed between treatment conditions.

Proposed checklist item for CRXO trial (stepped-wedge CONSORT item):

Whether, from whom and when consent was sought and for what; whether this differed between treatment conditions.

Proposed explanation: In cluster randomised designs, consent is complicated by the different levels at which the intervention can be delivered, and at which, data collection can occur. The stepped-wedge CONSORT statement extension provides a detailed exposition of this item, most of which is directly applicable to CRXO randomised trials. Therefore, the stepped-wedge explanation is presented here, but has been tailored to CRXO trials (e.g. with reference to 'stepped-wedge' replaced by 'CRXO') to assist interpretation.

Obtaining informed consent for participation, study interventions, and data collection procedures in clinical trials is an integral principle of research ethics and international human rights law (25, 26). The process by which consent was obtained can lead to biases (16). It is important to describe what consent was for (e.g. exposure to the intervention or use of data), whether consent was sought before or after randomisation, and whether the type of consent differed between intervention and control conditions.

In CRXO trials there can be cluster-level research participants (e.g., health-care practitioners) and individual-level research participants (e.g. patients) (27). It is therefore important to identify explicitly from whom consent was obtained in the study or to state that consent was not obtained. Furthermore, in most cluster trials someone provides access to the cluster; such individuals are often called "gatekeepers" or "cluster guardians" (28). Gatekeeper permission for trial participation is different to consent from cluster-level research participants, such as health providers, for their own participation in the study.

In cluster randomised trials in which the treatment is delivered at the level of the cluster, it may not be possible to obtain consent for exposure to the intervention or control condition as the intervention may be impossible to avoid; however, consent can still be taken for use of data. It is therefore important to clearly report what consent was for. If participants recruited to the control and intervention conditions are given different information when their consent is taken, this can lead to bias (29). The information provided about the objectives of the study can itself prompt participants to act differently. For example, participants enrolled in a study of an intervention to increase uptake of HIV screening, who are fully informed about the objectives of the study, might increase uptake of screening irrespective of allocation to the intervention condition. This is known as the Hawthorne effect (30). Reporting what information was provided to participants can allow readers to judge the risks of such biases. In a review of CRXO trials (2), 'who' provided consent for the individual level participants to receive the intervention was reported

in 60/83 trials (72%). In those trials where consent was sought from individual participants (or other person on their behalf), it was unclear in 14/30 (47%) trials whether the participant had knowledge of the intervention they would receive prior to consenting.

Sometimes a research ethics committee might deem it appropriate that the study proceed without the informed consent of research participants (i.e. a waiver of consent) or the research ethics committee may otherwise modify informed consent requirements (i.e. modification of consent). When a waiver or modification of consent has been granted by a research ethics committee, it should be reported and a justification given. It should be clear whose consent was waived and whether the waiver pertains to study participation, data collection, or both. Not all jurisdictions allow for a waiver or modification of consent. Information on data collection procedures in the trial, e.g., whether data are anonymous or pseudo-anonymous, and whether they were routinely collected, can provide clarity around ethical aspects of the trial. When appropriate it can be useful to include any participant consent forms in appendices, which will allow readers to infer precisely the information provided to participants.

Do you agree with the proposed checklist item?

Agree

Agree – but some change to the wording required (please explain)

Disagree (please explain)

Don’t know

Do you agree with the *content* of the proposed explanation?

Agree

Agree – but some additional rationale could be provided (please explain)

Disagree (please explain)

Don’t know

Further comments:

Section/ Topic: Methods Blinding

Item 11a: Blinding

Standard CONSORT item:

If done, who was blinded after assignment to interventions (for example, participants, care providers, those assessing outcomes) and how.

CONSORT cluster extension:

No modification suggested.

CONSORT crossover randomised trial extension [version 16/07/2018]**:**

No modification suggested.

CONSORT stepped-wedge cluster randomised trials extension [BMJ accepted]**:**

If done, who was blinded after assignment to sequences (for example, cluster level participants, individual level participants, those assessing outcomes) and how.

Proposed checklist item for CRXO trial (stepped-wedge CONSORT item):

If done, who was blinded after assignment to sequences (for example, cluster level participants, individual level participants, those assessing outcomes) and how.

Proposed explanation: Clear reporting of whether those involved in the delivery of the treatments, receipt of the treatments, outcome collection and adjudication, and data analysis, were blinded to the treatment allocation is important for assessing the likelihood of bias. Performance bias may arise when healthcare providers or individual level participants are aware of the treatment allocation, and this knowledge leads deviations from the intended treatment (e.g. use of co-interventions, increased or decreased compliance with the treatment) (31). Similarly, detection bias may arise when the outcome assessor or adjudicator is aware of the treatment allocation, and this leads to differential assessment of the outcome. Outcome assessors might be cluster participants (e.g. patients [e.g. pain], healthcare providers [e.g. knowledge, skills]), representatives of a cluster [e.g. team climate outcomes]); healthcare providers (e.g. clinical examination); trial personnel; or persons who are independent of delivery of the intervention. In many trials, there will be different types of outcome assessors; therefore, clearly reporting which outcomes were assessed by whom, and whether they were blind to the treatment allocation is necessary.

Parallel and stepped-wedge cluster randomised trials may be more likely to evaluate interventions delivered to cluster-level participants (e.g. educational interventions delivered to healthcare providers to improve practice) (23, 32, 33), and in this circumstance, it will generally be impossible to blind cluster-level participants. However, in CRXO trials, interventions delivered at the individual participant level are more likely, and this may provide more opportunity to employ blinding of cluster- and individual-level participants (3).

The reporting of whether individual-level and cluster-level participants were blinded to the treatment allocation was well reported in a review of CRXO trials (> 80%). This was not the case for outcome assessment, where the blinding status of the outcome assessor (when not an individual-level participant) was clear in only 45/69 (65%) of trials (2).

Do you agree with the proposed checklist item?

Agree

Agree – but some change to the wording required (please explain)

Disagree (please explain)

Don’t know

Do you agree with the *content* of the proposed explanation?

Agree

Agree – but some additional rationale could be provided (please explain)

Disagree (please explain)

Don’t know

Further comments:

Section/ Topic: Methods Blinding

Item 11b: Blinding

Standard CONSORT item:

If relevant, description of the similarity of interventions.

CONSORT cluster extension:

No modification suggested.

CONSORT crossover randomised trial extension [version 16/07/2018]**:**

No modification suggested.

CONSORT stepped-wedge cluster randomised trials extension [BMJ accepted]**:**

No modification suggested.

Proposed checklist item for CRXO trial (standard CONSORT item):

If relevant, description of the similarity of interventions.

Proposed explanation: Readers are referred to the CONSORT statement for examples and explanation (6).

Do you agree with the proposed checklist item?

Agree

Agree – but some change to the wording required (please explain)

Disagree (please explain)

Don't know

Does this item require an explanation beyond the referenced CONSORT statement?

Yes (please explain reasons)

No

Don't know

Further comments:

Section/ Topic: Methods Statistical methods

Item 12a: Statistical methods

Standard CONSORT item:

Statistical methods used to compare groups for primary and secondary outcomes.

CONSORT cluster extension:

How clustering was taken into account.

CONSORT crossover randomised trial extension [version 16/07/2018]**:**

Statistical methods used to compare groups for primary and secondary outcomes which are appropriate for crossover design (i.e. based on within participant comparison).

CONSORT stepped-wedge cluster randomised trials extension [BMJ accepted]**:**

Statistical methods used to compare treatment conditions for primary and secondary outcomes including how time effects, clustering and repeated measures were taken into account.

Proposed checklist item for CRXO trial (modified item):

Statistical methods used to compare treatment conditions for primary and secondary outcomes including how period effects, carryover (if possible), clustering and repeated measures were taken into account.

Proposed explanation: Primary reasons for reporting the statistical methods are to allow replication and for the reader to assess whether the methods are appropriate for the design. To allow replication, the guiding principle should be to provide enough detail such that a knowledgeable individual with access to the original data could verify the reported results (34). Reference(s) to the chosen statistical methodology should be provided, along with details of the statistical software packages (and versions) used to implement the methods. The link between which statistical method(s) apply to which outcomes, should be made clear.

For parallel cluster randomised trials, it is well recognised that the statistical analysis must account for the correlation of observations within clusters (within period intra-cluster correlation (WP-ICC)). In CRXO trials, the correlation of observations within the same cluster, but in different periods (between period intra-cluster correlation for a 2x2 design (BP-ICC)), also needs to be accounted for, along with (where applicable) repeated measurements from the same participants. Analyses that do not allow for these aspects may yield incorrect estimates of treatment effect standard errors (and therefore confidence intervals with coverage that is less or greater than the nominal level (e.g. 95%)). Reporting the following is recommended (2):

- whether the analysis was performed at the individual or cluster level (by aggregating observations within each cluster-period into a single measure);
- how the cluster randomisation and multiple period aspects of the design were accounted for; and,
- when there were more than two periods, what assumptions were made about correlation of observations at different periods (or times).

In addition, if a period effect was considered, how this was accounted for in the analysis should be reported. Any tests for carryover effects, or adjustment for carryover, should be noted. Given CRXO trials typically include a small number of clusters (3), adjustment might be made for a set of prognostic factors at the participant level to mitigate any imbalance in participant characteristics between periods within a cluster, and this should be reported. Any methods for handling of missing data at the individual and cluster level should be described (e.g. (35)). Pre-specified analyses should be distinguished from those that were not planned.

Do you agree with the proposed checklist item?

Agree

Agree – but some change to the wording required (please explain)

Disagree (please explain)

Don’t know

Do you agree with the *content* of the proposed explanation?

Agree

Agree – but some additional rationale could be provided (please explain)

Disagree (please explain)

Don’t know

Further comments:

Section/ Topic: Methods Statistical methods

Item 12b: Statistical methods
Standard CONSORT item:
Methods for additional analyses, such as subgroup analyses and adjusted analyses.

CONSORT cluster extension:
No modification suggested.

CONSORT crossover randomised trial extension [version 16/07/2018]**:**
No modification suggested.

CONSORT stepped-wedge cluster randomised trials extension [BMJ accepted]**:**
No modification suggested.

Proposed checklist item for CRXO trial (standard CONSORT item):
Methods for additional analyses, such as subgroup analyses and adjusted analyses.

Proposed explanation: CRXO trials, like other designs, will commonly investigate subgroup differences and may perform adjusted analyses (6). In CRXO trials with a small number of clusters and where the primary analysis is an individual-level analysis (e.g. mixed-effects models, generalised estimating equations), there may be merit in undertaking a sensitivity analysis to compare the results with a cluster-level analysis, since individual-level analyses can have poor performance with few clusters (36). Details of such sensitivity analyses should be reported.

Do you agree with the proposed checklist item?

Agree

Agree – but some change to the wording required (please explain)

Disagree (please explain)

Don't know

Do you agree with the *content* of the proposed explanation?

Agree

Agree – but some additional rationale could be provided (please explain)

Disagree (please explain)

Don't know

Further comments:

Section/ Topic: Results: Participant flow (a diagram is strongly recommended)

Item 13a: Participant flow

Standard CONSORT item:

For each group, the numbers of participants who were randomly assigned, received intended treatment, and were analysed for the primary outcome.

CONSORT cluster extension:

For each group, the numbers of clusters that were randomly assigned, received intended treatment, and were analysed for the primary outcome.

CONSORT crossover randomised trial extension [version 16/07/2018]**:**

The numbers of participants who were randomly assigned, received intended treatment, and were analysed for the primary outcome, separately for each sequence and period.

CONSORT stepped-wedge cluster randomised trials extension [BMJ accepted]**:**

For each treatment condition or allocated sequence, the numbers of clusters and participants who were assessed for eligibility, were randomly assigned, received intended treatments and were analysed for the primary outcome (Figure 3).

Proposed checklist item for CRXO trial (modified item):

For each sequence and period, report a) the number of clusters assessed for eligibility, consented, and were randomly assigned, and b) the numbers of individuals who were assessed or identified, consented (if applicable), received the intended treatments and were analysed for the primary outcome.

Proposed explanation: Providing information on the flow of clusters and participants at each stage of a randomised trial allows assessment of generalisability and the likelihood of selection and attrition bias. A flow diagram provides a succinct visual depiction of this information, which is particularly valuable for more complex trials (6, 37). The flow diagram should allow assessment of any differential inclusion and attrition by the allocated sequence, treatment condition, and period. The exact form and content of the diagram will vary depending on the CRXO trial's specific design features, such as the number of sequences and whether the same people, different people, or a mixture, are assessed in different periods. For example, where the same participants contribute repeated measurements, they may provide these under all treatment conditions. In this circumstance, summarising the number of participants by the allocated sequence, along with the average number of measurements contributed by each participant, may be most appropriate (10).

Inclusion of a diagram showing the flow of participants through a trial could be improved. In a review of 469 randomised trials, only 263 (56%) included a CONSORT flow diagram. In addition, information was often missing from these diagrams (37).

Do you agree with the proposed checklist item?

Agree

Agree – but some change to the wording required (please explain)

Disagree (please explain)

Don't know

Do you agree with the *content* of the proposed explanation?

Agree

Agree – but some additional rationale could be provided (please explain)

Disagree (please explain)

Don’t know

Further comments:

Section/ Topic: Results: Participant flow (a diagram is strongly recommended)

Item 13b: Participant flow
Standard CONSORT item:
For each group, losses and exclusions after randomisation, together with reasons

CONSORT cluster extension:
For each group, losses and exclusions for both clusters and individual cluster members.

CONSORT crossover randomised trial extension [version 16/07/2018]**:**
Number of participants excluded at each stage, with reasons, separately for each sequence and period.

CONSORT stepped-wedge cluster randomised trials extension [BMJ accepted]**:**
For each treatment condition or allocated sequence, losses and exclusions for both clusters and participants with reasons.

Proposed checklist item for CRXO trial (modified item):
For each sequence and period, losses and exclusions for both clusters and participants with reasons.

Proposed explanation: Same as 13a.

Do you agree with the proposed checklist item?

Agree

Agree – but some change to the wording required (please explain)

Disagree (please explain)

Don't know

Do you agree with the *content* of the proposed explanation?

Agree

Agree – but some additional rationale could be provided (please explain)

Disagree (please explain)

Don't know

Further comments:

Section/ Topic: Results: Recruitment

Item 14a: Recruitment

Standard CONSORT item:

Dates defining the periods of recruitment and follow-up.

CONSORT cluster extension:

No modification suggested.

CONSORT crossover randomised trial extension [version 16/07/2018]**:**

No modification suggested.

CONSORT stepped-wedge cluster randomised trials extension [BMJ accepted]**:**

Dates defining the steps, initiation of intervention and deviations from planned dates. Dates defining recruitment and follow-up for participants.

Proposed checklist item for CRXO trial (modified item):

Dates of treatment periods, washout periods, recruitment and follow-up of participants.

Proposed explanation: Providing the dates of the treatment periods, washout periods and the recruitment and follow-up of participants is important for providing historical context for the trial (in particular, the treatments evaluated) (6), assessing the potential for carryover effects, and for potentially explaining the observed treatment effects. Providing the dates of the washout periods allows assessment of whether the length was sufficient to mitigate any carryover effects. This assessment will be informed by the dates when participants were recruited and followed-up. For example, in CRXO trials that plan to recruit different participants in each period, knowledge of the recruitment and follow-up dates allows assessment of whether some of the participants may have ended up receiving an alternate treatment.

Knowledge of the length of the treatment periods may help explain the treatment effects. For interventions that are not expected to have an immediate effect, periods will need to be of sufficient length to observe any impact of the intervention. Again, this assessment will be informed by participant recruitment and follow-up dates; in particular, whether participants are recruited immediately or there is a lag time before recruitment commences.

Do you agree with the proposed checklist item?

Agree

Agree – but some change to the wording required (please explain)

Disagree (please explain)

Don’t know

Do you agree with the *content* of the proposed explanation?

Agree

Agree – but some additional rationale could be provided (please explain)

Disagree (please explain)

Don’t know

Further comments:

Section/ Topic: Results: Recruitment

Item 14b: Recruitment

Standard CONSORT item:
Why the trial ended or was stopped.

CONSORT cluster extension:
No modification suggested.

CONSORT crossover randomised trial extension [version 16/07/2018]**:**
No modification suggested.

CONSORT stepped-wedge cluster randomised trials extension [BMJ accepted]**:**
No modification suggested.

Proposed checklist item for CRXO trial (standard CONSORT item):
Why the trial ended or was stopped.

Proposed explanation: Readers are referred to the CONSORT statement for examples and explanation (6).

Do you agree with the proposed checklist item?

Agree

Agree – but some change to the wording required (please explain)

Disagree (please explain)

Don’t know

Does this item require an explanation beyond the referenced CONSORT statement?

Yes (please explain reasons)

No

Don’t know

Further comments:

Section/ Topic: Results: Baseline data

Item 15: Baseline data

Standard CONSORT:

A table showing baseline demographic and clinical characteristics for each group.

CONSORT cluster extension:

Baseline characteristics for the individual and cluster levels as applicable for each group.

CONSORT crossover randomised trial extension [version 16/07/2018]**:**

A table showing baseline demographic and clinical characteristics for each group by sequence and period.

CONSORT stepped-wedge cluster randomised trials extension [BMJ accepted]**:**

Baseline characteristics for the individual and cluster levels as applicable for each treatment condition or allocated sequence.

Proposed checklist item for CRXO trial (modified item):

For each sequence and period, a table showing baseline characteristics for the cluster and individual level characteristics.

Proposed explanation: Reporting of baseline characteristics provides information on the characteristics of the clusters and individual-level participants that were actually recruited to the trial, which may differ to the eligibility criteria (e.g. if only urban emergency departments (EDs) agree to participate in a trial that was open to all EDs). This is necessary information for generalising the findings (item 21). In addition, reporting of baseline characteristics permits assessment of the success of the randomisation process, and in trials with post-randomisation recruitment of participants, can allow assessment of potential selection bias.

In multiple-period trials, the concept of 'baseline' is more complex because of the longitudinal nature of the design (10). Here, the CONSORT stepped-wedge terminology is adopted where a 'baseline' characteristic is considered one that is measured before exposure to the treatment condition or one that is not expected to be influenced by the treatment (e.g. age). Therefore, 'baseline' characteristics may be measured post-randomisation of the clusters, such as in CRXO trials where different participants are recruited in each period. In trials where repeated measurements are taken from the same participants, it may be possible to measure baseline characteristics prior to randomisation. Cluster level characteristics can usually be measured prior to randomisation and are often time-invariant.

Tabulating baseline characteristics for each sequence and period allows assessment of the success of randomisation, the potential for selection bias, and any systematic change in characteristics across the periods (2). In CRXO trials where the number of clusters is small, chance imbalance in cluster level characteristics between sequences may arise. The statistics reported should be descriptive (e.g. means and standard deviations), and not inferential (e.g. standard errors) (6).

In a review of CRXO trials, 24/83 (29%) did not present a table of baseline characteristics, seven (8%) reported baseline characteristics by sequence, a further five (6%) separated in some way by period, but none separated by sequence and period.

Do you agree with the proposed checklist item?

Agree

Agree – but some change to the wording required (please explain)

Disagree (please explain)

Don’t know

Do you agree with the *content* of the proposed explanation?

Agree

Agree – but some additional rationale could be provided (please explain)

Disagree (please explain)

Don’t know

Further comments:

Section/ Topic: Results: Numbers analysed

Item 16: Numbers analysed

Standard CONSORT:

For each group, number of participants (denominator) included in each analysis and whether the analysis was by original assigned groups.

CONSORT cluster extension:

For each group, number of clusters included in each analysis.

CONSORT crossover randomised trial extension [version 16/07/2018]**:**

For each group, number of participants (denominator) included in each analysis and whether the analysis was by original assigned groups.

CONSORT stepped-wedge cluster randomised trials extension [BMJ accepted]**:**

The number of observations and clusters included in each analysis for each treatment condition and whether the analysis was according to the allocated schedule.

Proposed checklist item for CRXO trial (modified item):

For each treatment condition, the number of clusters and observations in each analysis.

Proposed explanation: The number of clusters and observations by treatment condition should be reported for analyses of all outcomes. For the primary outcome(s), this information will be attainable from the flow chart of clusters and participants through the trial (item 13a). For analyses of secondary outcomes, reference may be made to the flow chart when the same number of clusters and participants are included. For outcomes where not all clusters of participants contribute to each analysis, in addition to providing the number of clusters and observations by treatment condition, it can be useful to provide the: number of clusters that contribute to the analysis across all periods (by sequence), the number that contribute to only some periods (by treatment and sequence), and the number of participants included in the analysis (by treatment and sequence). This information is most efficiently reported in a diagram.

The type of summary information may differ depending on whether the same people, different people, or a mixture, are assessed in different periods. For example, where the same participants contribute repeated measurements across the periods, they will have been exposed to both treatment conditions; therefore, the total number of observations can be presented by treatment condition, or as the number of participants in the study with the average number of observations per participant under each treatment condition (10).

Do you agree with the proposed checklist item?

Agree

Agree – but some change to the wording required (please explain)

Disagree (please explain)

Don’t know

Do you agree with the *content* of the proposed explanation?

Agree

Agree – but some additional rationale could be provided (please explain)

Disagree (please explain)

Don’t know

Further comments:

Section/ Topic: Results: Outcomes and estimation

Item 17a: Outcomes and estimation

Standard CONSORT item:

For each primary and secondary outcome, results for each group, and the estimated effect size and its precision (such as 95% confidence interval).

CONSORT cluster extension:

Results at the individual or cluster level as applicable and a coefficient of intra-cluster correlation (ICC or k) for each primary outcome.

CONSORT crossover randomised trial extension [version 16/07/2018]**:**

For each primary and secondary outcome, results, including estimated effect size and its precision (such as 95% confidence interval) should be based on within participant comparisons. In addition, results for each intervention in each period are recommended.

CONSORT stepped-wedge cluster randomised trials extension [BMJ accepted]**:**

For each primary and secondary outcome, results for each treatment condition, and the estimated effect size and its precision (such as 95% confidence interval); any correlations and time effects estimated in the analysis.

Proposed checklist item for CRXO trial (modified item):

For each primary and secondary outcome, summary statistics for each treatment condition and the estimated effect size and its precision (95% confidence interval); and any correlations estimated in the analysis. In addition, summary statistics for each treatment and period could be presented.

Proposed explanation: For each primary and secondary outcome, the estimated treatment effects with 95% confidence intervals should be reported. Reporting the summary statistics by treatment condition provides necessary information for assessing the severity or prevalence of the outcome in the trial participants, and the potential for any ceiling or floor effects. Separation of the summary statistics by period can allow assessment of any period effects or treatment by period interactions, and therefore judgement of the appropriateness of the analysis. P values may be provided, but these should be in addition to effect estimates and confidence intervals, and should be reported as exact values (i.e. not based on a significance threshold, for example, $P = 0.032$, not $P < 0.05$). The completeness of reporting of outcomes has been shown to be associated with statistical significance, where statistically significant outcomes are more often completely reported than non-significant outcomes, thus distorting the evidence base about the effects of treatments (38, 39).

Reporting of estimates of correlations arising from the CRXO design is critical for planning future trials (10, 16). For a CRXO trial with two periods, this will include estimates of the within-period intra-cluster correlation coefficient (ICC) and the between-period intra-cluster correlation coefficient (or cluster autocorrelation coefficient). With more than two periods the parameter estimates of the assumed within-cluster correlation structure over time should be reported. Given the number of possible correlations, explicit reporting of the type of correlation alongside the estimate is necessary. Variance components may be reported as an alternative to ICCs, particularly for non-continuous outcomes (40). When reporting ICCs for binary outcomes, indicating the scale (e.g. proportions or logistic scale) is necessary to know whether the ICC can be used in variance inflation formula (41).

Do you agree with the proposed checklist item?

Agree

Agree – but some change to the wording required (please explain)

Disagree (please explain)

Don’t know

Do you agree with the *content* of the proposed explanation?

Agree

Agree – but some additional rationale could be provided (please explain)

Disagree (please explain)

Don’t know

Further comments:

Section/ Topic: Results: Outcomes and estimation

Item 17b: Outcomes and estimation

Standard CONSORT item:

For binary outcomes, presentation of both absolute and relative effect sizes is recommended.

CONSORT cluster extension:

No modification suggested.

CONSORT crossover randomised trial extension [version 16/07/2018]**:**

No modification suggested.

CONSORT stepped-wedge cluster randomised trials extension [BMJ accepted]**:**

No modification suggested.

Proposed checklist item for CRXO trial (standard CONSORT item):

For binary outcomes, presentation of both absolute and relative effect sizes is recommended.

Proposed explanation: Readers are referred to the CONSORT statement for examples and explanation (6).

Consideration: An explanation could include:

- Further methodological work being required to determine optimal methods of analysis that yield an absolute measure of effect.
- Current approaches to estimating an absolute effect include fitting two separate models (for example a binomial model with log link to report the relative risks; and a binomial model with an identity link to report a risk difference) or by fitting one model and using a transformation to report the other measure of treatment effect.

Do you agree with the proposed checklist item?

Agree

Agree – but some change to the wording required (please explain)

Disagree (please explain)

Don't know

Does this item require an explanation beyond the referenced CONSORT statement (for example, by addressing points in the *Consideration* above)?

Yes (please explain reasons)

No

Don't know

Further comments:

Section/ Topic: Results: Ancillary analyses

Item 18: Ancillary analyses

Standard CONSORT item:

Item 18. Results of any other analyses performed, including subgroup analyses and adjusted analyses, distinguishing pre-specified from exploratory.

CONSORT cluster extension:

No modification suggested.

CONSORT crossover randomised trial extension [version 16/07/2018]**:**

No modification suggested.

CONSORT stepped-wedge cluster randomised trials extension [BMJ accepted]**:**

No modification suggested.

Proposed checklist item for CRXO trial (standard CONSORT item):

Results of any other analyses performed, including subgroup analyses and adjusted analyses, distinguishing pre-specified from exploratory.

Proposed explanation: Readers are referred to the CONSORT statement for examples and explanation (6).

Do you agree with the proposed checklist item?

Agree

Agree – but some change to the wording required (please explain)

Disagree (please explain)

Don't know

Does this item require an explanation beyond the referenced CONSORT statement?

Yes (please explain reasons)

No

Don't know

Further comments:

Section/ Topic: Results: Harms

Item 19: Harms

Standard CONSORT item:

All important harms or unintended effects in each group (for specific guidance see CONSORT for harms).

CONSORT cluster extension:

No modification suggested.

CONSORT crossover randomised trial extension [version 16/07/2018]**:**

Describe all important harms or untended effects in a way that accounts for the design (for specific guidance see CONSORT for harms (Ioannidis Ann Intern Med 2004)).

CONSORT stepped-wedge cluster randomised trials extension [BMJ accepted]**:**

Important harms or unintended effects in each treatment condition (for specific guidance see CONSORT for harms).

Proposed checklist item for CRXO trial (stepped-wedge CONSORT item):

Important harms or unintended effects in each treatment condition (for specific guidance see CONSORT for harms).

Proposed explanation: Readers are referred to the CONSORT statement and the extension to the CONSORT statement for examples and explanation (6, 42).

Do you agree with the proposed checklist item?

Agree

Agree – but some change to the wording required (please explain)

Disagree (please explain)

Don't know

Does this item require an explanation beyond the referenced CONSORT statements?

Yes (please explain reasons)

No

Don't know

Further comments:

Section/ Topic: Discussion: Limitations

Item 20: Limitations

Standard CONSORT item:

Trial limitations, addressing sources of potential bias, imprecision, and, if relevant, multiplicity of analyses.

CONSORT cluster extension:

No modification suggested.

CONSORT crossover randomised trial extension [version 16/07/2018]**:**

Trial limitations, addressing sources of potential bias, imprecision, and, if relevant, multiplicity of analyses. Consider potential carry-over effects.

CONSORT stepped-wedge cluster randomised trials extension [BMJ accepted]**:**

No modification suggested.

Proposed checklist item for CRXO trial (crossover CONSORT item):

Trial limitations, addressing sources of potential bias, imprecision, and, if relevant, multiplicity of analyses. Consider potential carry-over effects.

Proposed explanation: Reporting of the limitations is important since this allows readers to make an assessment of the potential for bias in the results. As the CRXO trial is a combination of the parallel group cluster randomised and crossover designs, it inherits the potential limitations of both designs. The principle limitation of a crossover design is the potential for carryover; whereby the effects of the treatment in one period persist into a subsequent period, thus potentially biasing estimates of the treatment effects (43). In a review of 83 CRXO trials, only 17 (20%) discussed the possibility of carryover (2).

The principle limitation of cluster trials is the potential for selection bias in which different types of participants are recruited (or identified) to the treatment groups, which can occur when those responsible for recruiting participants are aware of the cluster's group allocation. This may lead to biased estimates of the treatment effects.

Another possible limitation in a CRXO trial is when there are losses of clusters, or individuals, or both, between periods. Readers are referred to the CONSORT statement for discussion of imprecision and multiplicity of results (6).

Do you agree with the proposed checklist item?

Agree

Agree – but some change to the wording required (please explain)

Disagree (please explain)

Don't know

Do you agree with the *content* of the proposed explanation?

Agree

Agree – but some additional rationale could be provided (please explain)

Disagree (please explain)

Don’t know

Further comments:

Section/ Topic: Discussion:

Item 21: Discussion

Standard CONSORT item:

Generalisability (external validity, applicability) of the trial findings.

CONSORT cluster extension:

Generalisability to clusters and/or individual participants (as relevant)

CONSORT crossover randomised trial extension [version 16/07/2018]**:**

No modification suggested.

CONSORT stepped-wedge cluster randomised trials extension [BMJ accepted]**:**

Generalisability (external validity, applicability) of the trial findings. Generalisability to clusters and/or individual participants (as relevant).

Proposed checklist item for CRXO trial (stepped-wedge CONSORT item):

Generalisability (external validity, applicability) of the trial findings. Generalisability to clusters and/or individual participants (as relevant).

Proposed explanation: Readers are referred to the CONSORT statement and the extension to the CONSORT statement for examples and explanation (6, 16).

Do you agree with the proposed checklist item?

Agree

Agree – but some change to the wording required (please explain)

Disagree (please explain)

Don't know

Does this item require an explanation beyond the referenced CONSORT statements?

Yes (please explain reasons)

No

Don't know

Further comments:

Section/ Topic: Discussion: Interpretation

Item 22: Interpretation

Standard CONSORT item:

Interpretation consistent with results, balancing benefits and harms, and considering other relevant evidence.

CONSORT cluster extension:

No modification suggested

CONSORT crossover randomised trial extension [version 16/07/2018]**:**

No modification suggested.

CONSORT stepped-wedge cluster randomised trials extension [BMJ accepted]**:**

No modification suggested.

Proposed checklist item for CRXO trial (standard CONSORT item):   *Interpretation consistent with results, balancing benefits and harms, and considering other relevant evidence.* <br>***Proposed explanation:*** Readers are referred to the CONSORT statement for examples and explanation (6).

Do you agree with the proposed checklist item?

Agree

Agree – but some change to the wording required (please explain)

Disagree (please explain)

Don't know

Does this item require an explanation beyond the referenced CONSORT statement?

Yes (please explain reasons)

No

Don't know

Further comments:

Section/ Topic: Other information

Item 23: Trial registration

Standard CONSORT item:
Registration number and name of trial registry.

CONSORT cluster extension:
No modification suggested.

CONSORT crossover randomised trial extension [version 16/07/2018]**:**
No modification suggested.

CONSORT stepped-wedge cluster randomised trials extension [BMJ accepted]**:**
No modification suggested.

Proposed checklist item for CRXO trial (standard CONSORT item):
Registration number and name of trial registry.

Proposed explanation: Non-publication of trials, or non-reporting of outcomes or results within trials, distorts the evidence base on which clinical decisions are made about the benefits and harms of treatments (44-46). Reporting of trial registration details (registry name and unique registration number) allows assessment of important changes to the trial design, and the potential for any bias arising from missing outcomes or results. Provision of trial registration details also allows linking of multiple publications from the same trial, reducing the potential for a trial to inadvertently be included more than once in a systematic review. If the trial has not been registered, this should be reported along with the reason.

A growing number of studies have examined trial registration rates, reporting of registration details, and the timing of registration (e.g. (47-54)). Many of these studies indicate a large percentage of trials are not registered, and in those that are registered, not all report the registration details in the trial report, and many are retrospectively registered.

Do you agree with the proposed checklist item?

Agree

Agree – but some change to the wording required (please explain)

Disagree (please explain)

Don't know

Do you agree with the *content* of the proposed explanation?

Agree

Agree – but some additional rationale could be provided (please explain)

Disagree (please explain)

Don't know

Further comments:

Section/ Topic: Other information

Item 24: Trial protocol

Standard CONSORT item:

Where the full trial protocol can be accessed, if available.

CONSORT cluster extension:

No modification suggested

CONSORT crossover randomised trial extension [version 16/07/2018]**:**

No modification suggested.

CONSORT stepped-wedge cluster randomised trials extension [BMJ accepted]**:**

No modification suggested.

Proposed checklist item for CRXO trial (standard CONSORT item):

Where the full trial protocol can be accessed, if available.

Proposed explanation: Readers are referred to the CONSORT statement for examples and explanation (6).

Do you agree with the proposed checklist item?

Agree

Agree – but some change to the wording required (please explain)

Disagree (please explain)

Don't know

Does this item require an explanation beyond the referenced CONSORT statement?

Yes (please explain reasons)

No

Don't know

Further comments:

Section/ Topic: Other information

Item 25: Funding

Standard CONSORT item:

Sources of funding and other support (such as supply of drugs), role of funders.

CONSORT cluster extension:

No modification suggested.

CONSORT crossover randomised trial extension [version 16/07/2018]**:**

No modification suggested.

CONSORT stepped-wedge cluster randomised trials extension [BMJ accepted]**:**

No modification suggested.

Proposed checklist item for CRXO trial (standard CONSORT item):

Sources of funding and other support (such as supply of drugs), role of funders.

Proposed explanation: Readers are referred to the CONSORT statement for examples and explanation (6).

Do you agree with the proposed checklist item?

Agree

Agree – but some change to the wording re (please explain)

Disagree (please explain)

Don't know

Does this item require an explanation beyond the referenced CONSORT statement?

Yes (please explain reasons)

No

Don't know

Further comments:

Section/ Topic: Other information

Item 26: Research Ethics Review
Standard CONSORT item:
Not included.

CONSORT cluster extension:
Not included

CONSORT crossover randomised trial extension [version 16/07/2018]**:**
Not included.

CONSORT stepped-wedge cluster randomised trials extension [BMJ accepted]**:**
Whether the study was approved by a research ethics committee, with identification of the review committee(s). Justification for any waiver or modification of informed consent requirements.

Proposed checklist item for CRXO trial (stepped-wedge CONSORT item):
Whether the study was approved by a research ethics committee, with identification of the review committee(s). Justification for any waiver or modification of informed consent requirements.

Proposed explanation: The International Committee of Medical Journal Editors (ICMJE) makes the recommendation that a statement should be made indicating that the research was approved by an independent local, regional, or national review body (ICMJE 2017). The original CONSORT statement did not include an item on research ethics approval. However, recent extensions to the CONSORT statement for pilot studies (55) and stepped-wedge trials (10) have introduced such an item. In the case of cluster randomised designs, there is evidence of under-reporting of ethics review, with 26% (77/300) of cluster randomised trials (56) and 25% (8/32) of stepped-wedge trials (33) failing to report review by a research ethics committee. The application or reference number should be reported in addition to the ethics committee(s). Any exemptions from review by a research ethics committee should be reported together with the justification for exemption.

Do you agree with the proposed checklist item?

Agree

Agree – but some change to the wording required (please explain)

Disagree (please explain)

Don't know

Do you agree with the *content* of the proposed explanation?

Agree

Agree – but some additional rationale could be provided (please explain)

Disagree (please explain)

Don't know

Further comments:

Additional items

Are there any additional reporting items that you think should be included in the CONSORT CRXO extension?

Yes (please list below, including justification)

No

Additional items

Survey references

1. Moher D, Schulz KF, Simera I, Altman DG. Guidance for Developers of Health Research Reporting Guidelines. PLoS Med. 2010;7:e1000217.
2. Arnup SJ, Forbes AB, Kahan BC, Morgan KE, McKenzie JE. The quality of reporting in cluster randomised crossover trials: proposal for reporting items and an assessment of reporting quality. Trials. 2016;17:575.
3. Arnup SJ, Forbes AB, Kahan BC, Morgan KE, McKenzie JE. Appropriate statistical methods were infrequently used in cluster-randomized crossover trials. J Clin Epidemiol. 2016;74:40-50.
4. Qualtrics. Copyright year. 2023.: Qualtrics. Provo, Utah, USA. https://www.qualtrics.com; 2005 [first release].
5. Hemming K, Taljaard M, McKenzie JE, et al. Reporting of stepped wedge cluster randomised trials: extension of the CONSORT 2010 statement with explanation and elaboration. BMJ. 2018;363:k1614.
6. Moher D, Hopewell S, Schulz KF, et al. CONSORT 2010 Explanation and Elaboration: updated guidelines for reporting parallel group randomised trials. BMJ. 2010;340:c869.
7. Bellomo R, Forbes A, Akram M, Bailey M, Pilcher DV, Cooper DJ. Why we must cluster and cross over. Crit Care Resusc. 2013;15:155-7.
8. Forbes AB, Akram M, Pilcher D, Cooper J, Bellomo R. Cluster randomised crossover trials with binary data and unbalanced cluster sizes: application to studies of near-universal interventions in intensive care. Clin Trials. 2015;12:34-44.
9. Giraudeau B, Ravaud P, Donner A. Sample size calculation for cluster randomized cross-over trials. Stat Med. 2008;27:5578-85.
10. Hemming K, Taljaard M, McKenzie JE, et al. The CONSORT extension for Stepped-Wedge Cluster Randomised Trials. Bmj. 2018;Accepted March 2018.
11. Senn S. The AB/BA Design with Normal Data. Cross-over Trials In Clinical Research: John Wiley & Sons, Ltd; 2003. p. 35-88.
12. Zwarenstein M, Treweek S, Gagnier JJ, et al. Improving the reporting of pragmatic trials: an extension of the CONSORT statement. BMJ. 2008;337:a2390.
13. Eldridge S, Ashby D, Bennett C, Wakelin M, Feder G. Internal and external validity of cluster randomised trials: systematic review of recent trials. BMJ. 2008;336:876-80.
14. Hoffmann TC, Glasziou PP, Boutron I, et al. Better reporting of interventions: template for intervention description and replication (TIDieR) checklist and guide. BMJ. 2014;348:g1687.
15. Welton NJ, Caldwell DM, Adamopoulos E, Vedhara K. Mixed treatment comparison meta-analysis of complex interventions: psychological interventions in coronary heart disease. American journal of epidemiology. 2009;169:1158-65.
16. Campbell MK, Piaggio G, Elbourne DR, Altman DG. Consort 2010 statement: extension to cluster randomised trials. BMJ. 2012;345:e5661.
17. Rutterford C, Taljaard M, Dixon S, Copas A, Eldridge S. Reporting and methodological quality of sample size calculations in cluster randomized trials could be improved: a review. J Clin Epidemiol. 2015;68:716-23.
18. Parienti JJ, Kuss O. Cluster-crossover design: a method for limiting clusters level effect in community-intervention studies. Contemporary clinical trials. 2007;28:316-23.
19. Hooper R, Teerenstra S, de Hoop E, Eldridge S. Sample size calculation for stepped wedge and other longitudinal cluster randomised trials. Stat Med. 2016.
20. Kasza J, Hemming K, Hooper R, Matthews J, Forbes AB. Impact of non-uniform correlation structure on sample size and power in multiple-period cluster randomised trials. Stat Methods Med Res. 2019;28:703-16.
21. Goodman SN, Berlin JA. The use of predicted confidence intervals when planning experiments and the misuse of power when interpreting results. Ann Intern Med. 1994;121:200-6.
22. Schulz KF, Grimes DA. Allocation concealment in randomised trials: defending against deciphering. Lancet. 2002;359:614-8.
23. Eldridge S, Campbell MK, Campbell MJ, et al. Revised Cochrane risk of bias tool for randomized trials (RoB 2) Additional considerations for cluster-randomized trials (RoB 2 CRT). https://www.riskofbias.info/welcome/rob-2-0-tool/rob-2-for-cluster-randomized-trials; 2021.

24. Caille A, Kerry S, Tavernier E, Leyrat C, Eldridge S, Giraudeau B. Timeline cluster: a graphical tool to identify risk of bias in cluster randomised trials. BMJ. 2016;354:i4291.
25. Capron AM. Where Did Informed Consent for Research Come From? The Journal of law, medicine & ethics : a journal of the American Society of Law, Medicine & Ethics. 2018;46:12-29.
26. United Nations. International Covenant on Civil and Political Rights. 1966.
27. Taljaard M, Weijer C, Grimshaw JM, Eccles MP, Ottawa Ethics of Cluster Randomised Trials Consensus G. The Ottawa Statement on the ethical design and conduct of cluster randomised trials: precis for researchers and research ethics committees. BMJ. 2013;346:f2838.
28. Edwards SJ, Braunholtz DA, Lilford RJ, Stevens AJ. Ethical issues in the design and conduct of cluster randomised controlled trials. BMJ. 1999;318:1407-9.
29. Eldridge SM, Ashby D, Feder GS. Informed patient consent to participation in cluster randomized trials: an empirical exploration of trials in primary care. Clin Trials. 2005;2:91-8.
30. McCarney R, Warner J, Iliffe S, van Haselen R, Griffin M, Fisher P. The Hawthorne Effect: a randomised, controlled trial. BMC Med Res Methodol. 2007;7:30.
31. Higgins JPT, Savović J, Page MJ, Sterne JAC, the development group for RoB 2.0. Revised Cochrane risk of bias tool for randomized trials (RoB 2.0). https://sites.google.com/site/riskofbiastool/; 2016.
32. Beard E, Lewis JJ, Copas A, et al. Stepped wedge randomised controlled trials: systematic review of studies published between 2010 and 2014. Trials. 2015;16:353.
33. Taljaard M, Hemming K, Shah L, Giraudeau B, Grimshaw JM, Weijer C. Inadequacy of ethical conduct and reporting of stepped wedge cluster randomized trials: Results from a systematic review. Clin Trials. 2017:1740774517703057.
34. ICMJE International Committee of Medical Journal Editors. Recommendations for the Conduct, Reporting, Editing and Publication of Scholarly Work in Medical Journals.;Updated January 2024. https://www.icmje.org/.
35. Taljaard M, Donner A, Klar N. Imputation strategies for missing continuous outcomes in cluster randomized trials. Biom J. 2008;50:329 - 45.
36. Morgan KE, Forbes AB, Keogh RH, Jairath V, Kahan BC. Choosing appropriate analysis methods for cluster randomised cross-over trials with a binary outcome. Stat Med. 2017;36:318-33.
37. Hopewell S, Hirst A, Collins GS, Mallett S, Yu LM, Altman DG. Reporting of participant flow diagrams in published reports of randomized trials. Trials. 2011;12:253.
38. Chan AW, Hrobjartsson A, Haahr MT, Gotzsche PC, Altman DG. Empirical evidence for selective reporting of outcomes in randomized trials: comparison of protocols to published articles. JAMA. 2004;291:2457-65.
39. Chan AW, Krleza-Jeric K, Schmid I, Altman DG. Outcome reporting bias in randomized trials funded by the Canadian Institutes of Health Research. CMAJ. 2004;171:735-40.
40. Hayes RJ, Bennett S. Simple sample size calculation for cluster-randomized trials. Int J Epidemiol. 1999;28:319-26.
41. Eldridge SM, Ukoumunne OC, Carlin JB. The intra-cluster correlation coefficient in cluster ranomized trials: A review of definitions. Int Stat Rev. 2009;77:378-94.
42. Ioannidis JP, Evans SJ, Gotzsche PC, et al. Better reporting of harms in randomized trials: an extension of the CONSORT statement. Ann Intern Med. 2004;141:781-8.
43. Edited by Higgins JPT, Li T, Sterne JA, on behalf of the RoB 2 working group on crossover trials. Revised Cochrane risk of bias tool for randomized trials (RoB 2) Additional considerations for crossover trials. https://www.riskofbias.info/welcome/rob-2-0-tool/rob-2-for-crossover-trials; 2021.
44. Dwan K, Altman DG, Clarke M, et al. Evidence for the Selective Reporting of Analyses and Discrepancies in Clinical Trials: A Systematic Review of Cohort Studies of Clinical Trials. PLoS Med. 2014;11:e1001666.
45. Dwan K, Gamble C, Williamson PR, Kirkham JJ, Reporting Bias G. Systematic review of the empirical evidence of study publication bias and outcome reporting bias - an updated review. PLoS One. 2013;8:e66844.
46. Song F, Parekh S, Hooper L, et al. Dissemination and publication of research findings: an updated review of related biases. Health technology assessment (Winchester, England). 2010;14:iii, ix-xi, 1-193.

47. Azar M, Riehm KE, McKay D, Thombs BD. Transparency of Outcome Reporting and Trial Registration of Randomized Controlled Trials Published in the <italic>Journal of Consulting and Clinical Psychology</italic>. PLoS ONE. 2015;10:e0142894.
48. Gray R, Badnapurkar A, Hassanein E, et al. Registration of randomized controlled trials in nursing journals. Research integrity and peer review. 2017;2:8.
49. Harriman SL, Patel J. When are clinical trials registered? An analysis of prospective versus retrospective registration. Trials. 2016;17:187.
50. Killeen SMDF, Sourallous PM, Hunter IAPF, Hartley JEMDBF, Grady HLOMDF. Registration Rates, Adequacy of Registration, and a Comparison of Registered and Published Primary Outcomes in Randomized Controlled Trials Published in Surgery Journals. Annals of Surgery. 2014;259:193-6.
51. Mathieu S, Boutron I, Moher D, Altman DG, Ravaud P. Comparison of registered and published primary outcomes in randomized controlled trials. JAMA. 2009;302:977-84.
52. Riehm KE, Azar M, Thombs BD. Transparency of outcome reporting and trial registration of randomized controlled trials in top psychosomatic and behavioral health journals: A 5-year follow-up. Journal of psychosomatic research. 2015;79:1-12.
53. Scott A, Rucklidge JJ, Mulder RT. Is Mandatory Prospective Trial Registration Working to Prevent Publication of Unregistered Trials and Selective Outcome Reporting? An Observational Study of Five Psychiatry Journals That Mandate Prospective Clinical Trial Registration. PLoS One. 2015;10:e0133718.
54. van de Wetering FT, Scholten RJPM, Haring T, Clarke M, Hooft L. Trial Registration Numbers Are Underreported in Biomedical Publications. PLOS ONE. 2012;7:e49599.
55. Eldridge SM, Chan CL, Campbell MJ, et al. CONSORT 2010 statement: extension to randomised pilot and feasibility trials. BMJ. 2016;355:i5239.
56. Taljaard M, McRae AD, Weijer C, et al. Inadequate reporting of research ethics review and informed consent in cluster randomised trials: review of random sample of published trials. BMJ. 2011;342:d2496.
