# Supplementary material for: Reporting of cluster randomised crossover trials: extension of the CONSORT 2010 statement with explanation and elaboration
Source: BMJ. 2025 Jan 6;388:e080472. doi: 10.1136/bmj-2024-080472 (PMC11701780; doi:10.1136/bmj-2024-080472)
Supplement: Supplementary file 2 — Web appendix 2: Supplementary material 2 [file mckj080472.ww2.pdf]

Supplementary material 2 for 'Reporting of cluster randomised crossover trials: extension of the CONSORT 2010 statement with explanation and elaboration'

McKenzie JE*, Taljaard M, Hemming K, Arnup SJ, Giraudeau B, Eldridge S, Hooper R, Kahan BC, Li T, Moher D, Grimshaw JM, Forbes AB. BMJ 2025;388:e080472. doi:10.1136/bmj-2024-080472

* Correspondence to: Professor Joanne McKenzie, School of Public Health and Preventive Medicine, Monash University, 553 St Kilda Road, Melbourne, Victoria 3004, Australia
Email: joanne.mckenzie@monash.edu

Table of contents:

Table S1. Comparison of items across the standard CONSORT 2010 statement and design extensions for CRXO, cluster, crossover and stepped-wedge*† 2

Table S2. Comparison of items to report in journal or conference abstracts (item 1b) across the standard CONSORT 2010 statement and design extensions for CRXO, cluster, crossover and stepped-wedge* 11

Table S3. Evidence of reporting quality* 14

References 20

Table S1. Comparison of items across the standard CONSORT 2010 statement and design extensions for CRXO, cluster, crossover and stepped-wedge*†

		CONSORT statement or extension				
**Section** / Topic	Item no.	**CRXO 2024**	**CONSORT 2010**	**Cluster 2012‡**	**Crossover 2019**	**Stepped-wedge 2018**
**Title and Abstract**						
	1a	Identification as a cluster randomised crossover trial in the title.	Identification as a randomised trial in the title	Identification as a cluster randomised trial in the title	Identification as a randomised crossover trial in the title	Identification as a SW-CRT in the title.
	1b	Structured summary of the trial design, methods, results, and conclusions (see separate CRXO checklist for abstracts)<br><br>[see Table S2 below for items]	Structured summary of trial design, methods, results, and conclusions (for specific guidance see CONSORT for abstracts)<br>[see Table S2 below for items]	Structured summary of trial design, methods, results, and conclusions See table 2 (for specific guidance see CONSORT for abstracts)<br>[see Table S2 below for items]	Specify a crossover design and report all information outlined in table 2<br><br>[see Table S2 below for items]	Structured summary of trial design, methods, results, and conclusions (see separate SW-CRT checklist for abstracts).<br><br>[see Table S2 below for items]
**Introduction**						
Background and objectives	2a	Scientific background and explanation of rationale.	Scientific background and explanation of rationale	Scientific background and explanation of rationale<br>*Extension:* Rationale for using a cluster design	Scientific background and explanation of rationale	Scientific background. Rationale for using a cluster design and rationale for using a stepped wedge design.
	2b	Specific objectives or hypotheses.	Specific objectives or hypotheses	Specific objectives or hypotheses<br>*Extension:* Whether objectives pertain to the cluster level, the individual participant level, or both	Specific objectives or hypotheses	Specific objectives or hypotheses.
**Methods**						
Trial design	3a.1<br><br>3a.2	Rationale for a cluster crossover design.<br>Description of the realised trial design including:<br>• number of treatment conditions;<br>• definition of cluster (i.e. the unit of randomisation);<br>• number and duration of periods;<br>• number and composition of sequences (e.g. ABAB, BABA);	3a. Description of trial design (such as parallel, factorial) including allocation ratio	3a. Description of trial design (such as parallel, factorial) including allocation ratio<br>*Extension:* Definition of cluster and description of how the design features apply to the clusters	3a. Rationale for a crossover design. Description of the design features including allocation ratio, especially the number and duration of periods, duration of washout period, and consideration of carry over effect	3a. Description and diagram of trial design including definition of cluster, number of sequences, number of clusters randomised to each sequence, number of periods, duration of time between each step, and whether the participants assessed in different periods are the same people, different people, or a mixture.

Section / Topic	Item no.	CRXO 2024	CONSORT 2010	Cluster 2012‡	Crossover 2019	Stepped-wedge 2018
		**CONSORT statement or extension**				
		• number of clusters randomised to each sequence;<br>• duration of any washout periods;<br>• whether the participants assessed in different periods are the same people, different people, or a mixture of the two; and<br>• consideration of potential for carryover effects.<br>A diagram of the trial is recommended when there are more than two periods and/or treatment conditions.				
	3b	Important changes to planned methods after trial commencement (such as eligibility criteria), with reasons.	Important changes to methods after trial commencement (such as eligibility criteria), with reasons	Important changes to methods after trial commencement (such as eligibility criteria), with reasons	Important changes to methods after trial commencement (such as eligibility criteria), with reasons	Important changes to methods after trial commencement (such as eligibility criteria), with reasons.
Participants	4a	Eligibility criteria for clusters and participants.	Eligibility criteria for participants	Eligibility criteria for participants<br>*Extension:* Eligibility criteria for clusters	Eligibility criteria for participants	Eligibility criteria for clusters and participants.
	4b	Settings and locations where the data were collected.	Settings and locations where the data were collected	Settings and locations where the data were collected	Settings and locations where the data were collected	Settings and locations where the data were collected.
Intervention	5	The treatment conditions with sufficient details to allow replication, and whether they were delivered at the level of the cluster, the individual, or both.	The interventions for each group with sufficient details to allow replication, including how and when they were actually administered	The interventions for each group with sufficient details to allow replication, including how and when they were actually administered<br>*Extension:* Whether interventions pertain to the cluster level, the individual participant level, or both	The interventions with sufficient details to allow replication, including how and when they were actually administered	The intervention and control conditions with sufficient details to allow replication, including whether the intervention was maintained or repeated, and whether it was delivered at the cluster level, the individual participant level, or both.
Outcomes	6a	Completely defined pre-specified primary and	Completely defined pre-specified primary and	Completely defined prespecified primary and	Completely defined prespecified primary and	Completely defined prespecified primary and

Section / Topic	Item no.	CONSORT statement or extension: CRXO 2024	CONSORT 2010	Cluster 2012‡	Crossover 2019	Stepped-wedge 2018
		secondary outcome measures, including how and when they were assessed (for specific guidance see CONSORT for outcomes).	secondary outcome measures, including how and when they were assessed	secondary outcome measures, including how and when they were assessed<br>*Extension:* Whether outcome measures pertain to the cluster level, the individual participant level, or both	secondary outcome measures, including how and when they were assessed	secondary outcome measures, including how and when they were assessed.
	6b	Any changes to trial outcomes after the trial commenced, with reasons.	Any changes to trial outcomes after the trial commenced, with reasons	Any changes to trial outcomes after the trial commenced, with reasons	Any changes to trial outcomes after the trial commenced, with reasons	Any changes to trial outcomes after the trial commenced, with reasons.
Sample size	7a	How sample size was determined. Method of calculation and relevant parameters with sufficient detail so the calculation can be reproduced. Assumptions made about correlations between outcomes of participants from the same cluster (see separate CRXO checklist for sample size items)	How sample size was determined	How sample size was determined<br>*Extension:* Method of calculation, number of clusters(s) (and whether equal or unequal cluster sizes are assumed), cluster size, a coefficient of intracluster correlation (ICC or k), and an indication of its uncertainty	How sample size was determined, accounting for within participant variability	How sample size was determined. Method of calculation and relevant parameters with sufficient detail so the calculation can be replicated. Assumptions made about correlations between outcomes of participants from the same cluster. (see separate checklist for SW-CRT sample size items).
	7b	When applicable, explanation of any interim analyses and stopping guidelines.	When applicable, explanation of any interim analyses and stopping guidelines	When applicable, explanation of any interim analyses and stopping guidelines	When applicable, explanation of any interim analyses and stopping guidelines	When applicable, explanation of any interim analyses and stopping guidelines.
Randomisation:						
Schedule generation¶	8a	Method used to generate the random allocation schedule.	Method used to generate the random allocation sequence	Method used to generate the random allocation sequence	Method used to generate the random allocation sequence	Method used to generate the random allocation to the sequences of treatments.
	8b	Type of randomisation; details of any restricted randomisation, if used.	Type of randomisation; details of any restriction (such as blocking and block size)	Type of randomisation; details of any restriction (such as blocking<br>*Extension:* Details of stratification or matching if used and block size)	Type of randomisation; details of any restriction (such as blocking and block size)	Type of randomisation; details of any constrained randomisation or stratification, if used.
Allocation concealment mechanism	9	Specification that allocation was based on clusters; description of any methods used to conceal the allocation	Mechanism used to implement the random allocation sequence (such as sequentially numbered containers), describing any steps taken to	Mechanism used to implement the random allocation sequence (such as sequentially numbered containers), describing any steps taken to	Mechanism used to implement the random allocation sequence (such as sequentially numbered containers), describing any steps taken to	Specification that allocation was based on clusters; description of any methods used to conceal the allocation

Section / Topic	Item no.	CRXO 2024	CONSORT 2010	Cluster 2012‡	Crossover 2019	Stepped-wedge 2018
		**CONSORT statement or extension**				
		from the clusters until after their recruitment.	conceal the sequence until interventions were assigned	conceal the sequence until interventions were assigned *Extension:* Specification that allocation was based on clusters rather than individuals and whether allocation concealment (if any) was at the cluster level, the individual participant level, or both	conceal the sequence until interventions were assigned	from the clusters until after recruitment.
Implementation	10a	Who generated the random allocation schedule, who enrolled clusters, and who assigned clusters to sequences of treatments in the schedule.	10. Who generated the random allocation sequence, who enrolled participants, and who assigned participants to interventions	Who generated the random allocation sequence, who enrolled clusters, and who assigned clusters to interventions	10. Who generated the random allocation sequence, who enrolled participants, and who assigned participants to the sequence of interventions	Who generated the randomisation schedule, who enrolled clusters, and who assigned clusters to sequences.
	10b	Mechanism by which individual participants were included in clusters for the purposes of the trial (such as complete enumeration or random sampling; continuous recruitment or ascertainment; or recruitment at a fixed point in time), including who recruited or identified participants.		Mechanism by which individual participants were included in clusters for the purposes of the trial (such as complete enumeration, random sampling)		Mechanism by which individual participants were included in clusters for the purposes of the trial (such as complete enumeration or random sampling; continuous recruitment or ascertainment; or recruitment at a fixed point in time), including who recruited or identified participants.
	10c	Whether consent was sought, from whom, when and for what; whether this differed between treatment conditions. Justification for any waiver or modification of informed consent requirements.		From whom consent was sought (representatives of the cluster, or individual cluster members, or both) and whether consent was sought before or after randomisation		Whether, from whom and when consent was sought and for what; whether this differed between treatment conditions.
Blinding	11a	Who was blinded after assignment to sequences (e.g. cluster level participants, individual level participants, those assessing outcomes) and how.	If done, who was blinded after assignment to interventions (for example, participants, care providers, those assessing outcomes) and how	If done, who was blinded after assignment to interventions (for example, participants, care providers, those assessing outcomes) and how	If done, who was blinded after assignment to interventions (for example, participants, care providers, those assessing outcomes) and how	If done, who was blinded after assignment to sequences (eg, cluster level participants, individual level participants, those assessing outcomes) and how.

Section / Topic	Item no.	CRXO 2024	CONSORT 2010	Cluster 2012‡	Crossover 2019	Stepped-wedge 2018
		**CONSORT statement or extension**				
	11b	If relevant, description of the similarity of interventions.	If relevant, description of the similarity of interventions	If relevant, description of the similarity of interventions	If relevant, description of the similarity of interventions	If relevant, description of the similarity of treatments.
Statistical methods	12a	Target estimand for each primary and secondary outcome including whether it pertains to the cluster-level or individual-level; statistical methods for their estimation including how period effects, clustering and repeated measures were taken into account. Any assessment of carryover effects should be reported.	Statistical methods used to compare groups for primary and secondary outcomes	Statistical methods used to compare groups for primary and secondary outcomes<br>*Extension:* How clustering was taken into account	Statistical methods used to compare groups for primary and secondary outcomes which are appropriate for crossover design (that is, based on within participant comparison)	Statistical methods used to compare treatment conditions for primary and secondary outcomes including how time effects, clustering and repeated measures were taken into account.
	12b	Methods for additional analyses, such as subgroup analyses, sensitivity analyses, and adjusted analyses.	Methods for additional analyses, such as subgroup analyses and adjusted analyses	Methods for additional analyses, such as subgroup analyses and adjusted analyses	Methods for additional analyses, such as subgroup analyses and adjusted analyses	Methods for additional analyses, such as subgroup analyses, sensitivity analyses, and adjusted analyses.
**Results**						
Participant flow (a diagram is strongly recommended)	13a	The numbers of clusters that were assessed for eligibility and were randomly assigned to each sequence. For each sequence-period (i.e. each cell) or treatment condition:<br>• the numbers of clusters that received intended treatments and were analysed for the primary outcome; and,<br>• the numbers of participants who were assessed for eligibility, received intended treatments and were analysed for the primary outcome.	For each group, the numbers of participants who were randomly assigned, received intended treatment, and were analysed for the primary outcome	For each group, the numbers of participants who were randomly assigned, received intended treatment, and were analysed for the primary outcome<br>*Extension:* For each group, the numbers of clusters that were randomly assigned, received intended treatment, and were analysed for the primary outcome	The numbers of participants who were randomly assigned, received intended treatment, and were analysed for the primary outcome, separately for each sequence and period	For each treatment condition or allocated sequence, the numbers of clusters and participants who were assessed for eligibility, were randomly assigned, received intended treatments, and were analysed for the primary outcome (see separate SW-CRT flow chart).

Section / Topic	Item no.	CONSORT statement or extension: CRXO 2024	CONSORT 2010	Cluster 2012‡	Crossover 2019	Stepped-wedge 2018
	13b	For each sequence-period (i.e. each cell) or treatment condition, losses and exclusions for both clusters and participants with reasons.	For each group, losses and exclusions after randomisation, together with reasons	For each group, losses and exclusions after randomisation, together with reasons<br>*Extension:* For each group, losses and exclusions for both clusters and individual cluster members	No of participants excluded at each stage, with reasons, separately for each sequence and period	For each treatment condition or allocated sequence, losses and exclusions for both clusters and participants with reasons.
Recruitment	14a	Dates of treatment periods and washout periods.	Dates defining the periods of recruitment and follow-up	Dates defining the periods of recruitment and follow-up	Dates defining the periods of recruitment and follow-up	Dates defining the steps, initiation of intervention, and deviations from planned dates. Dates defining recruitment and follow-up for participants.
	14b	Why the trial ended or was stopped.	Why the trial ended or was stopped	Why the trial ended or was stopped	Why the trial ended or was stopped	Why the trial ended or was stopped.
Baseline data	15	A table showing baseline cluster level characteristics by sequence, and individual level characteristics for each sequence-period (i.e. each cell) or treatment condition.	A table showing baseline demographic and clinical characteristics for each group	A table showing baseline demographic and clinical characteristics for each group<br>*Extension:* Baseline characteristics for the individual and cluster levels as applicable for each group	A table showing baseline demographic and clinical characteristics by sequence and period	Baseline characteristics for the individual and cluster levels as applicable for each treatment condition or allocated sequence.
Numbers analysed	16	The number of observations and clusters included in each analysis for each treatment condition and whether the analysis was according to the allocated schedule.	For each group, number of participants (denominator) included in each analysis and whether the analysis was by original assigned groups	For each group, number of participants (denominator) included in each analysis and whether the analysis was by original assigned groups<br>*Extension:* For each group, number of clusters included in each analysis	Number of participants (denominator) included in each analysis and whether the analysis was by original assigned groups	The number of observations and clusters included in each analysis for each treatment condition and whether the analysis was according to the allocated schedule.
Outcomes and estimation	17a	For each primary and secondary outcome, summary statistics by sequence-period (i.e. each cell) or treatment condition; the estimated effect size and its precision (e.g. 95% confidence interval); and any within cluster correlations or variance components estimated in the analysis.	For each primary and secondary outcome, results for each group, and the estimated effect size and its precision (such as 95% confidence interval)	For each primary and secondary outcome, results for each group, and the estimated effect size and its precision (such as 95% confidence interval)<br>*Extension:* Results at the individual or cluster level as applicable and a coefficient of	For each primary and secondary outcome, results including estimated effect size and its precision (such as 95% confidence interval) should be based on within participant comparisons. In addition, results for each intervention in each period are recommended	For each primary and secondary outcome, results for each treatment condition, and the estimated effect size and its precision (such as 95% confidence interval); any correlations (or covariances) and time effects estimated in the analysis.

Section / Topic	Item no.	CONSORT statement or extension<br>CRXO 2024	CONSORT 2010	Cluster 2012‡	Crossover 2019	Stepped-wedge 2018
				intracluster correlation (ICC or k) for each primary outcome		
	17b	For binary outcomes, presentation of both absolute and relative effect sizes is recommended.	For binary outcomes, presentation of both absolute and relative effect sizes is recommended	For binary outcomes, presentation of both absolute and relative effect sizes is recommended	For binary outcomes, presentation of both absolute and relative effect sizes is recommended	For binary outcomes, presentation of both absolute and relative effect sizes is recommended.
Ancillary analyses	18	Results of any other analyses performed, including subgroup analyses, sensitivity analyses, and adjusted analyses, distinguishing pre-specified from exploratory.	Results of any other analyses performed, including subgroup analyses and adjusted analyses, distinguishing pre-specified from exploratory	Results of any other analyses performed, including subgroup analyses and adjusted analyses, distinguishing prespecified from exploratory	Results of any other analyses performed, including subgroup analyses and adjusted analyses, distinguishing prespecified from exploratory	Results of any other analyses performed, including subgroup analyses and adjusted analyses, distinguishing prespecified from exploratory.
Harms	19	Important harms or unintended effects in each treatment condition (for specific guidance see CONSORT Harms 2022 statement).	All important harms or unintended effects in each group (for specific guidance see CONSORT for harms)	All important harms or unintended effects in each group (for specific guidance see CONSORT for harms)	Describe all important harms or untended effects in a way that accounts for the design (for specific guidance, see CONSORT for harms)	Important harms or unintended effects in each treatment condition (for specific guidance see CONSORT for harms).
**Discussion**						
Limitations	20	Trial limitations, addressing sources of potential bias, imprecision, and if relevant, multiplicity of analyses. Consider potential carry-over effects.	Trial limitations, addressing sources of potential bias, imprecision, and, if relevant, multiplicity of analyses	Trial limitations, addressing sources of potential bias, imprecision, and, if relevant, multiplicity of analyses	Trial limitations, addressing sources of potential bias, imprecision, and if relevant, multiplicity of analyses. Consider potential carry over effects	Trial limitations, addressing sources of potential bias, imprecision, and, if relevant, multiplicity of analyses.
Generalisability	21	Generalisability (external validity, applicability) of the trial findings. Generalisability to clusters or individual participants, or both (as relevant).	Generalisability (external validity, applicability) of the trial findings	Generalisability (external validity, applicability) of the trial findings<br>*Extension:* Generalisability to clusters and/or individual participants (as relevant)	Generalisability (external validity, applicability) of the trial findings	Generalisability (external validity, applicability) of the trial findings. Generalisability to clusters or individual participants, or both (as relevant).
Interpretation	22	Interpretation consistent with results, balancing benefits and harms, and considering other relevant evidence.	Interpretation consistent with results, balancing benefits and harms, and considering other relevant evidence	Interpretation consistent with results, balancing benefits and harms, and considering other relevant evidence	Interpretation consistent with results, balancing benefits and harms, and considering other relevant evidence	Interpretation consistent with results, balancing benefits and harms, and considering other relevant evidence.
**Other information**						
Registration	23	Registration number and name of trial registry, or state the trial was not registered.	Registration number and name of trial registry	Registration number and name of trial registry	Registration number and name of trial registry	Registration number and name of trial registry.

Section / Topic	Item no.	CONSORT statement or extension<br>CRXO 2024	CONSORT 2010	Cluster 2012‡	Crossover 2019	Stepped-wedge 2018
Protocol	24	Where the full trial protocol and statistical analysis plan can be accessed, if available.	Where the full trial protocol can be accessed, if available	Where the full trial protocol can be accessed, if available	Where the full trial protocol can be accessed, if available	Where the full trial protocol can be accessed, if available.
Funding	25	Sources of funding and other support (such as supply of drugs), role of funders.	Sources of funding and other support (such as supply of drugs), role of funders	Sources of funding and other support (such as supply of drugs), role of funders	Sources of funding and other support (such as supply of drugs), role of funders	Sources of funding and other support (such as supply of drugs), and the role of funders.
Research ethics review	26	Whether the study was approved by a research ethics committee, with identification of the review committee(s).				Whether the study was approved by a research ethics committee, with identification of the review committee(s). Justification for any waiver or modification of informed consent requirements.
Data sharing	27	Where the individual de-identified participant data (including data dictionary), statistical code, and any other relevant documents/materials can be accessed.				
Patient and public involvement	28	Details of any patient and/or public involvement in the design, conduct and reporting of the trial; and, when applicable, other stakeholders' involvement.				

* The colour of the cell in the CRXO 2024 column indicates which statement or extension has most closely influenced the CRXO item (blue = standard CONSORT 2010; green = cluster 2012; grey = crossover 2019; yellow = stepped-wedge). Lighter shading indicates a minor change has been made to the wording of the influencing item; however, for inconsequential changes, the shading is unchanged (e.g. where reference in the item is made to a new CONSORT extension [item 6a]). For items where the wording has been slightly modified across statements, but the concept remains unchanged, the earliest statement or extension is highlighted. For example, while item 4a of the CRXO is the same as that of the stepped-wedge, the cell is highlighted green to indicate that the cluster extension is the influencing item, because the cluster extension was the first to introduce the requirement to report eligibility of clusters. If the CRXO item is sufficiently different from the other items, it is coloured orange.

† Note that items have been slightly edited by removing citations and footnotes within to simplify and avoid confusion.

‡ The CONSORT extension to cluster randomised trials provided *extensions* to the standard CONSORT 2010 items, rather than replacement items (as occurred in the crossover and stepped-wedge extensions).

¶ The standard CONSORT 2010 statement and design extensions for cluster, crossover and stepped-wedge all use '*sequence* generation'. In CONSORT CRXO we

use the term ‘*schedule*’ in place of ‘*sequence*’ to distinguish the randomly generated list of allocations (the schedule) from the sequence of treatments in the schedule. See Item 8a in CONSORT CRXO for further explanation.

Table S2. Comparison of items to report in journal or conference abstracts (item 1b) across the standard CONSORT 2010 statement and design extensions for CRXO, cluster, crossover and stepped-wedge*

	CONSORT statement or extension				
**Abstract item**	**CRXO 2024**	**CONSORT 2010†**	**Cluster 2012‡**	**Crossover 2019**	**Stepped-wedge 2018**
**Authors**		Contact details for the corresponding author*			
**Title**	Identification of study as a cluster randomised crossover trial	Identification of the study as randomised	Identification of study as randomised<br>*Extension:* Identification of study as cluster randomised	Identification of study as a randomised crossover trial	Identification of study as a stepped wedge cluster randomised trial
**Trial design**	Description of the trial design (including number of sequences, periods and clusters; and whether participants assessed in different periods are the same people, different people, or a mixture)	Description of the trial design (such as parallel, cluster, non-inferiority)	Description of the trial design (for example, parallel, cluster, non-inferiority)	Description of the trial design (crossover trial and number of periods)	Description of the trial design (including numbers of sequences and clusters, and whether participants assessed in different periods are the same people, different people, or a mixture)
**Methods:**					
Participants	Eligibility criteria for clusters and participants and the settings where the data were collected	Eligibility criteria for participants and the settings where the data were collected	Eligibility criteria for participants and the settings where the data were collected<br>*Extension:* Eligibility criteria for clusters	Eligibility criteria for participants and the settings where the data were collected	Eligibility criteria for clusters and participants
Intervention	The treatment conditions	Interventions intended for each group	Interventions intended for each group	Interventions intended for all participants	The intervention and control conditions
Objective	Specific objectives or hypotheses	Specific objective or hypothesis	Specific objective or hypothesis<br>*Extension:* Whether objective or hypothesis pertains to the cluster level,<br>the individual participant level, or both	Specific objective or hypothesis	Specific objective or hypothesis
Outcome	Clearly defined primary outcome	Clearly defined primary outcome for this report	Clearly defined primary outcome for this report<br>*Extension:* Whether the primary outcome pertains to the cluster level, the individual participant level or both	Clearly defined primary outcome for this report	Clearly defined primary outcome
Randomisation	How clusters were allocated to sequences of treatments	How participants were allocated to interventions	How participants were allocated to interventions	How participants were allocated to sequences	How clusters were allocated to sequence of treatments

Abstract item	CRXO 2024	CONSORT 2010†	Cluster 2012‡	Crossover 2019	Stepped-wedge 2018
			Extension: How clusters were allocated to interventions		
Blinding (masking)	Whether or not participants, healthcare professionals, those recruiting and those assessing outcomes were blinded	Whether participants, care givers, and those assessing the outcomes were blinded to group assignment	Whether or not participants, care givers, and those assessing the outcomes were blinded to group assignment	Whether or not participants, care givers, and those assessing the outcomes were blinded to intervention	Whether participants, healthcare professionals, those recruiting and those assessing outcomes were blinded
**Results:**					
Numbers randomised	Number of clusters randomised to each sequence of treatments	Number of participants randomised to each group	Number of participants randomised to each group *Extension:* Number of clusters randomised to each group	Number of participants randomised to each sequence	Number of clusters randomised to each sequence of treatments
Recruitment¶	Trial status	Trial status	Trial status	Trial status	Trial status
Numbers analysed	Number of clusters included in the analysis. Number of observations in the analysis for each treatment condition.	Number of participants analysed in each group	Number of participants analysed in each group *Extension:* Number of clusters analysed in each group	Number of participants analysed	Number of observations and clusters included in the analysis
Outcome	For the primary outcome, summary statistics by treatment condition and the estimated effect size (confidence interval)	For the primary outcome, a result for each group and the estimated effect size and its precision	For the primary outcome, a result for each group and the estimated effect size and its precision *Extension:* Results at the cluster or individual level as applicable for each primary outcome	For the primary outcome, the estimated effect size and its precision based on within participant comparisons	For the primary outcome, the estimated effect size (confidence interval) and reporting of any adjustment for secular trends
Harms	Important adverse events or side effects (even if not observed), or that harms were not assessed§	Important adverse events or side effects	Important adverse events or side effects	Important adverse events or side effects	Important adverse events or side effects
**Conclusions**	General interpretation of the results	General interpretation of the results	General interpretation of the results	General interpretation of the results	General interpretation of the results
**Trial registration**	Registration number and name of trial register	Registration number and name of trial register	Registration number and name of trial register	Registration number and name of trial register	Registration number and name of trial register
**Funding**	Source of funding	Source of funding	Source of funding	Source of funding	
**Research ethics review**	Ethical approvals				Ethical approvals

* The colour of the cell in the CRXO 2024 column indicates which statement or extension has most closely influenced the CRXO item (blue = standard CONSORT 2010; green = cluster 2012; grey = crossover 2019; yellow = stepped-wedge). Lighter shading indicates a minor change has been made to the wording of the influencing item; however, for inconsequential changes, the shading is unchanged. For items where the wording has been slightly modified across statements, but

the concept remains unchanged, the earliest statement or extension is highlighted. If the CRXO item is sufficiently different from the other items, it is coloured orange.

† CONSORT 2010 statement includes abstract items from the CONSORT extension for reporting randomised trials in journal and conference abstracts (1). Note that the wording in CONSORT for abstracts for blinding was “Whether **or not** participants, care givers, and those assessing the outcomes were blinded to group assignment”.

‡ The CONSORT extension to cluster randomised trials provided *extensions* to the standard CONSORT 2010 items, rather than replacement items (as occurred in the crossover and stepped-wedge extensions).

¶ Relevant to conference abstracts.

§ Informed by the explanation for item 1b from CONSORT Harms 2022 statement (2).

Table S3. Evidence of reporting quality*

**Section** / Topic	Item no.	Checklist item	Evidence
**Title and Abstract**			
	1a	Identification as a cluster randomised crossover trial in the title.	In a review of CRXO trials (3), 7/83 (8%) were identified as such in the title.
	1b	Structured summary of the trial design, methods, results, and conclusions (see separate CRXO checklist for abstracts)	In a review of CRXO trials (3), 21/83 (25%) were identified as such in the abstract.
**Introduction**			
Background and objectives	2a	Scientific background and explanation of rationale.	
	2b	Specific objectives or hypotheses.	
**Methods**			
Trial design	3a.1	Rationale for a cluster crossover design.	In a review of CRXO trials (3), 20/83 (24%) reported a rationale for using a cluster design *and* a crossover of interventions at the cluster level.
	3a.2	Description of the realised trial design including:<br>• number of treatment conditions;<br>• definition of cluster (i.e. the unit of randomisation);<br>• number and duration of periods;<br>• number and composition of sequences (e.g. ABAB, BABA);<br>• number of clusters randomised to each sequence;<br>• duration of any washout periods;<br>• whether the participants assessed in different periods are the same people, different people, or a mixture of the two; and<br>• consideration of potential for carryover effects.<br>A diagram of the trial is recommended when there are more than two periods and/or treatment conditions.	In a review of CRXO trials (3):<br>• 79/83 (95%) reported the number of clusters;<br>• 76/83 (92%) reported the number of periods;<br>• 77/83 (93%) reported a definition of the cluster;<br>• 83/83 (100%) reported whether the design was a cohort, repeated cross-sectional, or mixture of designs;<br>• 83/83 (100%) reported the use of a washout period;<br>• 17/83 (20%) discussed the potential for carryover to occur; and,<br>• 23/83 (28%) provided a schematic representation of the design.
	3b	Important changes to planned methods after trial commencement (such as eligibility criteria), with reasons.	

**Section** / Topic	Item no.	Checklist item	Evidence
Participants	4a	Eligibility criteria for clusters and participants.	In reviews examining cluster randomised trials, the reporting of eligibility criteria for clusters has been shown to vary (e.g. reported in 9/23 (39%) cluster trials in oral health (4), 96/106 (91%) in children (5), “most reports”/34 in primary care (6)).
	4b	Settings and locations where the data were collected.	
Intervention	5	The treatment conditions with sufficient details to allow replication, and whether they were delivered at the level of the cluster, the individual, or both.	
Outcomes	6a	Completely defined pre-specified primary and secondary outcome measures, including how and when they were assessed (for specific guidance see CONSORT for outcomes).	
	6b	Any changes to trial outcomes after the trial commenced, with reasons.	
Sample size	7a	How sample size was determined. Method of calculation and relevant parameters with sufficient detail so the calculation can be reproduced. Assumptions made about correlations between outcomes of participants from the same cluster (see separate CRXO checklist for sample size items).	In a review of CRXO protocols and trials (7), methodology and reporting of sample size calculations has been shown to be inadequate: only 53/91 (58%) provided a sample size calculation (with 39/53 (74%) of these providing sufficient detail to allow replication); and, only 9/91 (10%) used a sample size methodology that was appropriate for the CRXO design.
	7b	When applicable, explanation of any interim analyses and stopping guidelines.	
Randomisation:			
Schedule generation	8a	Method used to generate the random allocation schedule.	In a review of CRXO trials (3), 36/83 (43%) reported the method used to generate the random allocation schedule.
	8b	Type of randomisation; details of any restricted randomisation, if used.	In a review of CRXO trials (3), 30/83 (33%) reported using restricted randomisation (i.e. stratification, minimisation, matching).

**Section** / Topic	Item no.	Checklist item	Evidence
Allocation concealment mechanism	9	Specification that allocation was based on clusters; description of any methods used to conceal the allocation from the clusters until after their recruitment.	In a review of CRXO trials (3), 40/83 (48%) reported whether the people allocating the intervention sequence to the clusters knew the allocation sequence.
Implementation	10a	Who generated the random allocation schedule, who enrolled clusters, and who assigned clusters to sequences of treatments in the schedule.	
	10b	Mechanism by which individual participants were included in clusters for the purposes of the trial (such as complete enumeration or random sampling; continuous recruitment or ascertainment, or recruitment at a fixed point in time), including who recruited or identified participants.	In a review of CRXO trials (3), 44/57 (77%) reported whether the people recruiting / identifying participants knew which intervention sequence had been assigned to the cluster.
	10c	Whether consent was sought, from whom, when and for what; whether this differed between treatment conditions. Justification for any waiver or modification of informed consent requirements.	In a review of CRXO trials (3), 60/83 (72%) reported information about who provided consent to receive the intervention. In those trials where consent to receive the intervention was sought from individual participants (or another person on their behalf), 14/30 (47%) did not adequately report whether the participant had knowledge of the intervention they would receive prior to consenting, thus hindering judgements about the potential for bias arising from recruitment.
Blinding	11a	Who was blinded after assignment to sequences (e.g. cluster level participants, individual level participants, those assessing outcomes) and how.	In a review of CRXO trials (3), >80% reported whether individual-level and cluster-level participants were blinded to the treatment allocation. This was not the case for outcome assessment, where the blinding status of the outcome assessor (when not an individual-level participant) was clear in only 45/69 (65%) of trials.
	11b	If relevant, description of the similarity of interventions.	
Statistical methods	12a	Target estimand for each primary and secondary outcome including whether it pertains to the cluster-level or individual-level; statistical methods for their estimation including how period effects, clustering and repeated	In a review of CRXO trials (3):<br>• 78/83 (94%) reported whether the analysis was performed at the cluster or individual level;

**Section** / Topic	Item no.	Checklist item	Evidence
		measures were taken into account. Any assessment of carryover effects should be reported.	• 0/83 (0%) reported whether a single correlation was assumed for the within-cluster between-period correlation when there were more than two periods.
	12b	Methods for additional analyses, such as subgroup analyses, sensitivity analyses, and adjusted analyses.	
**Results**			
Participant flow (a diagram is strongly recommended)	13a	The numbers of clusters that were assessed for eligibility and were randomly assigned to each sequence. For each sequence-period (i.e. each cell) or treatment condition:<br>• the numbers of clusters that received intended treatments and were analysed for the primary outcome; and,<br>• the numbers of participants who were assessed for eligibility, received intended treatments and were analysed for the primary outcome.	Studies examining the use and completeness of information reported in CONSORT flow diagrams have found they are commonly missing or when used, have missing information (8-10).
	13b	For each sequence-period (i.e. each cell) or treatment condition, losses and exclusions for both clusters and participants with reasons.	
Recruitment	14a	Dates of treatment periods and washout periods.	In a review of CRXO trials (3), all reported use of a washout period (83/83; 100%).<br><br>In a review examining the reporting of cluster randomised trials in critical care medicine (11), 11/21 (52%) of the included CRXO trials provided details of a washout period.
	14b	Why the trial ended or was stopped.	
Baseline data	15	A table showing baseline cluster level characteristics by sequence, and individual level characteristics for each sequence-period (i.e. each cell) or treatment condition.	In a review of CRXO trials (3), 24/83 (29%) did not present a table of baseline characteristics, 37 (45%) reported baseline characteristics by treatment condition, seven (8%) by sequence, five (6%) separated in some way by period, two (2%)

**Section** / Topic	Item no.	Checklist item	Evidence
			separated by cluster only, but none separated by sequence and period.
Numbers analysed	16	The number of observations and clusters included in each analysis for each treatment condition and whether the analysis was according to the allocated schedule.	
Outcomes and estimation	17a	For each primary and secondary outcome, summary statistics by sequence-period (i.e. each cell) or treatment condition; the estimated effect size and its precision (e.g. 95% confidence interval); and any within-cluster correlations or variance components estimated in the analysis.	In a review of CRXO trials (3), a coefficient for the within-cluster within-period correlation and within-cluster between-period correlation, or other measure (such as variance components), for each primary outcome, was not reported in any of the included trials (0/83; 0%).<br><br>In a review examining the reporting of cluster randomised trials in critical care medicine (11), 2/21 (10%) provided estimates of correlations.
	17b	For binary outcomes, presentation of both absolute and relative effect sizes is recommended.	In a review of cluster randomised trial reports published in 2017 (12), 13/73 (18%) were found to report both a relative and absolute measure.<br><br>In a review of parallel group individually randomised trial reports published in January 2019 (13), 16/198 (8%) were found to report both a relative and an absolute measure.
Ancillary analyses	18	Results of any other analyses performed, including subgroup analyses, sensitivity analyses, and adjusted analyses, distinguishing pre-specified from exploratory.	
Harms	19	Important harms or unintended effects in each treatment condition (for specific guidance see CONSORT Harms 2022 statement).	
**Discussion**			
Limitations	20	Trial limitations, addressing sources of potential bias, imprecision, and if relevant, multiplicity of analyses. Consider potential carry-over effects.	In a review of CRXO trials (3), only 17/83 (20%) discussed the possibility of carryover.

**Section** / Topic	Item no.	Checklist item	Evidence
Generalisability	21	Generalisability (external validity, applicability) of the trial findings. Generalisability to clusters or individual participants, or both (as relevant).	
Interpretation	22	Interpretation consistent with results, balancing benefits and harms, and considering other relevant evidence.	
**Other information**			
Registration	23	Registration number and name of trial registry, or state the trial was not registered.	
Protocol	24	Where the full trial protocol and statistical analysis plan can be accessed, if available.	
Funding	25	Sources of funding and other support (such as supply of drugs), role of funders.	
Research ethics review	26	Whether the study was approved by a research ethics committee, with identification of the review committee(s).	In a review of cluster randomised designs (14), 77/300 (26%) failed to report ethics review.<br><br>In a review of stepped-wedge trials (15), 8/32 (25%) failed to report review by a research ethics committee.
Data sharing	27	Where the individual de-identified participant data (including data dictionary), statistical code, and any other relevant documents/materials can be accessed.	
Patient and public involvement	28	Details of any patient and/or public involvement in the design, conduct and reporting of the trial; and, when applicable, other stakeholders' involvement.	In a review examining the extent of patient engagement activities in published trials (16), only 23/371159 (0.01%) were estimated to report such activities.

* The evidence presented in the table primarily relates to the quality of reporting of CRXO trials; however, for some items, we draw upon broader evidence (e.g. other cluster designs).

References

1. Hopewell S, Clarke M, Moher D, et al. CONSORT for reporting randomised trials in journal and conference abstracts. Lancet. 2008;371:281-3.
2. Junqueira DR, Zorzela L, Golder S, et al. CONSORT Harms 2022 statement, explanation, and elaboration: updated guideline for the reporting of harms in randomised trials. BMJ. 2023;381:e073725.
3. Arnup SJ, Forbes AB, Kahan BC, Morgan KE, McKenzie JE. The quality of reporting in cluster randomised crossover trials: proposal for reporting items and an assessment of reporting quality. Trials. 2016;17:575.
4. Froud R, Eldridge S, Diaz Ordaz K, Marinho VCC, Donner A. Quality of cluster randomized controlled trials in oral health: a systematic review of reports published between 2005 and 2009: Quality of cluster randomized trials in oral health. Community dentistry and oral epidemiology. 2012;40:3-14.
5. Walleser S, Hill SR, Bero LA. Characteristics and quality of reporting of cluster randomized trials in children: reporting needs improvement. Journal of Clinical Epidemiology. 2011;64:1331-40.
6. Eldridge S, Ashby D, Bennett C, Wakelin M, Feder G. Internal and external validity of cluster randomised trials: systematic review of recent trials. BMJ. 2008;336:876-80.
7. Arnup SJ, Forbes AB, Kahan BC, Morgan KE, McKenzie JE. Appropriate statistical methods were infrequently used in cluster-randomized crossover trials. J Clin Epidemiol. 2016;74:40-50.
8. Hopewell S, Hirst A, Collins GS, Mallett S, Yu LM, Altman DG. Reporting of participant flow diagrams in published reports of randomized trials. Trials. 2011;12:253.
9. Meyer H-GJ, Pandis N, Seehra J, Faggion CM. Reporting of flow diagrams in randomised controlled trials published in periodontology and implantology: a survey. BMC Med Res Methodol. 2023;23:105.
10. Rønsbo TN, Laigaard J, Pedersen C, Mathiesen O, Karlsen APH. Adherence to participant flow diagrams in trials on postoperative pain management after total hip and knee arthroplasty: a methodological review. Trials. 2021;22:280.
11. Cook DJ, Rutherford WB, Scales DC, Adhikari NKJ, Cuthbertson BH. Rationale, Methodological Quality, and Reporting of Cluster-Randomized Controlled Trials in Critical Care Medicine: A Systematic Review. Crit Care Med. 2021;49:977-87.
12. Turner EL, Platt AC, Gallis JA, et al. Completeness of reporting and risks of overstating impact in cluster randomised trials: a systematic review. Lancet Glob Health. 2021;9:e1163-e8.
13. Rombach I, Knight R, Peckham N, Stokes JR, Cook JA. Current practice in analysing and reporting binary outcome data-a review of randomised controlled trial reports. BMC Med. 2020;18:147.
14. Taljaard M, McRae AD, Weijer C, et al. Inadequate reporting of research ethics review and informed consent in cluster randomised trials: review of random sample of published trials. BMJ. 2011;342:d2496.
15. Taljaard M, Hemming K, Shah L, Giraudeau B, Grimshaw JM, Weijer C. Inadequacy of ethical conduct and reporting of stepped wedge cluster randomized trials: Results from a systematic review. Clin Trials. 2017:1740774517703057.
16. Fergusson D, Monfaredi Z, Pussegoda K, et al. The prevalence of patient engagement in published trials: a systematic review. Res Involv Engagem. 2018;4:17.
