# Supplementary material for: Reporting of cluster randomised crossover trials: extension of the CONSORT 2010 statement with explanation and elaboration
Source: BMJ. 2025 Jan 6;388:e080472. doi: 10.1136/bmj-2024-080472 (PMC11701780; doi:10.1136/bmj-2024-080472)
Supplement: Supplementary file 3 — Web appendix 3: Supplementary material 3 [file mckj080472.ww3.pdf]

Supplementary material 3 for ‘Reporting of cluster randomised crossover trials: extension of the CONSORT 2010 statement with explanation and elaboration’

McKenzie JE*, Taljaard M, Hemming K, Arnup SJ, Giraudeau B, Eldridge S, Hooper R, Kahan BC, Li T, Moher D, Grimshaw JM, Forbes AB. BMJ 2025;388:e080472. doi:10.1136/bmj-2024-080472

* Correspondence to: Professor Joanne McKenzie, School of Public Health and Preventive Medicine, Monash University, 553 St Kilda Road, Melbourne, Victoria 3004, Australia
Email: joanne.mckenzie@monash.edu

Table of contents:

Table S1. Adapted version of the abstract from the Pourrat *et al* CRXO trial (1) demonstrating reporting against the abstract checklist items. ........ 2

Table S2. Example of the baseline table structured by sequence-period from the Bridge-It CRXO trial (two-treatment, two-period, two-sequence cross-sectional design) (2) ........ 3

Table S3. Example of the baseline table structured by treatment condition from the BETR Disinfection Study (four-treatment, four-period, nine-sequence cross-sectional design) (3) ........ 4

Table S4. Example of a table with the numbers of clusters and participants reported for each period within each sequence from the Pourrat *et al* CRXO trial (two-treatment, two-period, two-sequence cross-sectional design) (1) ........ 5

Table S5. Example of a table with the within-period and between-period intra-cluster correlation coefficients from the Pourrat et al CRXO trial (two-treatment, two-period, two-sequence cross-sectional design) (1) ........ 6

Table S6. Example of a table presenting the results of post-hoc sensitivity analyses from the PEPTIC CRXO trial (two-treatment, two-period, two-sequence cross-sectional design) (4) ........ 7

Figure S1. Example of a timeline cluster diagram from the Pourrat *et al* CRXO trial (two-treatment, two-period, two-sequence cross-sectional design) (1) ........ 8

Figure S2. Example of a flow diagram structured by sequence-period from the Pourrat *et al* CRXO trial (two-treatment, two-period, two-sequence cross-sectional design) (1) ........ 10

Figure S3. Example of a flow diagram structured by treatment condition from the MedBridge CRXO trial (three-treatment, six-period, five-sequence cross-sectional design) (5). [Note that the diagram does not include information on the number of clusters that received the treatments or the number that were analysed for the primary outcome.] ........ 11

Acknowledgements ........ 12

References ........ 13

Table S1. Adapted version of the abstract from the Pourrat *et al* CRXO trial (1) demonstrating reporting against the abstract checklist items.

*Title:* Effectiveness of a multicomponent pharmacist intervention at hospital discharge for drug-related problems: A cluster randomised cross-over trial

*Aims:* The aim of this study was to assess whether a pharmacist intervention associating medication reconciliation at discharge with a link to the community pharmacist *compared with usual pharmaceutical care (i.e. no systematic reconciliation done by a pharmacist)* reduces drug-related problems (DRP) in adult patients during the 7 days after hospital discharge in 22 university or general hospitals in France.

*Methods:* We conducted a *cross-sectional two-period (each 14-days) two-sequence* cluster randomised cross-over superiority trial with hospital units as the cluster unit. *Randomisation was stratified by hospital, with balanced allocation of units to the two sequences within.* The primary outcome was a composite of any kind of DRP (prescription/dispensation, patient error or gap due to no medication available) during the 7 days after discharge, assessed by phone with the patient and community pharmacist. Among secondary outcomes, we studied self-reported unplanned hospitalisations at day 35 after discharge and severe iatrogenic problems. *Hospital and community pharmacists, and outcome assessors were not blinded to intervention. An exception was outcome assessors judging the potential medical impact of the DRP, who were blinded. Patients recruited in the intervention period were not blinded, while those in the control periods were blinded to the study hypothesis.*

*Results:* A total of 1092 patients (538 in the experimental periods and 554 in the control periods) were enrolled in 48 units *(24 receiving intervention followed by control, 24 vice-versa)*. Three patients refused to have their data analysed and were excluded from the analyses. As compared with usual care, the pharmacist intervention led to a lower proportion of patients with at least one DRP (44.0% vs 50.6%; odds ratio [OR] 0.77, 95% confidence interval [CI] 0.61–0.98) and severe iatrogenic problems (5.2% vs 8.7%; OR 0.57, 95% CI 0.35–0.93) but no significant difference in unplanned hospitalisations at day 35 (5.8% vs 4.5%; OR 1.46, 95% CI 0.91–2.35).

*Conclusion:* Medication reconciliation associated with communication between the hospital and community pharmacist may decrease patient exposure to DRP and severe iatrogenic problems but not unplanned hospitalisation. However, this intervention could be recommended in health policies to improve drug management.

*Trial registration: ClinicalTrials.gov (NCT02006797)*
*Research ethics review: Local ethics committee for all centres (CPP TOURS - Region Centre - Ouest 1)*
*Source of funding: French Ministry of Health, Grant/Award Number: PREPS 12-010-0054*

Red italicised text indicates additional text to that in the published abstract.

Table S2. Example of the baseline table structured by sequence-period from the Bridge-It CRXO trial (two-treatment, two-period, two-sequence cross-sectional design) (2)

| | Intervention group, period 1 (N=229) | Intervention group, period 2 (N=86) | Control group, period 1 (N=161) | Control group, period 2 (N=157) |
|---|---|---|---|---|
| **Age, years** | | | | |
| Mean | 23·2 (6·0) | 21·4 (4·8) | 22·2 (4·4) | 22·9 (5·8) |
| **Methods of contraception used** | | | | |
| Combined hormonal contraceptive (pill, patch, or ring) | 114 (49·8%) | 41 (47·7%) | 99 (61·5%) | 94 (59·9%) |
| Progestogen-only pill | 39 (17·0%) | 19 (22·1%) | 35 (21·7%) | 32 (20·4%) |
| Male condom | 189 (82·5%) | 66 (76·7%) | 117 (72·7%) | 135 (86·0%) |
| Progestogen-only injectable | 14 (6·1%) | 6 (7·0%) | 10 (6·2%) | 18 (11·5%) |
| Progestogen-only implant | 29 (12·7%) | 10 (11·6%) | 23 (14·3%) | 19 (12·1%) |
| Copper-bearing intrauterine device | 6 (2·6%) | 0 | 4 (2·5%) | 4 (2·5%) |
| Levonorgestrel-releasing intrauterine system | 0 | 1 (1·2%) | 4 (2·5%) | 2 (1·3%) |
| Withdrawal method | 65 (28·4%) | 28 (32·6%) | 52 (32·3%) | 67 (42·7%) |
| Other methods* | 10 (4·4%) | 6 (7·0%) | 7 (4·3%) | 9 (5·7%) |
| Never used any method | 8 (3·5%) | 4 (4·7%) | 10 (6·2%) | 2 (1·3%) |
| **Sexual and reproductive history** | | | | |
| Previous birth | 30 (13·1%) | 5 (5·8%) | 7 (4·3%) | 13 (8·3%) |
| Previous termination | 38 (16·6%) | 6 (7·0%) | 22 (13·7%) | 27 (17·2%) |
| Previous miscarriage | 17 (7·4%) | 5 (5·8%) | 10 (6·2%) | 6 (3·8%) |
| Current sexual relationship | 176 (76·9%) | 55 (64·0%) | 104 (64·6%) | 111 (70·7%) |
| First time use of emergency contraception | 52 (22·7%) | 22 (25·6%) | 28 (17·4%) | 32 (20·4%) |
| **Number of times emergency contraception used in past 12 months** | | | | |
| Mean | 1·4 (1·4) | 1·5 (1·5) | 1·5 (1·2) | 1·7 (2·0) |
| Median | 1·0 (0·0–2·0) | 1·0 (1·0–2·0) | 1·0 (1·0–2·0) | 1·0 (1·0–2·0) |
| Minimum, maximum | 0·0–8·0 | 0·0–9·0 | 0·0–6·0 | 0·0–20·0 |
| **Ethnic background** | | | | |
| White | 157 (68·6%) | 60 (69·8%) | 98 (60·9%) | 114 (72·6%) |
| Asian or Asian British | 21 (9·2%) | 6 (7·0%) | 8 (5·0%) | 21 (13·4%) |
| Black or Black British | 29 (12·7%) | 12 (14·0%) | 36 (22·4%) | 15 (9·6%) |
| Mixed or other | 19 (8·3%) | 6 (7·0%) | 17 (10·6%) | 6 (3·8%) |
| Not specified | 3 (1·3%) | 2 (2·3%) | 2 (1·2%) | 1 (0·6%) |

Data are mean (SD), N (%), or median (25th, 75th percentile). N is the number of women recruited. The proportion of women with previous history of ectopic pregnancy was less than 1% in all groups. *Other methods of protection were female condom, cap or diaphragm, vasectomy, fertility awareness, and emergency contraception.

*Table 1:* **Baseline characteristics**

Table S3. Example of the baseline table structured by treatment condition from the BETR Disinfection Study (four-treatment, four-period, nine-sequence cross-sectional design) (3)

| | Reference (n=4916) | UV group (n=5178) | Bleach group (n=5438) | Bleach and UV group (n=5863) |
|---|---|---|---|---|
| **Demographics*** | | | | |
| Mean age (SD) | 57·9 (20·9) | 58·5 (21·3) | 58·6 (20·7) | 57·7 (21·8) |
| Race | | | | |
| White | 3042 (63%) | 3228 (65%) | 3416 (64%) | 3747 (64%) |
| African American | 1418 (30%) | 1411 (28%) | 1591 (30%) | 1655 (28%) |
| Other | 243 (5%) | 233 (5%) | 249 (5%) | 329 (6%) |
| Unknown | 102 (2%) | 97 (2%) | 95 (2%) | 111 (2%) |
| Male sex | 2475 (51%) | 2518 (51%) | 2768 (52%) | 3017 (52%) |
| **Comorbidities*** | | | | |
| Median Charlson index (IQR) | 2 (1–4) | 2 (0–4) | 2 (1–4) | 2 (0–4) |
| Myocardial infarction | 499 (11%) | 457 (10%) | 475 (9%) | 583 (10%) |
| Congestive heart failure | 937 (20%) | 950 (20%) | 1014 (20%) | 1151 (21%) |
| Cerebrovascular disease | 571 (12%) | 540 (11%) | 582 (11%) | 610 (11%) |
| Hemiplegia or paraplegia | 97 (2%) | 118 (2%) | 139 (3%) | 166 (3%) |
| Peripheral vascular disease | 450 (10%) | 498 (10%) | 524 (10%) | 543 (10%) |
| Dementia | 75 (2%) | 101 (2%) | 138 (3%) | 111 (2%) |
| COPD | 1248 (27%) | 1325 (28%) | 1339 (26%) | 1516 (27%) |
| Rheumatic disease | 161 (3%) | 181 (4%) | 183 (4%) | 224 (4%) |
| Peptic ulcer disease | 143 (3%) | 97 (2%) | 126 (2%) | 178 (3%) |
| Liver disease | | | | |
| Mild | 475 (10%) | 452 (9%) | 484 (9%) | 557 (10%) |
| Moderate or severe | 120 (3%) | 142 (3%) | 135 (3%) | 177 (3%) |
| Diabetes mellitus | 1302 (28%) | 1248 (26%) | 1371 (27%) | 1505 (27%) |
| Complicated | 303 (7%) | 273 (6%) | 350 (7%) | 350 (6%) |
| Renal disease | 980 (21%) | 986 (21%) | 1083 (21%) | 1171 (21%) |
| Malignancy | 842 (18%) | 807 (17%) | 864 (17%) | 961 (17%) |
| Metastatic solid tumour | 305 (7%) | 340 (7%) | 330 (6%) | 367 (7%) |
| AIDS/HIV | 49 (1%) | 48 (1%) | 52 (1%) | 55 (1%) |
| Arrhythmia | 1542 (33%) | 1425 (30%) | 1513 (30%) | 1824 (33%) |
| Valvular heart disease | 420 (9%) | 415 (9%) | 412 (8%) | 566 (10%) |
| Pulmonary circulation disease | 386 (8%) | 452 (9%) | 439 (9%) | 495 (9%) |
| Hypertension | 2264 (49%) | 2237 (47%) | 2336 (46%) | 2709 (48%) |
| Complicated | 826 (18%) | 839 (18%) | 959 (19%) | 1007 (18%) |
| Other neurological disease | 682 (15%) | 665 (14%) | 671 (13%) | 792 (14%) |
| Hypothyroid disease | 523 (11%) | 545 (11%) | 599 (12%) | 637 (11%) |

Data are n (%) unless stated otherwise. *The majority of patient-specific data were not available from one study hospital because of changes in electronic health record systems as follows: age (406 had data missing), race (428 missing), sex (407 missing), comorbidity data (1267 missing). COPD=chronic obstructive pulmonary disease.

***Table 1:* Baseline characteristics**

Table S4. Example of a table with the numbers of clusters and participants reported for each period within each sequence from the Pourrat *et al* CRXO trial (two-treatment, two-period, two-sequence cross-sectional design) (1)

**TABLE 2** Drug-related problems observed during the 7 days after hospital discharge

| Outcome | Control/intervention sequence | | Intervention/control sequence | | Risk difference (%) (95% CI) | Odds ratio (95% CI) |
|---|---|---|---|---|---|---|
| | Period 1: Control 24 units, *n* = 307 | Period 2: Intervention 24 units, *n* = 258 | Period 1: Intervention 24 units, *n* = 278 | Period 2: Control 24 units, *n* = 246 | | |
| At least one drug-related problem (ITT) | 160 (52.1) | 115 (44.6) | 121 (43.5) | 120 (48.8) | −6.55 (−12.49; −0.60) | 0.77 (0.61;0.98) |
| *At least one prescription/dispensation problem* | 18 (5.9) | 5 (1.9) | 13 (4.7) | 17 (6.9) | −3.19 (−5.71; −0.67) | 0.52 (0.29;0.93) |
| *At least one patient error* | 142 (46.3) | 104 (40.3) | 107 (38.5) | 100 (40.7) | −4.27 (−10.1;1.59) | 0.84 (0.66;1.07) |
| *At least one treatment missing* | 36 (11.7) | 25 (9.7) | 16 (5.8) | 27 (11.0) | −3.48 (−6.95; −0.01) | 0.65 (0.43;0.99) |
| | 24 units, *n* = 263 | 24 units, *n* = 233 | 24 units, *n* = 242 | 24 units, *n* = 233 | | |
| At least one drug-related problem (completers) | 160 (60.8) | 115 (49.4) | 121 (50.0) | 120 (51.5) | −6.64 (−12.9; −0.37) | 0.77 (0.60;0.99) |

Table S5. Example of a table with the within-period and between-period intra-cluster correlation coefficients from the Pourrat et al CRXO trial (two-treatment, two-period, two-sequence cross-sectional design) (1)

**TABLE 3** Within-period and between-period intra-cluster correlation coefficients

| Outcome | Within-period correlation | Between-period correlation |
|---|---|---|
| At least one drug-related problem (ITT) *n* = 1089 | 0.022 [0.000;0.051] | 0.003 [0.000;0.012] |
| *At least one prescription/dispensation problem* | 0.000 [0.000;0.019] | 0.000 [0.000;0.014] |
| *At least one patient error* | 0.019 [0.000;0.053] | 0.002 [0.000;0.013] |
| *At least one treatment missing* | 0.029 [0.000;0.070] | 0.015 [0.000;0.037] |
| At least one drug-related problem (completers) *n* = 971 | 0.030 [0.000;0.065] | 0.004 [0.000;0.015] |

ITT, intention to treat
Confidence intervals are obtained by a normal-based bootstrap approach with 10 000 replications

Table S6. Example of a table presenting the results of post-hoc sensitivity analyses from the PEPTIC CRXO trial (two-treatment, two-period, two-sequence cross-sectional design) (4)

**eTable 61.** Results of sensitivity analyses for the primary outcome (in-hospital mortality during index hospitalization within 90 days) using alternative analysis methods.

| Method and model description | Risk ratio (95% CI) |
|---|---|
| Main analysis: Generalised estimating equations (GEE) with full covariates[a], exchangeable working correlation, robust standard errors clustered by ICU | 1.05 (1.00 to 1.10) |
| GEE with full covariates, independence working correlation, robust standard errors clustered by ICU | 1.05 (1.00 to 1.11) |
| Generalised linear mixed model with full covariates, binary outcome, logarithmic link, random effects for site and site-period | 1.05 (1.00 to 1.11) |
| Generalised linear mixed model with full model, binary outcome, logarithmic link, random effects for site only | 1.05 (1.00 to 1.10) |
| Generalised linear model with full covariates, binary outcome, logarithmic link and fixed effects for site | 1.05 (1.00 to 1.11) |
| GEE with full covariates apart from proportion of patients in each site in PPI group, exchangeable working correlation, robust standard errors clustered by ICU | 1.05 (1.00 to 1.10) |
| GEE with full covariates and bootstrapped standard error with clustering by ICU (1000 replications) | 1.05 (1.00 to 1.10) |
| GEE with full covariates and bootstrap percentile 95% CI | 1.05 (1.00 to 1.10) |
| GEE with full covariates and bootstrap bias-corrected 95% CI | 1.05 (1.00 to 1.10) |
| GEE with full covariates and various small sample corrections to the robust/sandwich standard error, and use of standard normal (Z) or t-distribution with 32 degrees of freedom† | |
| Morel, Bokossa, Neerchal (MBN) Z | 1.05 (0.99 to 1.11) |
| MBN t-32 | 1.05 (0.99 to 1.11) |
| Fay & Graubard (FG) Z | 1.05 (1.00 to 1.10) |
| FG t-32 | 1.05 (1.00 to 1.10) |
| Mancl & DeRouen (MD) Z | 1.05 (0.99 to 1.11) |
| MD t-32 | 1.05 (0.99 to 1.11) |
| Kauermann & Carroll (KC) Z | 1.05 (0.99 to 1.10) |
| KC t-32 | 1.05 (0.99 to 1.11) |

a. The composition of "Full covariates" is as described in the Methods section: fixed effect terms for treatment group, proportion of patients in each ICU in the PPI group, order of administration, batch of randomisation, and batch-by-order interaction.
b. GEE small sample corrections used the xtgeebcv module in Stata[9]

Figure S1. Example of a timeline cluster diagram from the Pourrat *et al* CRXO trial (two-treatment, two-period, two-sequence cross-sectional design) (1)

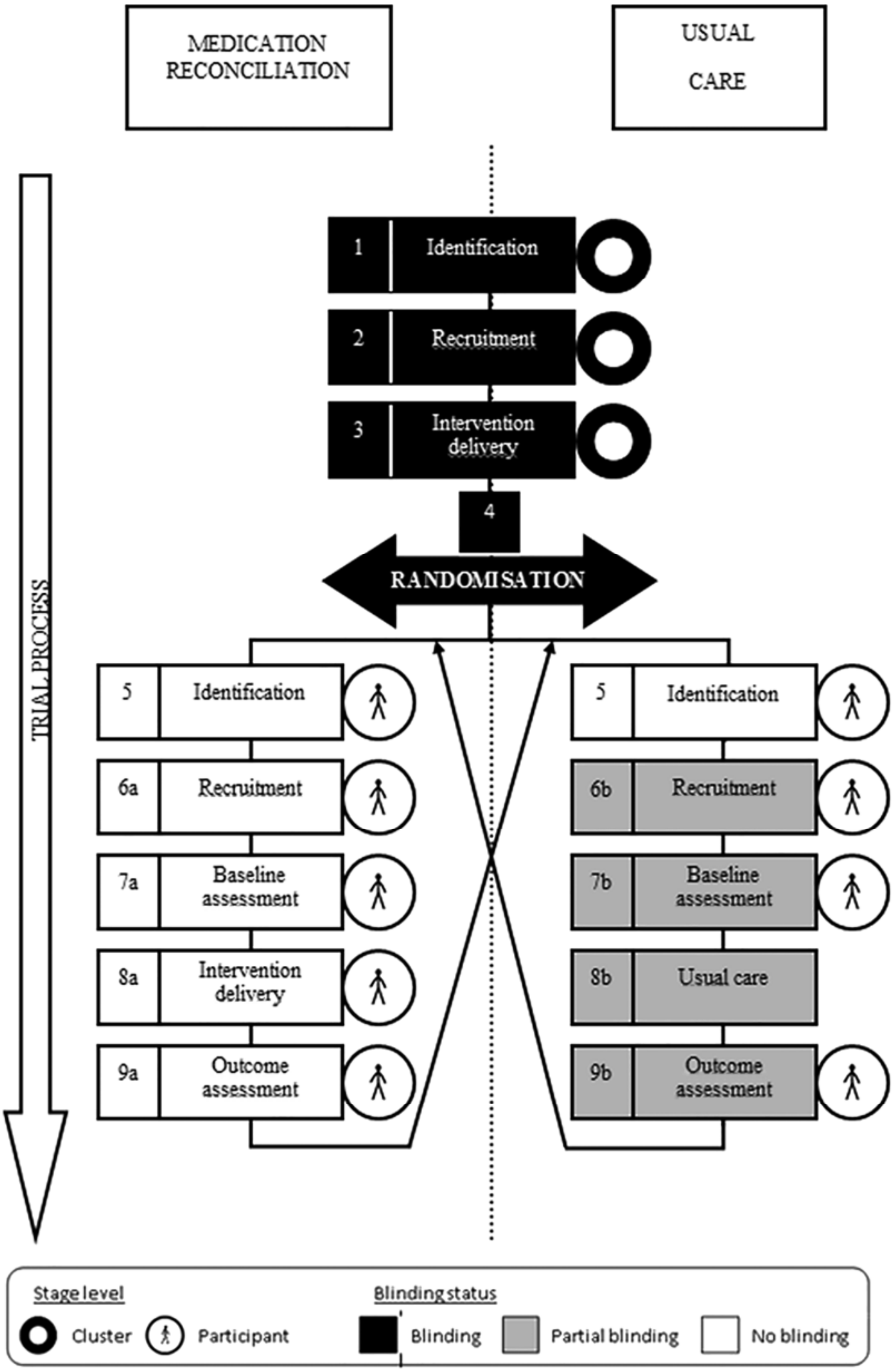

| | |
|---|---|
| 1 | Cluster identification<br>French hospital pharmacists are approached by the study team. Each hospital pharmacist who agrees to participate in the trial identifies 2 units from their hospital, one surgical and one medical unit. |
| 2 | Cluster recruitment<br>Medical heads from hospital units receive information and provide written consent to take part in the study. |
| 3 | Intervention delivery at cluster level<br>Hospital pharmacists are trained in medication reconciliation.<br>Community pharmacists working in nearby participating hospital units are informed of the study in 3 ways: an article in a professional journal supported by the pharmacist unions, in a professional journal supported by the national council of the order of pharmacists, and a letter from the study scientific committee distributed by wholesale drug distributors. |
| 4 | Randomisation: cross-over design<br>Randomisation is performed in a 1:1 ratio by an independent statistician with stratification on the hospital.<br>Each hospital unit is randomised to perform medication reconciliation or usual care for a first 14-day period and is crossed over to the other group for a second 14-day period. |
| 5 | Participant identification<br>In each hospital unit, unblinded hospital pharmacists identify eligible patients. |
| 6a | Participant recruitment in the medication reconciliation group<br>Participants are recruited by unblinded hospital pharmacists. They receive complete information and provide oral consent for intervention and for data collection. |
| 6b | Participant recruitment in the usual care group<br>Participants are recruited by unblinded hospital pharmacists. They receive partial information because they are not aware of the existence of the medication reconciliation group and provide oral consent for data collection. |
| 7a | Participant baseline data collection in the medication reconciliation group<br>Baseline data are collected by the unblinded hospital pharmacists. There is no blinding for patients.<br>Contact details for the patient's community pharmacist are collected. |
| 7b | Participant baseline data collection in the usual care group<br>Baseline data are collected by the unblinded hospital pharmacists. Patients are not aware of the existence of the medication reconciliation group.<br>Contact details for the patient's community pharmacist are collected. |
| 8a | Intervention delivery<br>Medication reconciliation at patient discharge is performed by a hospital pharmacist, followed by phone transmission of treatment modification to the patient's community pharmacist.<br>No blinding for hospital pharmacists, community pharmacists and patients. |
| 8b | Usual care<br>No blinding for community pharmacists, but they are not aware that the patient is involved in a trial.<br>No blinding for patients, but they are not aware of the existence of the medication reconciliation group. |
| 9a | Participant outcome assessment in the medication reconciliation group<br>Drug-related problem within 7 days after discharge assessed by a research pharmacist recruited for the study, using a standardised evaluation form. Assessment is centralised and performed by a phone call to both the participant and community pharmacist.<br>No blinding for the research pharmacist, community pharmacists and patients. |
| 9b | Participant outcome assessment in the usual care group<br>Drug-related problem within 7 days after discharge assessed by a research pharmacist recruited for the study, using a standardised evaluation form. Assessment is centralised and performed by a phone call to both the participant and the community pharmacist.<br>No blinding for the research pharmacist, community pharmacists are not aware that the patient is involved in a trial and patients are not aware of the existence of the medication reconciliation group. |

Figure S2. Example of a flow diagram structured by sequence-period from the Pourrat *et al* CRXO trial (two-treatment, two-period, two-sequence cross-sectional design) (1)

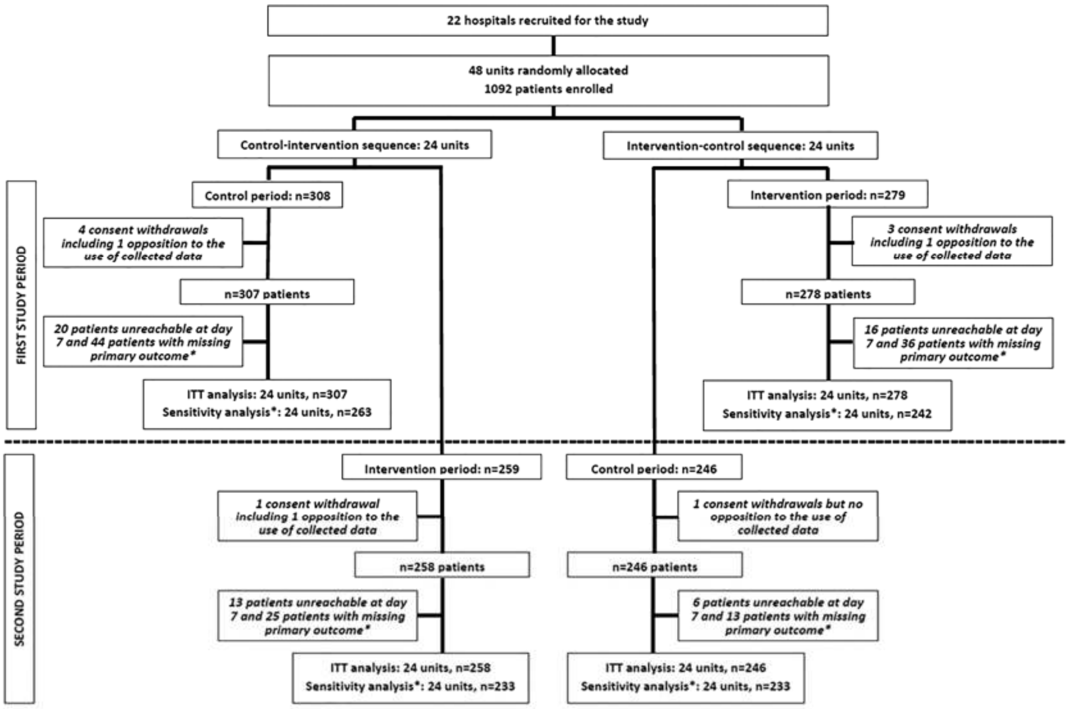


**FIGURE 4** Flowchart of the study

Figure S3. Example of a flow diagram structured by treatment condition from the MedBridge CRXO trial (three-treatment, six-period, five-sequence cross-sectional design) (5). [Note that the diagram does not include information on the number of clusters that received the treatments or the number that were analysed for the primary outcome.]

Figure 2. CONSORT Diagram of the Medication Reviews Bridging Healthcare Trial

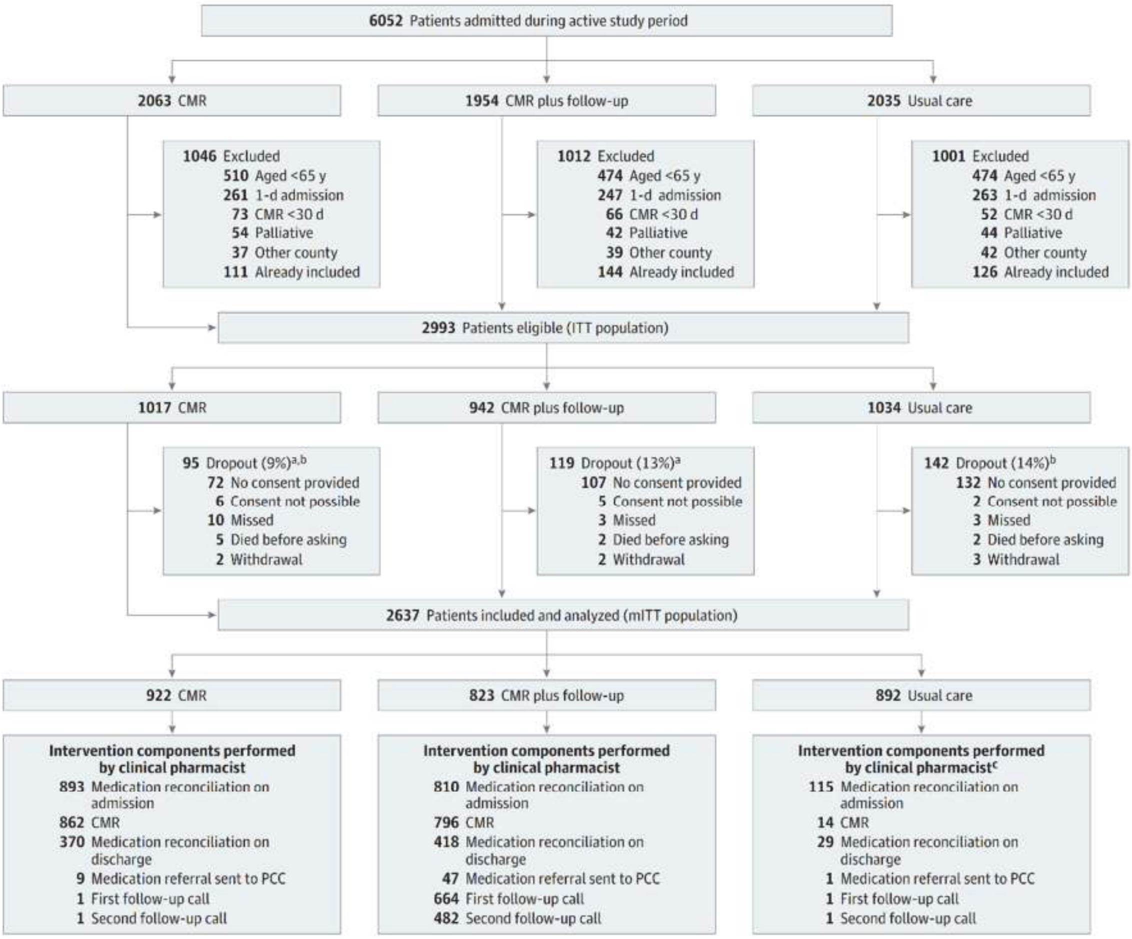


CMR indicates comprehensive medication review; CONSORT, Consolidated Standards of Reporting Trials; ITT, intention-to-treat; mITT, modified ITT; and PCC, primary care clinician.

[a] $P = .02$, $\chi^2$ test for differences in dropouts between CMR and usual care groups.

[b] $P < .01$, $\chi^2$ test for differences in dropouts between CMR plus follow-up and usual care groups.

[c] Indicates a protocol violation.

Acknowledgements

Table S3 was published in The Lancet, Vol. 389, Anderson DJ, Chen LF, Weber DJ, Moehring RW, Lewis SS, Triplett PF, Blocker M, Becherer P, Schwab JC, Knelson LP, Lokhnygina Y, Rutala WA, Kanamori H, Gergen MF, Sexton DJ; for the CDC Prevention Epicenters Program, Enhanced terminal room disinfection and acquisition and infection caused by multidrug-resistant organisms and Clostridium difficile (the Benefits of Enhanced Terminal Room Disinfection study): a cluster-randomised, multicentre, crossover study, Pages 805-814, Copyright Elsevier (2017).

Tables S4, S5, Figures S1, S2 are reprinted from British Journal Clinical Pharmacology, Vol. 86, Xavier Pourrat, Clémence Leyrat, Benoît Allenet, Brigitte Bouzige, Armelle Develay, Martial Fraysse, Valérie Garnier, Jean-Michel Halimi, Clarisse Roux-Marson, Bruno Giraudeau. Effectiveness of a multicomponent pharmacist intervention at hospital discharge for drug-related problems: A cluster randomised cross-over trial, Pages 2441-2454, Copyright (2020) The British Pharmacological Society, with permission from Wiley.

Table S6 reproduced with permission from JAMA. 2020. 323(7):616-626]. Copyright©(2020) American Medical Association. All rights reserved.

References

1. Pourrat X, Leyrat C, Allenet B, et al. Effectiveness of a multicomponent pharmacist intervention at hospital discharge for drug-related problems: A cluster randomised cross-over trial. Br J Clin Pharmacol. 2020;86:2441-54.
2. Cameron ST, Glasier A, McDaid L, et al. Use of effective contraception following provision of the progestogen-only pill for women presenting to community pharmacies for emergency contraception (Bridge-It): a pragmatic cluster-randomised crossover trial. Lancet. 2020;396:1585-94.
3. Anderson DJ, Chen LF, Weber DJ, et al. Enhanced terminal room disinfection and acquisition and infection caused by multidrug-resistant organisms and Clostridium difficile (the Benefits of Enhanced Terminal Room Disinfection study): a cluster-randomised, multicentre, crossover study. Lancet. 2017;389:805-14.
4. Peptic Investigators for the Australian New Zealand Intensive Care Society Clinical Trials Group, Alberta Health Services Critical Care Strategic Clinical Network,, the Irish Critical Care Trials Group,, Young PJ, Bagshaw SM, et al. Effect of Stress Ulcer Prophylaxis With Proton Pump Inhibitors vs Histamine-2 Receptor Blockers on In-Hospital Mortality Among ICU Patients Receiving Invasive Mechanical Ventilation: The PEPTIC Randomized Clinical Trial. JAMA. 2020;323:616-26.
5. Kempen TGH, Bertilsson M, Hadziosmanovic N, et al. Effects of Hospital-Based Comprehensive Medication Reviews Including Postdischarge Follow-up on Older Patients' Use of Health Care: A Cluster Randomized Clinical Trial. JAMA Netw Open. 2021;4:e216303.
