# Supplementary material for: Reporting of cluster randomised crossover trials: extension of the CONSORT 2010 statement with explanation and elaboration
Source: BMJ. 2025 Jan 6;388:e080472. doi: 10.1136/bmj-2024-080472 (PMC11701780; doi:10.1136/bmj-2024-080472)
Supplement: Supplementary file 4 — Web appendix 4: Checklist of information to include when reporting a cluster randomised crossover trial [file mckj080472.ww4.docx]

Supplementary materials 4: Checklist of information to include when reporting a cluster randomised crossover trial

| **Section** / Topic | Item no. | Checklist item | Page no. | Section heading* |
| --- | --- | --- | --- | --- |
| **Title and Abstract** |  |  |  |  |
|  | 1a | Identification as a cluster randomised crossover trial in the title. |  |  |
|  | 1b | Structured summary of the trial design, methods, results, and conclusions (see separate CRXO checklist for abstracts) |  |  |
| **Introduction** |  |  |  |  |
| Background and objectives | 2a | Scientific background and explanation of rationale. |  |  |
|  | 2b | Specific objectives or hypotheses. |  |  |
| **Methods** |  |  |  |  |
| Trial design | 3a.1 | Rationale for a cluster crossover design. |  |  |
|  | 3a.2 | Description of the realised trial design including:   - number of treatment conditions; - definition of cluster (i.e. the unit of randomisation); - number and duration of periods; - number and composition of sequences (e.g. ABAB, BABA); - number of clusters randomised to each sequence; - duration of any washout periods; - whether the participants assessed in different periods are the same people, different people, or a mixture of the two; and - consideration of potential for carryover effects.   A diagram of the trial is recommended when there are more than two periods and/or treatment conditions. |  |  |
|  | 3b | Important changes to planned methods after trial commencement (such as eligibility criteria), with reasons. |  |  |
| Participants | 4a | Eligibility criteria for clusters and participants. |  |  |
|  | 4b | Settings and locations where the data were collected. |  |  |
| Intervention | 5 | The treatment conditions with sufficient details to allow replication, and whether they were delivered at the level of the cluster, the individual, or both. |  |  |
| Outcomes | 6a | Completely defined pre-specified primary and secondary outcome measures, including how and when they were assessed (for specific guidance see CONSORT for outcomes). |  |  |
|  | 6b | Any changes to trial outcomes after the trial commenced, with reasons. |  |  |
| Sample size | 7a | How sample size was determined. Method of calculation and relevant parameters with sufficient detail so the calculation can be reproduced. Assumptions made about correlations between outcomes of participants from the same cluster (see separate CRXO checklist for sample size items). |  |  |
|  | 7b | When applicable, explanation of any interim analyses and stopping guidelines. |  |  |
| Randomisation: |  |  |  |  |
| Schedule generation | 8a | Method used to generate the random allocation schedule. |  |  |
|  | 8b | Type of randomisation; details of any restricted randomisation, if used. |  |  |
| Allocation concealment mechanism | 9 | Specification that allocation was based on clusters; description of any methods used to conceal the allocation from the clusters until after their recruitment. |  |  |
| Implementation | 10a | Who generated the random allocation schedule, who enrolled clusters, and who assigned clusters to sequences of treatments in the schedule. |  |  |
|  | 10b | Mechanism by which individual participants were included in clusters for the purposes of the trial (such as complete enumeration or random sampling; continuous recruitment or ascertainment, or recruitment at a fixed point in time), including who recruited or identified participants. |  |  |
|  | 10c | Whether consent was sought, from whom, when and for what; whether this differed between treatment conditions. Justification for any waiver or modification of informed consent requirements. |  |  |
| Blinding | 11a | Who was blinded after assignment to sequences (e.g. cluster level participants, individual level participants, those assessing outcomes) and how. |  |  |
|  | 11b | If relevant, description of the similarity of interventions. |  |  |
| Statistical methods | 12a | Target estimand for each primary and secondary outcome including whether it pertains to the cluster-level or individual-level; statistical methods for their estimation including how period effects, clustering and repeated measures were taken into account. Any assessment of carryover effects should be reported. |  |  |
|  | 12b | Methods for additional analyses, such as subgroup analyses, sensitivity analyses, and adjusted analyses. |  |  |
| **Results** |  |  |  |  |
| Participant flow (a diagram is strongly recommended) | 13a | The numbers of clusters that were assessed for eligibility and were randomly assigned to each sequence. For each sequence-period (i.e. each cell) or treatment condition:   - the numbers of clusters that received intended treatments and were analysed for the primary outcome; and, - the numbers of participants who were assessed for eligibility, received intended treatments and were analysed for the primary outcome. |  |  |
|  | 13b | For each sequence-period (i.e. each cell) or treatment condition, losses and exclusions for both clusters and participants with reasons. |  |  |
| Recruitment | 14a | Dates of treatment periods and washout periods. |  |  |
|  | 14b | Why the trial ended or was stopped. |  |  |
| Baseline data | 15 | A table showing baseline cluster level characteristics by sequence, and individual level characteristics for each sequence-period (i.e. each cell) or treatment condition. |  |  |
| Numbers analysed | 16 | The number of observations and clusters included in each analysis for each treatment condition and whether the analysis was according to the allocated schedule. |  |  |
| Outcomes and estimation | 17a | For each primary and secondary outcome, summary statistics by sequence-period (i.e. each cell) or treatment condition; the estimated effect size and its precision (e.g. 95% confidence interval); and any within-cluster correlations or variance components estimated in the analysis. |  |  |
|  | 17b | For binary outcomes, presentation of both absolute and relative effect sizes is recommended. |  |  |
| Ancillary analyses | 18 | Results of any other analyses performed, including subgroup analyses, sensitivity analyses, and adjusted analyses, distinguishing pre-specified from exploratory. |  |  |
| Harms | 19 | Important harms or unintended effects in each treatment condition (for specific guidance see CONSORT Harms 2022 statement). |  |  |
| **Discussion** |  |  |  |  |
| Limitations | 20 | Trial limitations, addressing sources of potential bias, imprecision, and if relevant, multiplicity of analyses. Consider potential carry-over effects. |  |  |
| Generalisability | 21 | Generalisability (external validity, applicability) of the trial findings. Generalisability to clusters or individual participants, or both (as relevant). |  |  |
| Interpretation | 22 | Interpretation consistent with results, balancing benefits and harms, and considering other relevant evidence. |  |  |
| **Other information** |  |  |  |  |
| Registration | 23 | Registration number and name of trial registry, or state the trial was not registered. |  |  |
| Protocol | 24 | Where the full trial protocol and statistical analysis plan can be accessed, if available. |  |  |
| Funding | 25 | Sources of funding and other support (such as supply of drugs), role of funders. |  |  |
| Research ethics review | 26 | Whether the study was approved by a research ethics committee, with identification of the review committee(s). |  |  |
| Data sharing | 27 | Where the individual de-identified participant data (including data dictionary), statistical code, and any other relevant documents/materials can be accessed. |  |  |
| Patient and public involvement | 28 | Details of any patient and/or public involvement in the design, conduct and reporting of the trial; and, when applicable, other stakeholders’ involvement. |  |  |

Cite as: McKenzie JE, Taljaard M, Hemming K, et al. Reporting of cluster randomised crossover trials: extension of the CONSORT 2010 statement with explanation and elaboration. *BMJ* 2025;388:e080472. doi:10.1136/bmj-2024-080472

* Completing section headings may be preferable to page numbers in some circumstances given headings are more likely to be persistent throughout the peer review process.
